# Loss of intestinal Akt elicits rapid mortality via intestinal damage which is rescued by mTORC1 activation

**DOI:** 10.1038/s41467-026-77283-9

**Published:** 2026-09-23

**Authors:** Ahmed Magdy, Hyunsoo Rho, Muzamil Mushtaq, William Putzbach, Veronique Nogueira, Vaibhav Chand, Nissim Hay

**Affiliations:** 1 Department of Biochemistry and Molecular Genetics, College of Medicine, University of Illinois at Chicago, Chicago, IL USA; 2 Research & Development Section, Jesse Brown VA Medical Center, Chicago, IL USA; 3 Present Address: Department of Cancer Biology, Dana-Farber Cancer Institute, Boston, MA USA; 4 Present Address: Department of Cell Biology, Harvard Medical School, Boston, MA USA; 5 Present Address: College of Pharmacy, Graduate School of Pharmaceutical Sciences, Ewha Womans University, Seoul, Republic of Korea; 6 Present Address: Graduate Program in Innovative Biomaterials Convergence, Ewha Womans University, Seoul, Republic of Korea

**Keywords:** Stem cells, Cancer therapy, Cell signalling

## Abstract

Combined *Akt1* and *Akt2* systemic deletion in adult mice elicits rapid mortality, preceded by body weight loss, diarrhea, and inflammation. Here, we show that lethality induced by systemic *Akt1/Akt2* deletion is caused by intestinal damage, as intestinal *Akt1/Akt2* deletion induces mortality phenocopying systemic deletion. Consistently, Akt ablation impairs intestinal organoid formation in vitro. Both in vivo and in vitro results show that Akt is essential for the survival of Paneth cells and survival and proliferation of Lgr5^+^ and transit-amplifying cells. Additional intestinal deletion of either *FoxO1* or *Tsc1* activates mTORC1 and rescues this phenotype. Thus, mTORC1 emerges as the most critical downstream effector of Akt in maintaining intestinal integrity. Indeed, mTORC1 inhibition by *Raptor* deletion phenocopies intestinal deletion of *Akt1/Akt2*. Remarkably, systemic *Akt1/Akt2* deletion is tolerated when *FoxO1* is deleted concurrently. This study unravels a critical role for Akt in maintaining intestinal homeostasis and the interplay between its downstream effectors FoxO1 and mTORC1.

## Abstract (web summary)

Akt is required for intestinal integrity, and Akt1/Akt2 intestinal deletion induces rapid mortality in mice, which is recapitulated by pan-PI3K and Akt inhibitors, and is reversed by activating mTORC1 via additional deletion of either FoxO1 or Tsc1.

## Introduction

The three Akt isoforms (Akt1-3) encoded by separate genes are almost identical to each other with respect to their amino acid sequences. However, they are expressed at relatively different levels across various mammalian tissues. Akt1 is the most ubiquitously expressed isoform, whereas Akt2 is the major expressed isoform in insulin-responsive tissues such as skeletal muscle, adipose tissue, and liver, and Akt3 is primarily expressed in the brain. Consequently, Akt1 null mice display impaired overall growth^1,2^, whereas Akt2-deficient mice display hyperinsulinemia and are insulin resistant^3^, consistent with the highest expression of Akt2 in insulin-responsive tissues. *Akt3*^*−/−*^ mice display a small-sized brain^4,5^, consistent with the highest expression of Akt3 in the brain. All Akt single isoform knockout mice are viable; however, combined deletions of (Akt1/Akt3) and (Akt1/Akt2) are prenatal and neonatal lethal, respectively^6,7^. Although the systemic deletion of Akt1 and Akt3 in adult mice did not have an overt phenotype, systemic deletion of Akt1 and Akt2 in adult mice elicited rapid mortality^8^.

It remains to be determined why systemic Akt1 and Akt2 deletion in adult mice elicits rapid mortality. Given our observations of intestinal damage and high inflammation together with the loss of body fat prior to death, we hypothesized that the reason for the mortality is intestinal damage that prevents nutrient absorption and markedly increases systemic inflammation by the disruption of the epithelial barrier. As an alternative, the mice consume body fat, and once it is exhausted, they succumb to death. If this hypothesis is correct, we should also expect rapid mortality after intestinal *Akt1* and *Akt2* deletion. Here we showed that intestinal deletion of *Akt1* and *Akt2* phenocopied systemic deletion. Our results revealed that Akt is essential for intestinal homeostasis and is particularly required for the survival and differentiation of rapidly cycling stem cells, as well as for suppressing cell death.

The intestinal epithelium is considered one of the most regenerative tissues in adult mammals and is maintained by self-renewing and rapidly proliferating Lgr5⁺ crypt-based columnar (CBC) stem cells^9^. Lgr5⁺ stem cells give rise to all differentiated cell types every 4–5 days. They divide continuously to generate Paneth cells, transit-amplifying (TA) progenitors, and self-renewing stem cells. Paneth cells are long-lived, and one of their functions is to support the stem cells. While Paneth cells reside at the base of the crypt, TA cells undergo rapid expansion along the crypt–villus axis and differentiation into two main lineages: absorptive enterocytes and secretory cell types, including goblet, enteroendocrine, and tuft cells^10^. Intestinal stem cell identity, proliferation, and lineage commitment are maintained by a plethora of signaling pathways, such as the WNT and Notch pathways^10,11^. In addition to these canonical pathways that regulate the balance between stemness and differentiation, accumulating evidence indicates that in response to hormones, growth factors, and nutrient cues, the PI3K/AKT signaling pathway plays an important role in intestinal epithelial cell fate^12–18^. AKT signaling is increasingly recognized as a key modulator of intestinal stem cell function and intestinal homeostasis mainly through its evolutionarily conserved effectors FOXO and mTORC1^13,19–21^.

We found that Akt is required for Paneth cell survival and for the survival and proliferation of Lgr5^+^ and transit-amplifying cells. Because of intestinal damage, the intestinal barrier was disrupted, leading to inflammation. The results have implications for the treatment of cancer patients with pan-Akt or pan-PI3K inhibitors that can also induce intestinal damage and mortality in mice, which is consistent with the severe diarrhea often observed after treatment of patients with these inhibitors^22,23^. Akt has multiple downstream effectors^24^. However, we focused on the most evolutionarily conserved targets, the transcription factor FOXO (FOXO1, 2, 3) and mTORC1. FOXO is phosphorylated by Akt, leading to its nuclear exclusion and degradation, whereas mTORC1 is activated by Akt largely via phosphorylation and inactivation of TSC2, which heterodimerizes with TSC1 to inhibit mTORC1^25^. Interestingly, the effects of Akt deletion on the intestine were recovered by additional deletion of *FoxO1* or *Tsc1*. Either FoxO1 or Tsc1 deletion elevated mTORC1 activity, suggesting that mTORC1 downstream of Akt is critical for intestinal homeostasis. Indeed, the phenotype of intestinal Raptor deletion, which inhibits mTORC1, recapitulated the phenotype of intestinal Akt1 and Akt2 deletion. Surprisingly, combined systemic deletion of *Akt1* and *Akt2* and *FoxO1* did not elicit intestinal damage or rapid mortality, suggesting that adult mice can survive in the absence of Akt1 and Akt2 if *FoxO1* is also systemically deleted. These mice were viable and active, but displayed hyperglycemia, hyperinsulinemia, and lipodystrophy.

## Results

### The phenotype of inducible intestine-specific Akt1 and Akt2 deletion recapitulates the phenotype of systemic Akt1 and Akt2 deletion

It was previously shown that systemic deletion of Akt1 and Akt2 in adult mice elicits rapid mortality^8^. Since previous experiments were done in the FVB background we repeated the experiments more extensively in C57BL/6 mice (Fig. S1). *Akt1*^*f/f*^; *Akt2*^*f/f*^; *R26Cre*^*ERT2*^ mice expressing floxed alleles of *Akt1* and *Akt2* and tamoxifen (Tam)-activated *Cre* recombinase (*Cre*^*ERT2*^*)* in the ubiquitous Rosa26 (R26) locus were generated in C57BL/6 background. The mice were exposed to Tam for 7 consecutive days to systemically delete Akt1 and Akt2 as previously described^8^. Deletion of Akt1 and Akt2 was verified in all tissues tested (Fig. S1a). Both male and female mice started losing body weight 4 days after exposure to Tam (Fig. S1b) followed by mortality (Fig. S1c). IL-6 levels measured in the serum of Akt null mice were dramatically increased (Fig. S1d), food consumption was markedly decreased (Fig. S1e), and the mice became hypoglycemic (Fig. S1f). Gross examination showed watery intestine and diarrhea (Fig. S1g). These mice also depleted adipose tissues suggesting increased fat consumption and lipolysis (Fig. S1g, h). The length of both the small and large intestines was significantly reduced (Fig. S1i). Finally, cross sections of the intestine and colonic crypts showed smaller and disrupted villi accompanied by colonic epithelial surface flattening (Fig. S1j, k). Staining for Ki67 and cleaved caspase-3 (CC3) showed that proliferation was inhibited, and cell death was increased after systemic Akt1 and Akt2 deletion, respectively (Fig. S1l, m). We hypothesized that the cause of mortality induced by systemic Akt1 and Akt2 deletion is the intestinal damage, followed by disruption of the epithelial barrier and systemic inflammation, as measured by IL-6 levels in the blood. The intestinal damage precludes the ability to absorb food leading to nutrient malabsorption, and therefore, body fat is being used and once exhausted the mice display severe hypoglycemia culminating in mortality. Therefore, we should expect that intestinal deletion of Akt1 and Akt2 would cause the same phenotype.

**Fig. 1:**
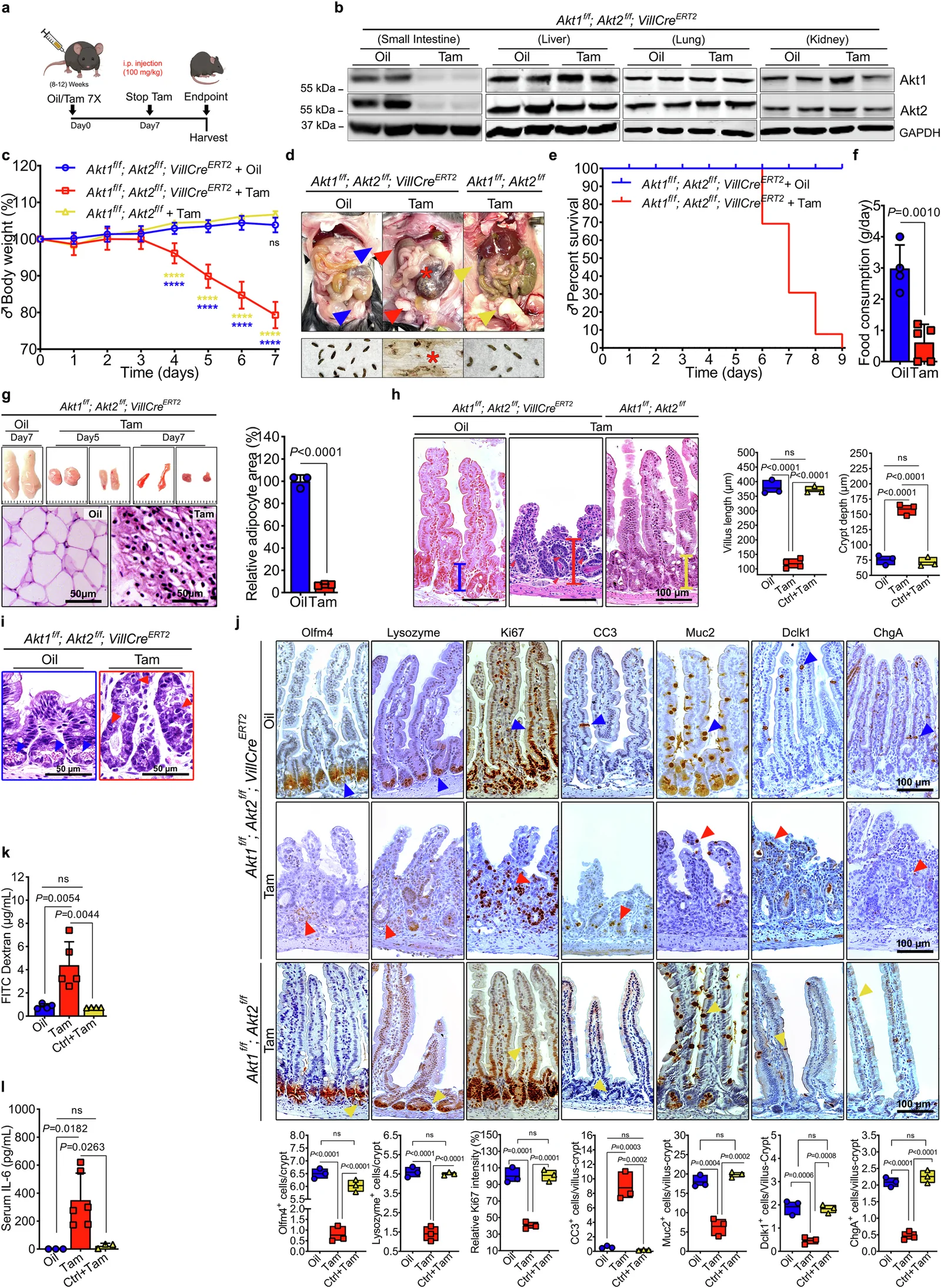
Intestine specific deletion of *Akt1* and *Akt2* in adult mice elicits intestinal damage leading to rapid mortality. **a** Schematic representation of our experimental protocol. Male C57BL/6J mice were injected with corn oil or tamoxifen for 7 consecutive days. Created in BioRender: https://BioRender.com/qc70ibv. **b** Immunoblotting from the indicated tissue lysates after treatment. GAPDH is a loading control. (*n* = 3 independent experiments). **c** Changes in body weight. (*n* = 10, 15, 10). Mean ± SD. Statistical analysis was performed using a mixed-effects model (Tukey’s post-hoc multiple comparisons test). ****P  ≤  0.0001, ns, not significant. **d** Abdominal photograph of representative animals at endpoint, showing white adipose tissue (WAT) depots indicated by colored arrowheads (upper panel), and normal stool versus diarrhea, marked by red asterisks, (lower panel). (*n* = 3). **e** Kaplan–Meier plot (*n* = 10). Log-rank (Mantel-Cox) test. **f** Food intake at endpoint (g/day). (*n* = 4, 5). Mean ± SD. **g** Representative images of gWAT (upper), H&E staining (lower), and quantification (Right). (*n* = 3 mice, 5 sections each). Mean ± SD. **f**, **g** Unpaired two-tailed *t*-test. **h** Representative H&E images showing changes in morphometric parameters of the small intestine. The red arrowheads indicate apoptotic and necrotic cells. The colored scale bars define the crypt area and illustrate differences in crypt thickness (Left). Quantification (Right). (*n* = 3). **i** Representative images of H&E-stained sections showing elongated crypts, necrotic cells in Tam-treated mice only (red arrowheads), and Paneth cells secretory granules in oil-treated mice only (blue arrows). (*n* = 3). **j** Representative images of immunohistochemistry (IHC) staining showing morphological and histological changes (upper), and quantification, (lower). **h**, **j** floating bars show minimum, mean, and maximum. (*n* = 3 per group). Quantification from “Tam” and “Ctrl + Tam” are used again later in (Figs. 4i and 6i). **k** Plasma concentrations of fluorescein isothiocyanate (FITC)- dextran. (*n* = 4, 5, 4), performed in technical duplicate. **l** Serum IL-6 levels show a robust induction of IL-6 after intestinal deletion of Akt. (*n* = 3, 6, 3), technical duplicate. **h**, **j**, **k**, **l** one-way ANOVA (Tukey’s post-hoc test). Source data are provided as a Source Data file.

To test this hypothesis, we generated a mouse model with intestine-specific deletion of Akt1 and Akt2. First, *Akt1*^*f/f*^ and *Akt2*^*f/f*^ mice were bred with mice expressing *Cre*^*ERT2*^ under the control of *Villin-1* promoter^26^. After multiple crossings of heterozygous mice, the *Akt1*^*f/f*^; *Akt2*^*f/f*^; *VillCre*^*ERT2*^ homozygous mouse strain was generated and maintained. Control groups included *Akt1*^*f/f*^; *Akt2*^*f/f*^; *VillCre*^*ERT2*^ mice treated with corn oil, *Akt1*^*f/f*^; *Akt2*^*f/f*^ mice treated with Tam, and *VillCre*^*ERT2*^ mice receiving Tam. *Akt1*^*f/f*^; *Akt2*^*f/f*^; *VillCre*^*ERT2*^ and control mice were treated at 8–12 weeks with Tam or oil, for 7 consecutive days (Fig. 1a). The deletion of Akt exclusively in the intestine was confirmed by western blot analysis using isoform specific antibodies (Fig. 1b). As expected, unlike R26-induced Cre^ERT2^ (Fig. S1a), VillCre^ERT2^ efficiently and specifically deleted the floxed *Akt1* and *Akt2* alleles in the intestine. The protein levels of both Akt isoforms in the liver, lung and kidney of Tam-treated mice were unchanged in comparison to oil-treated mice (Fig. 1b). Indeed, Akt1 and Akt2 are ubiquitously expressed in the intestine while Akt3 is not (Figs. 1b and S2a). Remarkably, 3–4 days post Tam injection, mice lacking Akt specifically in the intestine appeared inactive. Conversely, oil injected mice were normal, as were the Cre-free mice injected with Tam. Notably, mice from both control groups maintained their normal physical activity and body weight, with only minimal fluctuations observed. However, 5–7 days post-injection, intestinal Akt-deficient mice exhibited reduced activity, hunched posture, and lethargy. Additionally, these mice had a dramatic reduction in their body weight (Fig. 1c) as observed with Akt1 and Akt2 systemic deletion (Fig. S1b), which also associated with induction of severe and bloody diarrhea and dehydration. Namely, small and large intestines from mice deficient in intestinal Akt were pale and watery, and the cecum was full of bloody fluid (Fig. 1d), suggesting both disruption of intestine absorptive function and induction of tissue injury and inflammation.

Strikingly, 100% of mice lacking intestinal Akt exhibited rapid mortality within only 7 to 10 days, whereas control littermates appeared overtly normal (Fig. 1e). Therefore, these results suggest that Akt plays an essential role in regulating intestinal homeostasis, and upon its loss, intestinal tissue exhibits pathological changes. Notably, no differences were observed between males and females regarding body weight kinetics or survival, in both the systemic (Fig. S1b, c) and intestinal Akt deletion models (Figs. 1c, e and S2b, c). Indeed, we observed that structural abnormalities following Akt deletion were accompanied by a concomitant reduction in food intake (Fig. 1f). Moreover, nutrient uptake, as assessed by the number of lipid droplets in jejunal enterocytes after exposure to a high-fat diet (HFD) for 24 h (Fig. S2d), and the levels of glucose and lipids measured in the bloodstream at endpoint were significantly decreased (Fig. S2e). We also noticed that mice with intestinal deletion of Akt showed depletion of adipose tissues, particularly gonadal white adipose tissue (gWAT), in a time-dependent manner (Fig. 1d, g). These findings imply that mice lacking intestinal Akt are unable to effectively utilize external nutrients and may use body fat instead.

Next, we investigated the architecture of the small intestine and colon. Jejunal sections from intestinal Akt-deficient and Akt-proficient mice were stained with H&E and examined microscopically. In contrast to the control group that had normal morphology with no obvious architectural changes, the structure of villi and crypts from Akt-ablated mice was severely disrupted (Fig. 1h). Specifically, villi from Akt-intestinal deficient mice appeared sparse and blunted. Moreover, many epithelial cells in these villi and crypts were necrotic and detached (Fig. 1h). Furthermore, the crypts and crypt area were markedly elongated compared to the control group (Fig. 1h, i). These results suggest a lack of differentiation accompanied by induction of cell death and tissue damage, mediated by Akt loss. Similarly, the colonic epithelium showed a significant reduction in crypt depth, with the surface of epithelium appearing flattened and invaginations of the crypts being aberrantly diminished (Fig. S2f). These morphological changes suggest that Akt plays a crucial role in maintaining the structural integrity of the small and large intestines and that Akt deletion results in severe structural distortions within the gut. Taken together, these results support our hypothesis and show that mice with intestinal Akt1 and Akt2 deletion phenocopy the systemic Akt1 and Akt2 deletion phenotype. Thus, the intestinal damage could explain the rapid mortality of the mice following systemic Akt1 and Akt2 deletion.

### Akt is essential for intestinal tissue homeostasis and epithelial barrier integrity

The results described above clearly show that Akt is required for intestinal homeostasis and its ablation in the intestine disrupts intestinal integrity and promotes lethality. These observations prompted us to address in detail the question of how intestinal deletion of Akt impaired intestinal homeostasis; as indicated by the distorted architecture of villi and crypts, diminished nutrient uptake, and increased inflammatory response. It is well-established that regeneration of intestinal architecture and sustainable maintenance of the gastrointestinal mucosa is largely reliant on a constant pool of Lgr5^+^ stem cells nestled at the crypt base^9,27–29^. Lgr5^+^ adult stem cells, which are also marked by Olfm4, Sox9, and Ascl2^30–32^ not only continuously maintain themselves and give rise to their neighboring differentiated cells, Paneth cells, but also continually proliferate to supply a multipotent and highly proliferative population of cells upward in the villus known as transit-amplifying cells (TACs). TACs, in turn, differentiate into their progeny including nutrient-absorbing enterocytes, mucus-secreting goblet cells, chemosensory and immune sentinels (tuft cells), and hormone-secreting enteroendocrine cells, leading to the generation of all epithelial cell lineages comprising the intestinal epithelium in approximately 4–5 days^9,27,33,34^.

Hence, we set out to determine whether Akt ablation in the intestine had any impact on the maintenance of intestinal stem cells and other differentiated cell types. We performed RNA-seq on bulk mRNA followed by pathway analysis. Interestingly, gene expression analysis of regulators and cell type-specific markers of stem cells and all distinct cell lineages suggested that loss of Akt compromised intestinal homeostasis (Fig. S2g and Supplementary Data 1). Our results demonstrated a significant reduction in the gene expression levels of all stem cell and differentiation markers. Transcriptomic analysis of the two major signaling pathways (Wnt and Notch) governing intestinal stemness, progenitor expansion, and differentiation commitment were downregulated by *Akt1/Akt2* deletion. Specifically, mRNAs of Wnt related genes such as *Fzd2*, *Wnt6*, *Wnt9b*, *Lypd6*, and *Dand5* and of Notch target genes such as *Nrarp*, *Dtx1*, *Rfng*, and *Hes2* were markedly reduced, suggesting a key role for Akt in modulating these two pathways in the small intestine (Fig. S2g and Supplementary Data 1).

IHC staining showed that Olfm4 (intestinal stem cells (ISCs)) and Lysozyme (Paneth cells) were markedly diminished by *Akt1/Akt2* deletion (Fig. 1j), strongly indicating that Akt deficiency eliminates stem cells and Paneth cells. Since Paneth cells are long-lived and can survive for up to 57 days^35^, we concluded that Akt is essential for the viability of Paneth cells. Additionally, TACs in these mice displayed a decline in proliferative capacity and increased cell death compared with control groups, detected by a significant reduction in Ki67 and increased CC3 staining, respectively (Fig. 1j). Furthermore, absorptive enterocytes, and secretory goblet cells (Muc2), tuft cells (Dclk1), and enteroendocrine cells (ChgA) were markedly reduced (Fig. 1j). Collectively, these results suggest that the loss of intestinal Akt impairs cell survival and proliferation of cells giving rise to differentiated lineages.

Next, we hypothesized that cell death triggered by Akt intestine specific deletion disrupted intestinal barrier function and tissue integrity leading to systemic inflammation. To investigate this possibility, we performed the widely accepted intestinal permeability assay using FITC-Dextran^36^. Notably, 4 h after it was orally administered, the concentration of the 4-kDa FITC dextran measured in plasma was significantly higher in Akt-deficient mice compared to controls (Fig. 1k). This result indicates increased intestinal permeability and compromised epithelial barrier function. Given the crucial role of barrier integrity in protecting against systemic inflammation^37^, we analyzed inflammation-related genes significantly upregulated upon Akt deletion. RNA-seq analysis (Fig. S2h and Supplementary Data 2) revealed a strong induction of inflammatory signaling. Notably, KEGG pathway analysis of genes significantly upregulated in Akt deleted mice (Fig. S2i and Supplementary Data 3) revealed a robust activation of the innate inflammatory program, dominated by NF-κB, TNF-α, IL-1, TLR, and neutrophil-associated signaling. Focused analysis of NF-κB-related transcripts further supported induction of inflammatory response and immune cell infiltration (Fig. S2j and Supplementary Data 4). Consistently, IHC staining demonstrated infiltration of various immune cells including neutrophils, macrophages, T cells, and dendritic cells, within the affected mucosa (Fig. S2k). In addition, three independent experiments demonstrated a marked increase in the number of bacteria isolated from ileal luminal contents of inflamed intestines compared to the control group, suggesting impaired antimicrobial defense in Akt-deficient mice (Fig. S2l). These findings coincided with increased circulating IL-6 levels (Fig. 1l). Thus, impaired intestinal regeneration caused by Akt deletion not only decreases nutrient uptake but also compromises the anti-microbial defense against luminal antigens and bacteria, which may contribute to increased IL-6 observed in these mice.

### Akt is essential for crypt regeneration, and Lgr5^+^ stem cell self-renewal in vitro

**Fig. 2:**
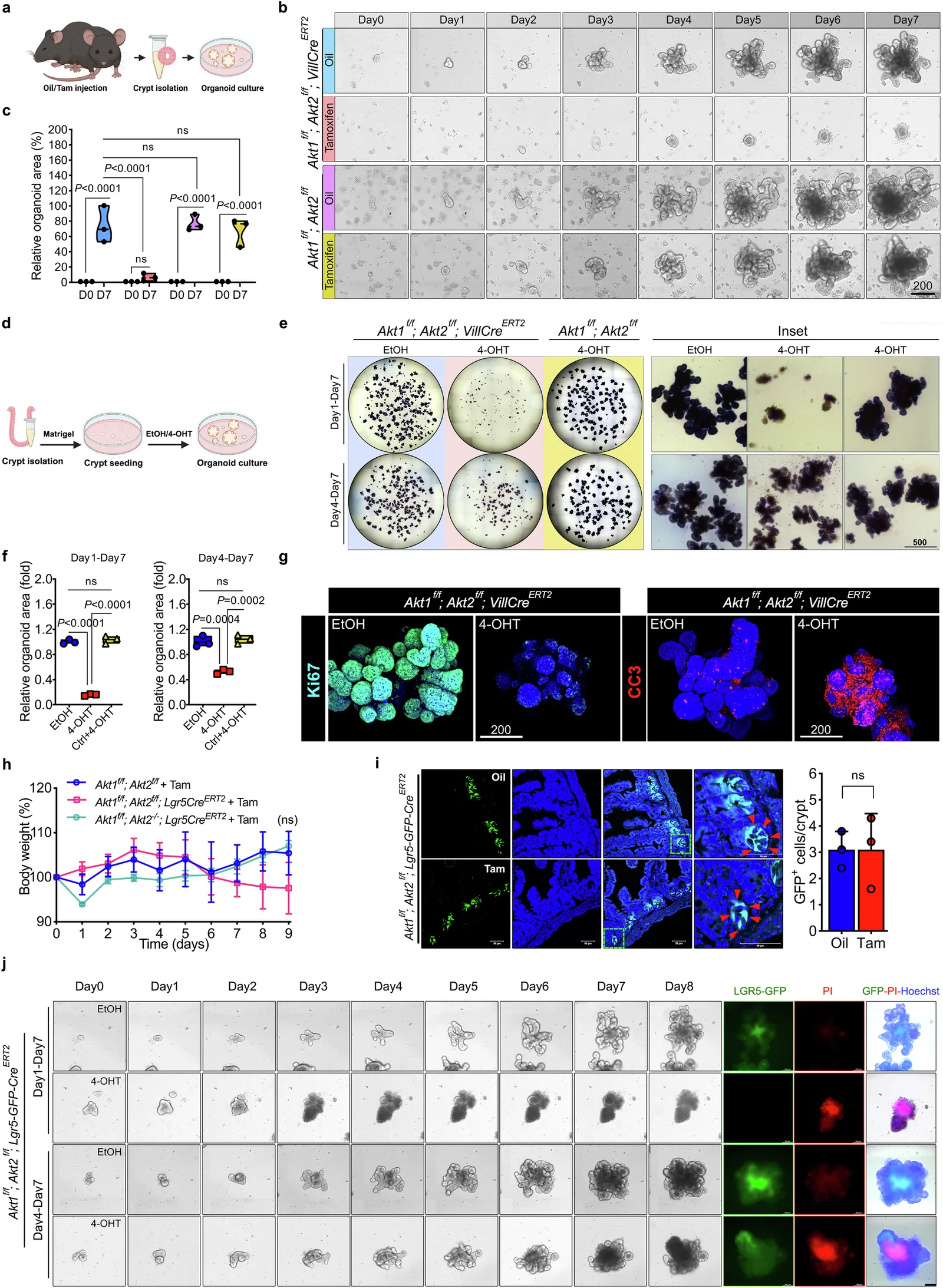
Intestinal Akt1 and Akt2 deletion impairs organoid formation and viability. **a** Schematic representation of the experimental workflow. Created in BioRender: https://BioRender.com/qc70ibv. **b** Representative bright-field images of primary organoid growth, and **c** quantification of relative organoid area, shown as percent at Day 7 in comparison to Day 0. Results demonstrate a complete loss of organoid forming capacity in Akt-deficient crypts compared to control groups. (*n* = 3 independent biological experiments). Data are plotted as individual points on truncated violin plots representing the minimum, median, and maximum. Two‑way repeated‑measures ANOVA (Sidak’s multiple‑comparisons test). **d** Experimental design for secondary organoid regeneration. Organoids were treated in vitro with 4-OHT (2 µM) to induce Cre activity, or EtOH as a vehicle. Created in BioRender: https://BioRender.com/qc70ibv. **e** MTT staining of organoids on Day 7 reveals structural and morphological changes in Akt-deficient compared with Akt-proficient organoids. (left: overview; right: insets). **f** Quantification of relative organoid area demonstrates a marked reduction in the ability of organoids to grow and regenerate in culture following incubation with 4-OHT, compared to control groups. Results are representative of three independent experiments. Floating bars show minimum, mean, and maximum. One-way ANOVA (Tukey’s post-hoc test). (ns, (*P* > 0.05); exact *P*-values are indicated on the graph). **g** Immunofluorescence staining for (Ki67, proliferation) and (CC3, apoptosis) showing reduction of proliferation and induction of cell death in organoids treated with 4-OHT compared to vehicle. Three independent biological replicates. **h** Body weight changes 9 days post Tam treatment demonstrate no significant difference. (*n* = 10, 9, 5). Mean ± SD. One-way ANOVA (Tukey’s post-hoc test). **i** Representative immunofluorescence images of GFP expression in small intestine sections of (*Akt1*^*f/f*^; *Akt2*^*f/f*^; *Lgr5-GFP-IRES-Cre*^*ERT2*^) mice following treatment (left). Quantification of Lgr5-GFP^+^ cells is shown on the right. (*n* = 3). (Unpaired two-tailed *t*-test). **j** Representative bright-field with side-by-side fluorescence images of (*Akt1*^*f/f*^; *Akt2*^*f/f*^; *Lgr5-GFP-IRES-*Cre^ERT2^) organoids. Fluorescence channels: GFP (green, Lgr5^+^ stem cells), propidium iodide (red, dead cells), and Hoechst (blue, nuclei). (*n* = 3 independent experiments). Source data are provided as a Source Data file.

We tested whether the stem cells in any residual crypts isolated from Akt-depleted small intestine can generate organoids in culture^38–43^. We isolated crypts from the duodenum of intestinal Akt-deficient mice 7–10 days post Tam injection, as well as from control groups, see schematic (Fig. 2a). Although a small number of Akt-lacking crypts appeared normal and intact during tissue digestion, none of these Akt-devoid crypts were able to generate primary organoids (Fig. 2b). In contrast, brightfield microscope images, acquired daily and for up to 7 days, demonstrated that organoids from control groups had a significant increase in their area, compared to their initial area (Figs. 2b, c).These results support a model in which stem cells from intestinal Akt-deficient mice have lost their ability to regenerate in vivo, and that Akt-proficient stem cells are essential for forming intestinal organoids and expanding in vitro. Collectively, these data strongly suggest that conditional deletion of Akt in the intestinal epithelium negatively regulates Lgr5^+^ stem cell regeneration. Subsequently, the loss of Akt in intestinal stem cells negatively impacted the self-renewal capacity of the crypt-villus axis, impaired organoid formation, compromised differentiation to all terminally differentiated epithelial cells, and ultimately perturbed tissue homeostasis. Similarly, we isolated crypts from *Akt1*^*f/f*^; *Akt2*^*f/f*^; *VillCre*^*ERT2*^ and *Akt1*^*f/f*^; *Akt2*^*f/f*^ mice, mixed with Matrigel, and incubated in culture to form organoids. Organoids from both genotypes were expanded for at least two passages before they were seeded for organoid formation and cell viability assay. To induce Cre recombinase activity and Akt deletion in vitro, we added to the media 2 µM of 4-hydroxytamoxifen (4-OHT) at two different time points (Day 1 and Day 4), and ethanol (EtOH)-treated organoids were used as a control (Fig. 2d). One week after initiation of the 3D culture, organoids were stained with MTT staining. Images of viable organoids (stained) versus dead (unstained) were obtained using a brightfield microscope and were quantified (Fig. 2e). Interestingly, exposure to 4-OHT on day 1 completely abrogated organoid formation and resulted in nearly complete cell death. Additionally, treating already established organoids on Day 4 with 4-OHT for 3 days had a negative effect on cell survival and expansion (Fig. 2e), with markedly diminished organoid area (Fig. 2e, f). Consistently, staining for Ki67 and CC3 clearly showed diminished proliferation and enhanced cell death in organoids after 4-OHT treatment in comparison to EtOH treatment (Fig. 2g). Collectively, these results underscore an essential role for Akt in the initiation and maintenance of intestinal organoids and further confirm its importance in intestinal epithelial cell survival, proliferation, and homeostasis.

To further confirm that Akt is indispensable for cell-intrinsic stem cell survival, self-renewal, and intestinal homeostasis, we employed a genetic approach to specifically ablate Akt exclusively in Lgr5^+^ stem cells. To this end, we crossed a transgenic mouse model expressing a knock-in *Lgr5-EGFP-IRES-Cre*^*ERT2*^ allele, which has been previously described and widely used^9,29,44–47^, with a mouse strain carrying *Akt1* and *Akt2* floxed alleles. We ultimately generated two mouse strains with conditional stem cell-restricted Akt deletion upon Tam injection (*Akt1*^*f/f*^*; Akt2*^*f/f*^*; Lgr5-EGFP-IRES-Cre*^*ERT2*^ and *Akt1*^*f/f*^*; Akt2*^*-/-*^*; Lgr5-EGFP-IRES-Cre*^*ERT2*^). To determine whether specific deletion of Akt exclusively in Lgr5^+^ cells will have any effect on intestinal regeneration and homeostasis, we injected these mice with Tam or oil for 7 days. Surprisingly, the changes in body weight between Tam-injected and oil-injected mice were comparable (Fig. 2h). Furthermore, quantification of Lgr5-EGFP^+^ cells from small intestine tissues using a confocal microscope revealed no significant differences between the two groups of mice (Fig. 2i), suggesting that Lgr5^+^ stem cell populations remain unaffected. Namely, the mice remained active, maintained their body weight within physiological ranges, and exhibited no signs of pathological changes or mortality. Collectively, the observations we had earlier from the VillCre^ERT2^ system were not recapitulated by this Lgr5Cre^ERT2^ model. Notably, the same observations regarding the discrepancies between Villin-driven and Lgr5-driven Cre systems were previously reported in several studies^48–50^. Specifically, it was shown that Lgr5^+^ cells are regenerated from other cells that are de-differentiated^46,51,52^.

To directly examine the role of Akt in modulating proliferation and survival of Lgr5^+^ cells, we utilized the in vitro epithelial-only organoid culture and 4-OHT treatment to activate Cre recombinase. We isolated crypts from *Akt1*^*f/f*^*; Akt2*^*f/f*^*; Lgr5-EGFP-IRES-Cre*^*ERT2*^ mice and incubated them with either EtOH or 4-OHT. Interestingly, microscopic images demonstrated a significant reduction in the area of organoids generated from Lgr5^+^ stem cells deficient in Akt compared to control (Fig. 2j). Administration of 4-OHT immediately after seeding completely precluded organoid formation and induced cell death within three days. Adding 4-OHT at day 4 after organoids have established, also nearly eliminated organoids 3–4 days later (Fig. 2j). Moreover, the frequency of Lgr5^+^ cells in the organoids was indirectly evaluated by measuring GFP fluorescence intensity (Fig. 2j), and consistently incubation with 4-OHT at the endpoint diminished GFP fluorescence. Therefore, we confirm that the impaired regeneration and compromised intestinal integrity phenotype in organoids is a direct consequence of losing Lgr5^+^ stem cells. These results strongly suggest that Akt is required for Lgr5^+^ stem cell survival in vivo and demonstrate that this population of stem cells is essential for organoid formation and crypt homeostasis at least in vitro. In summary, we uncovered a previously unrecognized role for Akt in regulating Lgr5^+^ stem cells and intestinal homeostasis.

### Pharmacological inhibition of PI3K or Akt mimics the consequences of *Akt* genetic deletion

**Fig. 3:**
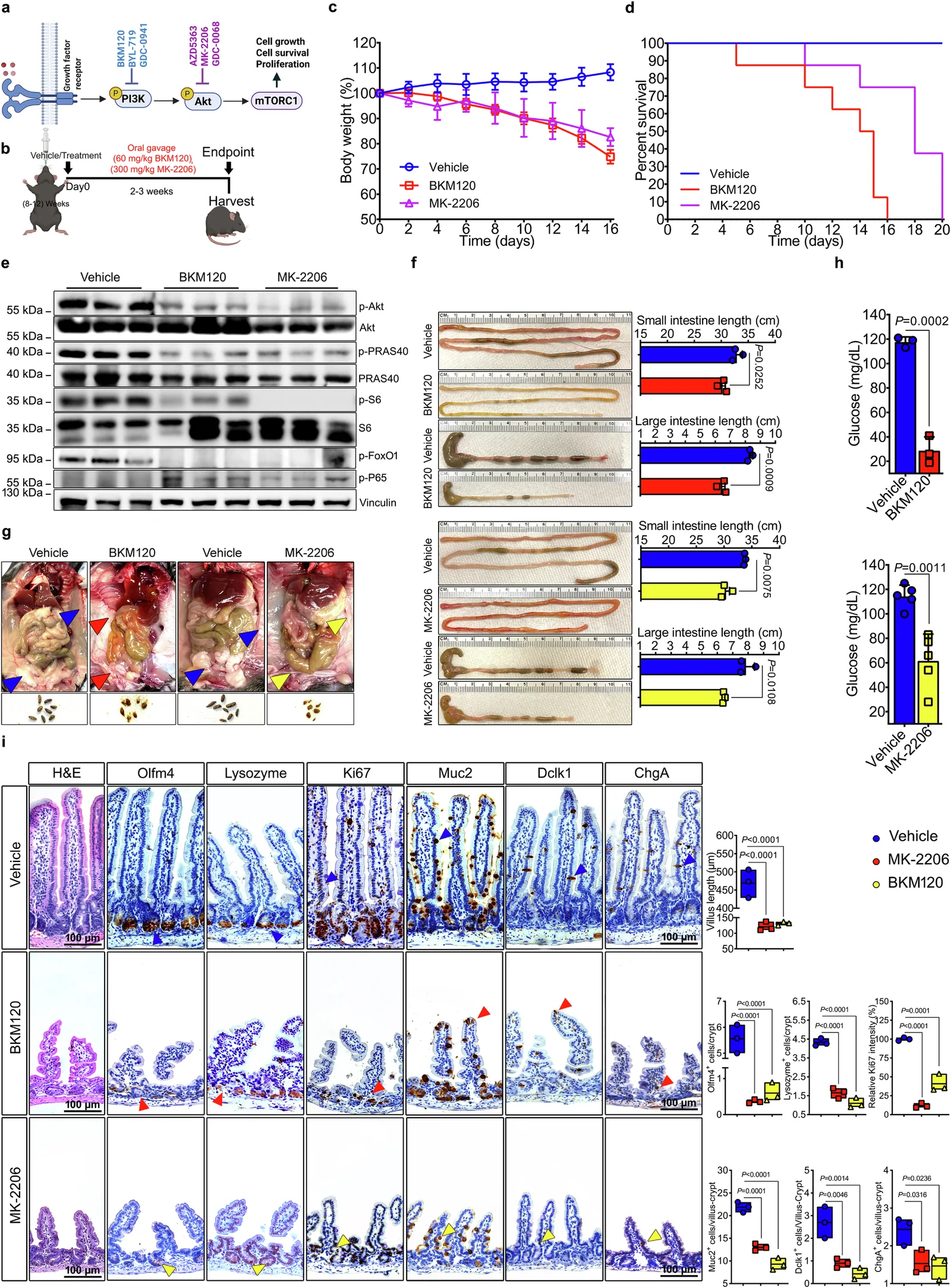
Inhibition of PI3K/AKT recapitulates the consequences of Akt genetic ablation. **a** Schematic of the PI3K/Akt pathway regulation and inhibitors used in this study. Created in BioRender: https://BioRender.com/ia6gmu4. **b** Schematic representation of the experimental protocol. WT male mice were orally administered vehicle, BKM120 (pan-PI3K inhibitor) or MK-2206 (pan-Akt inhibitor) for 2–3 weeks. Created in BioRender: https://BioRender.com/lbqo95f. **c** Body weight changes in mice treated daily for 2 weeks with (BKM120, 60 mg/Kg) compared to vehicle (10% DMSO + 40% PEG300 + 5% Tween-80 + 45% PBS), and day-on, day-off for 3 weeks with (MK2206, 300 mg/kg) in comparison to vehicle (Captisol). (*n* = 9, 7, 8). Mean ± SD. Statistical analysis was performed using a mixed-effects model (Tukey’s post-hoc multiple comparisons test). (ns, *P* > 0.05; *P ≤ 0.05; **P ≤ 0.01; ***P ≤ 0.001; ****P ≤ 0.0001). Exact *P* values are provided in the Source Data File. **d** Kaplan–Meier survival plot. (*n* = 8). Log-rank (Mantel-Cox) test. **e** Western blot confirming the inhibition of PI3K/Akt pathway. Membranes were blotted with anti- p-Akt S473, Akt total, p-PRAS40 (Akt target), PRAS40 total, p-S6 (mTORC1 activity readout), S6 total, p-FoxO1, p-P65, and Vinculin (loading control). (*n* = 3 independent biological replicates). **f** Gross intestinal morphology and length measurements (cm). (*n* = 3). Mean ± SD. Unpaired two-tailed *t*-test. **g** Abdominal images showing intestinal and adipose tissue pathological changes (colored arrowheads). (Upper) internal organs including the gut and body fat. (Lower) stool consistency. (*n* = 3). **h** Blood glucose levels reveal hypoglycemia in inhibitor-treated mice (Upper panel: *n* = 3 mice per group; lower panel: *n* = 5 mice per group). Mean ± SD. Unpaired two-tailed *t*-test. **i** Representative H&E and IHC staining of jejunum sections using the indicated antibodies (left) and corresponding quantifications (right). Colored arrowheads highlight phenotypic differences between the groups. (*n* = 3 mice per group). Floating bars show the minimum to maximum range with the internal horizontal line representing the mean. Statistical analysis was performed using one-way ANOVA (Tukey’s post-hoc test). *P* values are indicated on graph. Source data are provided as a Source Data file.

The results described above could explain why patients treated with pan-PI3K or pan-Akt inhibitors occasionally display severe diarrhea and inflammation, which result in discontinuation of the treatment^22,23^. Thus far, we genetically deleted Akt in the intestine and found a wide range of intestinal damage, barrier deficiency, and inflammation. However, we sought to determine whether this could be recapitulated by administrating pan-PI3K or pan-Akt inhibitors. We elucidated the role of the PI3K/Akt signaling pathway in regulating intestinal damage and tissue homeostasis using clinically relevant inhibitors of PI3K and Akt. We utilized panels of pan- (ATP-competitive) and allosteric inhibitors of phosphatidylinositol 3-kinase (PI3K) and Akt, as illustrated in (Fig. 3a). For in vivo work, we treated wild-type (WT) mice by oral gavage with (60 mg/kg) of the PI3Ki BKM120 daily, or with (300 mg/kg) of the Akti MK-2206 every other day (Fig. 3b). Meanwhile, in our in vitro experiments, PI3K inhibitors included BKM120, BYL-719, and GDC-0941, and Akt inhibitors included AZD5363, GDC-0068, and MK-2206 (Fig. S3). First, mice treated with BKM120, or MK-2206 exhibited a significant reduction in their body weight compared to the vehicle-treated mice (Fig. 3c). Importantly, prolonged inhibition of the PI3K/Akt activity (2–3 weeks) significantly reduced mouse viability and increased mortality (Fig. 3d). Consistently, western blot analysis showed a significant reduction in the phosphorylation of Akt and its targets PRAS40 and FoxO1. Additionally, S6 phosphorylation, which is a readout for mTORC1 activity, was also markedly decreased, indicative of an efficient attenuation of the PI3K/Akt pathway (Fig. 3e). Of note, p-p65, a marker of NF-κB activation was markedly increased following PI3K and Akt inhibition. These results are in line with our previous data on NF-κB activation in the genetic model of intestinal Akt deletion (Fig. S2h–j).

Consistent with our previous results and prior reports linking intestinal damage to reduced intestinal length^48,53^, inhibition of the PI3K/Akt pathway significantly reduced the length of both the small and large intestine (Fig. 3f). Furthermore, the gross examination showed mild to severe diarrhea following drug administration compared with mice treated with vehicles (Fig. 3g). These findings from our murine studies align with clinical observation in cancer patients experiencing gastrointestinal intolerance and diarrhea when treated with PI3K or Akt inhibitors^22,23^. Despite these observations, the underlying mechanism for severe diarrhea in cancer patients receiving PI3K/Akt inhibitors remains largely unknown. This highlights a need for further work to understand the role of this pathway in regulating intestinal functions and homeostasis.

Then, we examined whether pharmacological inhibition of PI3K or Akt would compromise the integrity of intestinal mucosa, leading to perturbation of small intestine architecture and function, loss of body fat, and induction of hypoglycemia. Indeed, mice treated with any of the two inhibitors exhibited a complete depletion of gWAT (Fig. 3g), similarly to what we have observed after Akt1 and Akt2 intestine-specific deletion (Fig. 1d, g) and whole-body deletion (Fig. S1g, h). Also, the exhaustion of adipose tissue was followed by hypoglycemia (Fig. 3h). Collectively, pharmacological inhibition of PI3K or Akt recapitulated the effect of Akt1 and Akt2 deletion and impaired nutrient utilization, forcing a reliance on body fat until exhaustion.

Our genetic models demonstrated that loss of Akt negatively influenced Paneth cell survival and Lgr5^+^ stem cells and TACs survival and proliferation (Fig. 1j). Therefore, we sought to determine whether PI3K/Akt inhibition would have a similar effect. Notably, H&E staining of small intestine sections revealed a significant reduction in villus length, and like the genetic deletion the intestinal architecture was disrupted following treatment with the inhibitors (Fig. 3i). PI3K or Akt inhibitors compromised the survival of ISCs and Paneth cells as well as TACs and all differentiated cell types (Fig. 3i). Structural damage to intestinal architecture and reduction in proliferation induced by PI3K/Akt inhibition were further confirmed in vitro. Intestinal crypts were exposed in culture to three different doses of the PI3Ki (BKM120, BYL-719, and GDC-0941) or Akt inhibitors (MK-2206, AZD5363, and GDC-0068) (Fig. S3). PI3K inhibition significantly decreased the organoid area compared to the control group (Fig. S3a). Furthermore, the reduction in the organoid area was accompanied by induction of cell death detected by PI staining (Fig. S3b). Similarly, treating already established organoids, 4 days after initiating the culture, with the same inhibitors had a negative effect on the organoid structure and viability (Fig. S3c). In parallel, Akt inhibition had a negative impact on organoid growth and integrity (Fig. S3d). In agreement with this, inhibition of PI3K or Akt markedly decreased proliferation and enhanced apoptotic cell death (Fig. S3e, f). Taken together, our results demonstrate that inhibition of the PI3K/Akt pathway phenocopies the deleterious effect exerted by genetic loss of Akt on crypt homeostasis.

### Intestinal *FoxO1* deletion reverses intestinal damage and mortality induced by Akt1 and Akt2 deletion

**Fig. 4:**
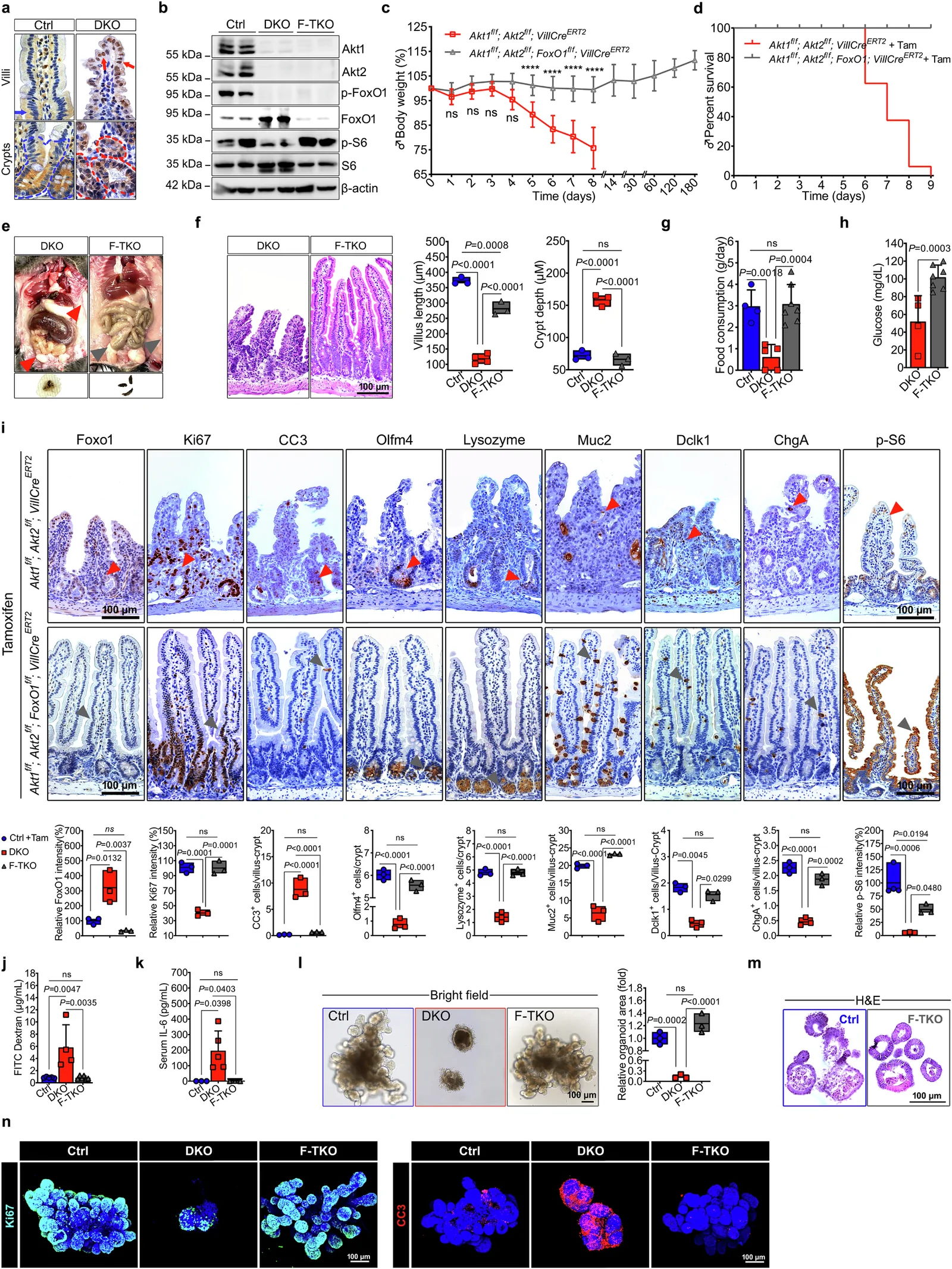
Intestinal *FoxO1* deletion restores intestinal epithelial integrity and tissue homeostasis in Akt intestinal deficient mice. **a** Representative IHC staining demonstrating FoxO1 nuclear localization in the villi and crypts of *Akt1/Akt2*-deleted mice. (*n* = 3). **b** Immunoblot confirming deletion of all floxed genes and activation of mTORC1 in the absence of Akt after FoxO1 removal. (*n* = 3). **c** Body weight changes up to 6 months post-Tam. (*n* = 18, 24). Mean ± SD. **d** Kaplan-Meier plot. (*n* = 16). **e** Representative images of internal organs indicating fat consumption (colored arrowheads) and diarrhea in DKO but not F-TKO mice. (*n* = 3). **f** H&E staining followed by villus length and crypt depth quantification. (Vill and crypts measurement for DKO are from Fig. 1h). (*n* = 3, 3-4, 3). One-way ANOVA (Tukey’s post-hoc test). **g** Food consumption. Values for Ctrl and DKO are from Fig. 1f oil and Tam. (*n* = 4, 5, 7). Mean ± SD. One-way ANOVA (Tukey’s post-hoc test). **h** Blood glucose. (*n* = 4, 7). Mean ± SD. Unpaired two-tailed t-test. **i** IHC analysis showing a normal differentiation pattern of intestinal epithelium as well as mTORC1 activation following FoxO1 deletion in F-TKO. Quantification of staining (lower). (*n* = 3). Quantification of Ctrl + Tam and DKO mice was adopted from (Fig. 1j). **j** Permeability assay demonstrates that additional deletion of *FoxO1* maintains intestinal integrity. (*n* = 5, 4, 6), technical duplicate. **k** Serum IL-6 levels. (*n* = 3, 5, 3), technical duplicate. (j,k) Ctrl = *Akt1*^*f/f*^; *Akt2*^*f/f*^; *VillCre*^*ERT2*^ + Oil. Mean ± SD. One-way ANOVA (Tukey’s post-hoc test). **l** Representative bright field images alongside quantification of organoid area. (*n* = 3, 5 organoids per group). Floating bars (**f**, **i**, **l**) show minimum, mean, and maximum. **m** Representative images of OCT-embedded and H&E-stained intestinal organoids demonstrating similarly normal structure of organoids from F-TKO compared to Ctrl. (*n* = 3). **n** Representative images of IF staining emphasizing the differences in proliferation and apoptosis between the three groups. (*n* = 3). Ctrl (**l**, **m**, **n**): *Akt1*^*f/f*^; *Akt2*^*f/f*^; *VillCre*^*ERT2*^ + EtOH. Source data are provided as a Source Data file.

Subsequently, we sought to uncover the mechanism underlying intestinal damage mediated by Akt deficiency, with the aim of identifying strategies to rescue this phenotype. Interestingly, KEGG pathway analysis, shown in Fig. S2i, demonstrated that the FOXO signaling pathway was among the most highly activated pathways following Akt deletion. Again, two more independent databases suggested FOXO (FoxO1) and P65 (RELA) among the most highly enriched transcription factors (Supplementary Data 3). We first investigated whether FOXO, which is an evolutionarily conserved target of Akt, is involved. FoxO1 is widely expressed in the gut of mice and humans^54,55^. Indeed, inactivation of the PI3K/Akt pathway completely blocked FoxO1 inhibitory phosphorylation (Ser256) (Fig. S4a) and markedly increased P65 phosphorylation (ser536) (Fig. 3e). Similarly, genetic ablation of Akt markedly diminished FoxO1 phosphorylation (Fig. 4). We also found that following Akt intestinal deletion, FoxO1 was mainly nuclear, contrasting with the cytosolic localization observed in the control group (Fig. 4a). Hence, we examined whether *FoxO1* intestinal ablation could reverse the intestinal damage mediated by *Akt* deletion and restore tissue homeostasis. We generated two mouse models of intestine-specific Akt and FoxO1 deficiency: *Akt1*^*f/f*^*; Akt2*^*f/f*^*; FoxO1*^*f/f*^*; VillCre*^*ERT2*^ (Fig. 4), and *Akt1*^*f/f*^*; Akt2*^*-/-*^*; FoxO1*^*f/f*^*; VillCre*^*ERT2*^ (Fig. S4), both referred to here as F-TKO. These mice were injected with Tam as previously described and were compared to Akt*1*^*f/f*^*; Akt2*^*f/f*^*; VillCre*^*ERT2*^ and *Akt1*^*f/f*^*; Akt2*^*-/-*^*; VillCre*^*ERT2*^ mice, both referred to here as DKO. Deletion of *Akt1/Akt2* and *FoxO1* following Tam administration was confirmed by PCR (Fig. S4b), and protein samples isolated from F-TKO small intestine indicated a complete loss of Akt and FoxO1 (Fig. 4b). Intriguingly, F-TKO mice exhibited slight body weight loss and appeared relatively normal during the time of injection, in comparison to DKO, which had a significant body weight reduction (Figs. 4c and S4c). Of note, we closely monitored the F-TKO mice for more than 6 months post-Tam injection, and consistently detected no change in their viability, while the DKO mice consistently showed 100% lethality (Figs. 4d and S4d). Moreover, no signs of diarrhea were seen or a reduction in gWAT was detected (Figs. 4e and S4e). Most importantly, the structural and functional impairment observed in intestinal Akt-deficient mice was completely reversed with additional deletion of FoxO1 (Figs. 4f and S4f). Although DKO mice exhibited destroyed villi filled with apoptotic cells and deeper crypts, F-TKO mice maintained normal epithelium without histological abnormalities (Figs. 4f and S4f). Notably, the length of the small and large intestines after *FoxO1* deletion were also back to normal parameters regardless of the loss of Akt (Fig. S4g).

We next investigated whether deletion of *FoxO1* could restore the ability of mice with *Akt1* and *Akt2* intestinal deletion to utilize food and maintain glucose homeostasis and found that food consumption (Fig. 4g) and glucose levels (Fig. 4h) were restored to the same levels as seen in control groups. Also, most of the crypts had an average of 5 stem cells intermingled by 5 Paneth cells, suggesting preserved stem cell survival and differentiation despite Akt deficiency (Figs. 4i and S4h). Further analysis of the IHC staining of Ki67^+^, Muc2^+^, Dclk1^+^, ChgA^+^ cells confirmed a normalization of proliferation and differentiation capacities in Akt null tissues following *FoxO1* deletion. Critically, deletion of *FoxO1*, which was also confirmed by IHC staining in F-TKO mice, protected from apoptotic cell death caused by Akt ablation and FoxO1 nuclear localization (Figs. 4i and S4h). These results demonstrate a critical role for FoxO1 activation in inducing intestinal damage and strongly suggest that targeting FoxO1 protects from tissue injury and inflammation induced by Akt deletion. Consistent with these observations, intestinal permeability was comparable between F-TKO and the control group (Figs. 4j and S4i). This was accompanied by normalization of IL-6 levels and prevention of immune cell infiltration (Figs. 4k and S4j). These findings are consistent with the ability of the mice to utilize food, maintain tissue homeostasis, and body weight. Furthermore, our analysis of old F-TKO mice revealed no detectable alteration in physiological parameters, histological characteristics, or overall tissue architecture and viability compared to control mice. Of note, gWAT following the deletion of *FoxO1* was only slightly affected, however, upon recovery, the adipocyte area was completely restored in comparison to a nearly complete loss of lipid contents in DKO tissues (Fig. S4k). Finally, crypts lacking Akt could form organoids and maintain intestinal organoid architecture, when FoxO1 was concurrently deleted (Fig. 4l, m). Also, proliferation and cell death rates in F-TKO and control organoids were comparable (Fig. 4n). Intriguingly, the deletion of Akt increased FoxO1 levels whereas mTORC1 activity, as measured by p-S6, was significantly reduced (Figs. 4b and S4l). Conversely, FoxO1 deletion elicited activation of mTORC1 even in the absence of Akt as shown by immunoblotting (Figs. 4b and S4l) and IHC (Figs. 4i and S4h). Altogether, loss of Akt in the intestinal epithelium enhances FoxO1 nuclear retention and activity, potentially leading to epithelial cell death and intestinal tissue damage. Indeed, deletion of *FoxO1* is sufficient to counteract the intestinal damage and mortality induced by Akt deficiency. Importantly, intestinal deletion of *FoxO1* alone elevated mTORC1 activity (Fig. S5a, b), consistent with our results from the F-TKO model and previous findings^56^. However, *FoxO1* deletion had no detectable effect on intestinal homeostasis (Fig. S5c), or mucus-producing goblet cells (Fig. S5d), contrary to a previous report^54^.

**Fig. 5:**
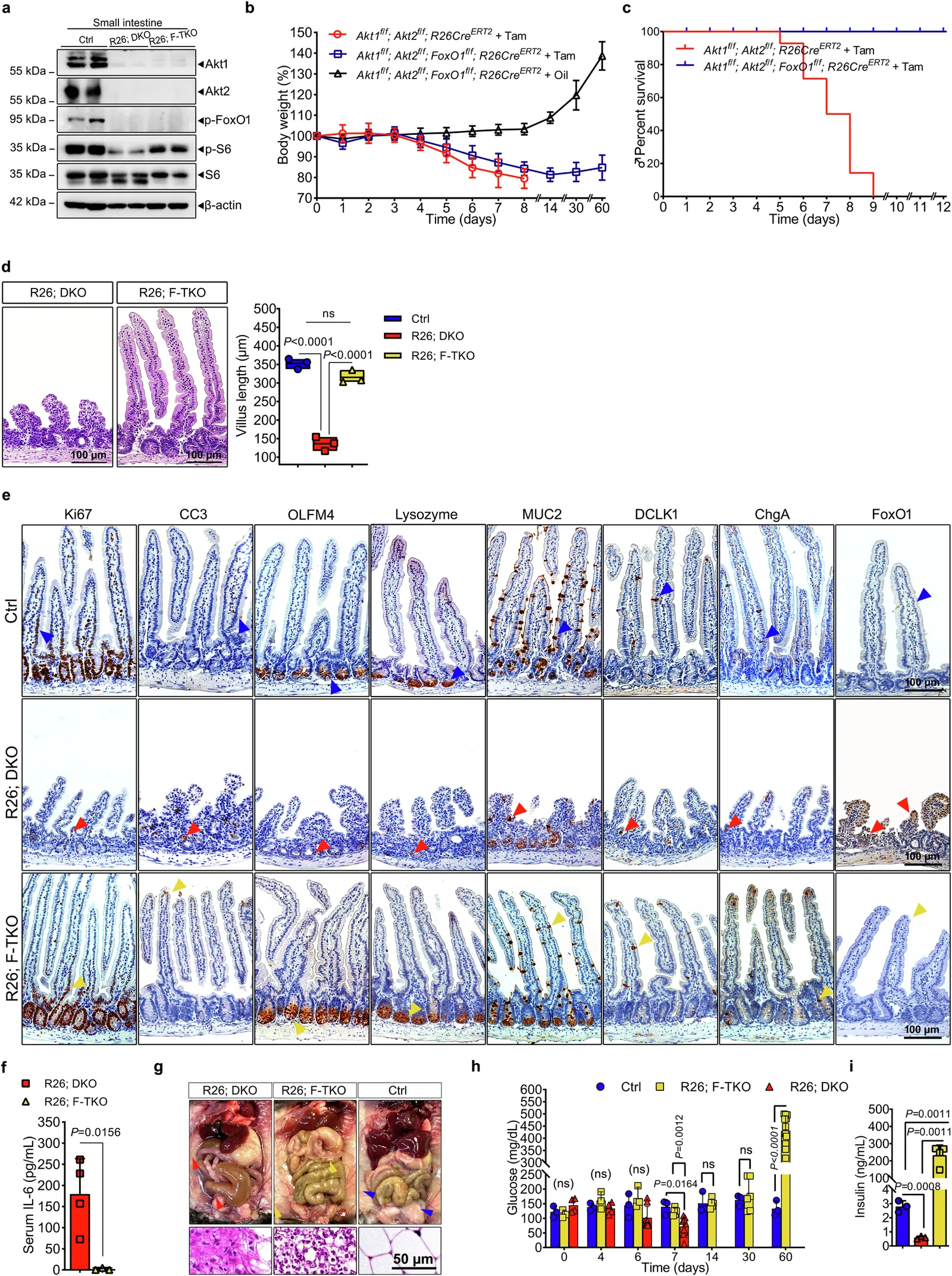
Systemic FoxO1 deletion restores viability of mice with systemic Akt1 and Akt2 deletion. **a** Small intestine lysates isolated from Ctrl (*Akt1*^*f/f*^*; Akt2*^*f/f*^*; FoxO1*^*f/f*^*; VillCre*^*ERT2*^ + Oil), *Akt*1 and *Akt2* whole-body knockout (R26; DKO), and whole-body *Akt1*, *Akt2*, and *FoxO1* deleted mice (R26; F-TKO) were immunoblotted with Akt1, Akt2, p-FoxO1, p-S6, S6 total, and Actin antibodies. (*n* = 3). **b** The change in body weight between the indicated groups over 2 months. (*n* = 12, 23, 10). Mean ± SD. Statistical analysis was performed using a mixed-effects model (Tukey’s post-hoc multiple comparisons test). **c** Kaplan–Meier plot demonstrates the survival of R26; F-TKO mice despite progressive reduction in body weight. (*n* = 14 mice per group). **d** H&E staining images (Left), and the quantification of the villus length (Right). (*n* = 3). Floating bars show the minimum to maximum range with the internal horizontal line representing the mean. **e** Representative IHC from staining the small intestine of Ctrl, R26; DKO, and R26; F-TKO. Colored arrowheads to highlight the differences in the indicated staining between the three groups. (*n* = 3). **f** IL-6 levels measured in the serum of the indicated groups. (*n* = 4, 3), technical duplicate. Mean ± SD. Unpaired two-tailed t-test. **g** Representative images depicting consumption of body fat in both R26; DKO and R26; F-TKO compared to Ctrl (upper), and H&E stained gWAT demonstrating the reduction of adipocyte area in both experimental groups compared to Ctrl (lower). (*n* = 3). **h** Blood glucose levels showing induction of hypoglycemia in R26; DKO (7 days post Tam injection) and hyperglycemia in R26; F-TKO (60 days post injection) compared to normal levels in the Ctrl group. (exact *n* values for each group are provided in the Source Data file). Mean ± SD. Unpaired, two-tailed Welch’s t-test. **i** Insulin levels measured in the serum of R26; DKO (1 week post injection), R26; F-TKO, and Ctrl mice (2–3 months post injection). (*n* = 3, 3, 4), technical duplicate. Mean ± SD. Unpaired, two-tailed Welch’s t-test. Source data are provided as a Source Data file.

Next, we sought to investigate whether systemic *FoxO1* deletion could rescue the mortality caused by Akt1 and Akt2 systemic deletion. For this purpose, we generated *Akt1*^*f/f*^; *Akt2*^*f/f*^; *FoxO1*^*f/f*^; *R26Cre*^*ERT2*^ mouse strain, referred to here as (R26; F-TKO). We confirmed the deletion of the floxed genes by immunoblotting (Fig. 5a). Triple systemic deletion of *Akt1*, *Akt2* and *FoxO1*, initially reduced body weight to a similar extent as that observed after *Akt1* and *Akt2* deletion only (Fig. 5b). Surprisingly, however, unlike *Akt1*^*f/f*^; *Akt2*^*f/f*^; *R26Cre*^*ERT2*^ (R26; DKO) mice that succumbed to rapid mortality after *Akt1* and *Akt2* deletion owing to severe intestinal damage, R26; F-TKO mice remained viable (Fig. 5c) and did not display the intestinal phenotype of the R26; DKO model (Fig. 5d, e). Moreover, IL-6 levels measured in these mice were indistinguishable from those of the control group (Fig. 5f). Thus, systemic deletion of FoxO1 is sufficient to protect from intestinal injury and rapid mortality elicited by systemic Akt1 and Akt2 deletion. Importantly, the R26; F-TKO mice survived the deletion for at least 6 months after Tam administration despite the significant body weight loss. The mice stayed lean and lost body fat (Fig. 5b, g). Notably, these mice became highly hyperglycemic and hyperinsulinemic 2 months after the deletion (Fig. 5h, i), which is consistent with the role of Akt1 and Akt2 in glucose homeostasis, adipocyte differentiation, and the maintenance of adipose tissues^6,57–60^. Interestingly, after *Akt1* and *Akt2* deletion, FoxO1 was exclusively localized to the nucleus, not only in the intestine (Fig. 5e), but also in the lung, kidney, and liver (Fig. S6a). As shown previously in intestine-specific deletion models (Figs. 4 and S4), when *FoxO1* was systemically deleted in Akt-deficient mice, mTORC1 activity was markedly elevated (Figs. 5a and S6b). Consistently, we obtained the same results not only from the intestine but also from lung and liver (Fig. S6b). Indeed, this is consistent with feedback loops exerted by activated FOXO to inhibit mTORC1 activity^56,61^. Finally, systemic deletion of Akt1 and Akt2 induced cell death and tissue damage in other tissues including liver and kidney, which was also ameliorated by concurrent *FoxO1* systemic deletion (Fig. S6c, d). Together, these results again confirm a critical role for Akt in protecting against intestinal tissue damage via inactivation of FoxO1 and suggest the same effect in different tissues.

### Hyperactivation of intestinal mTORC1 protects against intestinal injury and mortality induced by intestinal *Akt* deletion

**Fig. 6:**
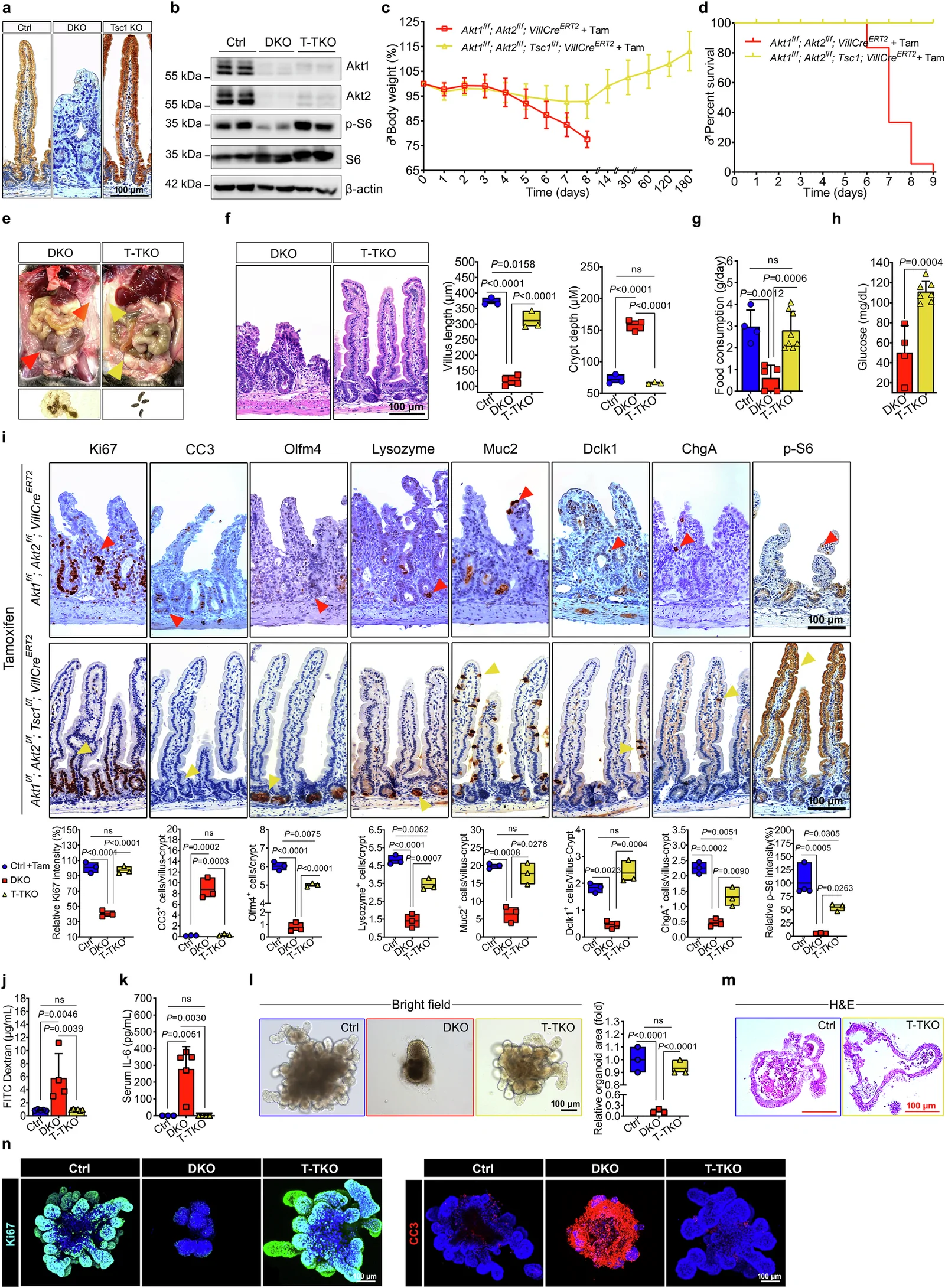
Restoration of intestinal epithelium homeostasis and viability in mice lacking intestinal Akt is achieved via enhanced mTORC1 activity. **a** IHC analysis demonstrates mTORC1 inhibition in DKO compared to Ctrl and confirms hyperactivation of mTORC1 after *Tsc1* intestinal deletion. (*n* = 3). **b** Representative immunoblots confirm loss of Akt and induction of S6 phosphorylation post Tam treatment in T-TKO compared to control. (*n* = 3). **c** Change in body weight following Tam injection. (*n* = 15, 27). Mean ± SD. **d** Kaplan-Meier plot. (*n* = 18). **e** Representative abdominal images demonstrating exhaustion of adipose tissues (red arrowheads) and diarrhea in DKO (left) versus maintenance of adipose tissues (yellow arrowheads) and normal stool in T-TKO. (*n* = 3). **f** H&E staining depicting small intestine structure (left). Quantification of villus length and crypt depth (Right). (*n* = 3, 4, 3). Crypts and villi measurements for Ctrl and DKO are from Fig. 4f Ctrl and DKO. **g** Food intake. (*n* = 4, 5, 8). Values for Ctrl and DKO are from Fig. 1f oil and Tam. **h** Blood glucose levels showing maintenance of glucose homeostasis in T-TKO (*n* = 4, 7). Mean ± SD. Unpaired two-tailed t-test. **i** Representative images of IHC staining for various intestinal markers (upper) and statistical analysis (lower). (*n* = 3). **j** Intestinal permeability (*n* = 4, 4, 5). Mean ± SD. One-way ANOVA (Tukey’s post-hoc test). **k** Serum levels of IL-6 confirm a protective role of mTORC1 activation against intestinal damage-induced systemic inflammation. (*n* = 3, 5, 4). **g**, **j**, **k** Mean ± SD. One-way ANOVA (Tukey’s post-hoc test). **l** (Left) Representative bright-field images showing organoid formation (passage 2) in T-TKO vs. complete loss of regeneration in DKO crypts. (Right) Statistical analysis of organoid area. (*n* = 3 independent experiments). **f**, **i**, **l** Floating bars show minimum, mean, and maximum range. **m** Representative H&E staining of Ctrl versus T-TKO OCT embedded organoids depicting normalization of organoid architecture following *Tsc1* deletion despite the loss of Akt1/Akt2. (*n* = 3). **n** Representative IF of Ki67 CC3 staining. (*n* = 3 independent experiments). **j**–**n**) Ctrl = *Akt1*^*f/f*^; *Akt2*^*f/f*^; *VillCre*^*ERT2*^ + EtOH.

mTORC1 is a critical evolutionarily conserved downstream effector of the PI3K/Akt pathway. As was shown previously, at least in vitro, the hyperactivation of mTORC1 inhibits Akt activity through negative feedback loops^62–65^, and once mTORC1 is hyperactivated, Akt is no longer required for cell proliferation^66^. In addition, the deletion of *FoxO1*, which rescued the lethality elicited by *Akt1* and *Akt2* deletion, increased mTORC1 activity (Figs. 4b, i; S4h, l; 5a, and S6b). Therefore, we asked whether tissue damage mediated by Akt loss could be ameliorated by hyperactivation of mTORC1. As depicted in (Fig. S7a), Akt phosphorylates Tsc2, thereby inhibiting the activity of Tsc1/Tsc2 heterodimer, which is a negative regulator of mTORC1 that inhibits its upstream positive regulator, Rheb, and thereby deletion of either *Tsc1* or *Tsc2* leads to hyperactivation of mTORC1^67–71^. Thus, we generated mouse models in which *Akt1* and *Akt2* are deleted together with Tsc1 inducible knockout: (*Akt1*^*f/f*^*; Akt2*^*f/f*^*; Tsc1*^*f/f*^*; VillCre*^*ERT2*^ (Fig. 6)) and (*Akt1*^*f/f*^*; Akt2*^*-/-*^*; Tsc1*^*f/f*^*; VillCre*^*ERT2*^ (Fig. S7)) both referred to as T-TKO. As before, the mice were subjected to Tam administration to delete the floxed genes. As expected, *Tsc1* deletion only enhanced mTORC1 activity in comparison to DKO and control mice (Fig. 6a). The deficiency of Akt1, Akt2, and Tsc1 was confirmed by immunoblotting and PCR analysis (Figs. 6b, S7b, l). In contrast to the DKO mice, T-TKO mice maintained their body weight, except for a slight reduction during the time of treatment and recovery (Figs. 6c and S7c) and remained viable (Figs. 6b and S7d). T-TKO mice appeared overtly normal and exhibited no body fat loss or diarrhea (Figs. 6e and S7e). Furthermore, intestinal villi and crypt depth (Figs. 6f and S7f), food consumption (Fig. 6g), small and large intestine lengths (Fig. S7g), and glucose levels (Fig. 6h) were normalized in T-TKO mice. Finally, mTORC1 activity in T-TKO mice was further confirmed by IHC staining (Figs. 6i and S7h). Consistently, proliferation and cell death rates were back to normal in T-TKO mice in comparison with DKO and control mice (Figs. 6i and S7h). Moreover, a complete restoration of epithelium regeneration capacity, which was evident by the significant increase in ISCs (Olfm4^+^), Paneth cells (Lysozyme^+^), proliferative cells (Ki67^+^), goblet cells (Muc2^+^), tuft cells (Dclk1^+^) and enteroendocrine cells (ChgA^+^), was observed in T-TKO compared to DKO mice (Figs. 6i and S7h). It should be noted, however, that although not significant, there was a slight reduction in the recovery of Paneth cells after *Tsc1* deletion, which is consistent with previous results showing that hyperactivation of mTORC1 caused by Tsc1 deletion decreases Paneth cells^72^. Intestinal integrity, evaluated by permeability assay (Figs. 6j and S7i) and IHC staining for myeloid cell marker MRP8 (S100A8) (Fig. S7j), was preserved within the physiological ranges. Consistently, no induction of IL-6 was detected in T-TKO mice (Fig. 6k). Restoration of body weight in T-TKO was associated with the recovery of adipose tissues (Fig. S7k). Consistent with normal intestinal regeneration in vivo, we were able to grow organoids from T-TKO crypts, without any significant differences between these organoids and those collected from the control group (Fig. 6l–n).

Interestingly, in the two mouse models of T-TKO, FoxO1 is retained in the nucleus (Fig. S8a). This is consistent with Akt’s role in phosphorylating FoxO1, leading to its nuclear exclusion. However, since these mice survived Akt deletion and FoxO1 nuclear localization, it implies that hyperactivation of mTORC1 overcomes, at least to some extent, FoxO1 activity. Indeed, RNA-seq analysis showed that many of the genes significantly affected by Akt deletion were restored after either *FoxO1* or *Tsc1* deletion (Fig. S8b, and supplementary Data 5). Specifically, analyzing FOXO target genes^73^, showed that expression of most FOXO target genes in the intestine of DKO mice was reversed in T-TKO mice (Fig. S8c and Supplementary Data 5), suggesting that mTORC1 hyperactivation overrides FoxO1 activity despite its nuclear localization by an unknown mechanism. We concluded that intestinal hyperactivation of mTORC1 by the deletion of Tsc1 is sufficient to completely restore intestinal integrity and viability in mice lacking intestinal Akt1 and Akt2 despite FoxO1 nuclear localization. Finally, the expression levels of intestinal homeostasis genes (epithelial cell markers and NOTCH and WNT-related genes) were normalized in F-TKO and T-TKO tissues. Similarly, the elevated NF-κB target genes in the DKO mice were diminished in either the F-TKO or T-TKO mice (Fig. S8d, e and Supplementary Data 6).

### Inactivation of mTORC1 by intestinal Raptor deletion recapitulates Akt1 and Akt2 intestinal deletion

**Fig. 7:**
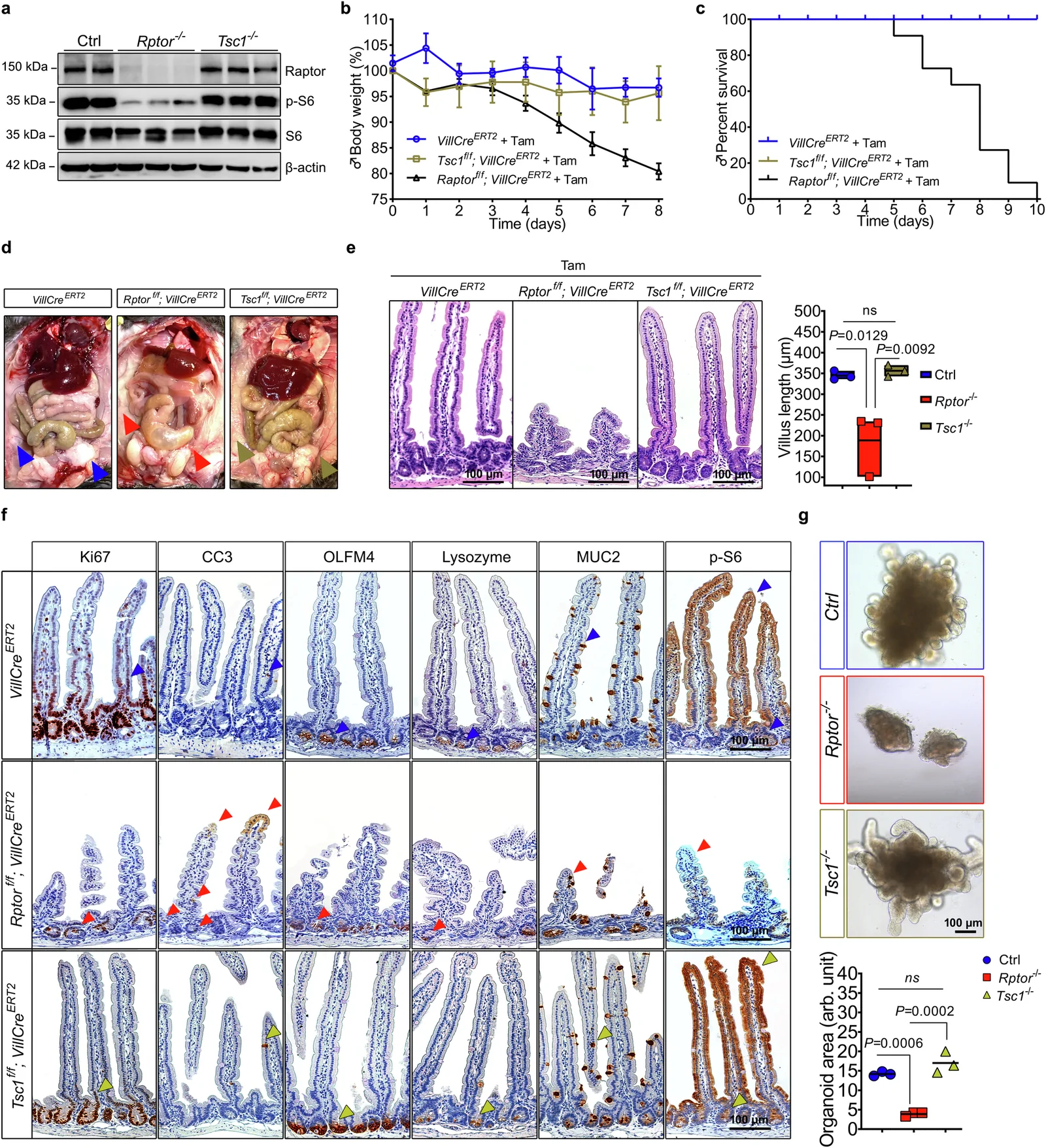
Intestinal mTORC1 is essential for intestinal homeostasis and mouse viability. **a** Immunoblot confirming the deletion of Raptor in *Rptor*^*f/f*^*; VillCre*^*ERT2*^. Reduction in p-S6 levels further confirms Raptor deficiency and mTORC1 inhibition, while elevated p-S6 confirms Tsc1 deletion and mTORC1 activation. (*n* = 3). **b** Differences in body weight between the indicated genotypes following Tam injection, shown as percent of the initial weight, highlight the importance of intestinal mTORC1 for maintaining body weight (*n* = 10, 21, 11). Mean ± SD. Statistical analysis was performed using a mixed-effects model (Tukey’s post-hoc multiple comparisons test). **c** Kaplan–Meier survival curve highlighting the essentiality of mTORC1 for viability. (*n* = 11 mice per group). **d** Representative images of internal organs from control, Raptor deficient, and Tsc1-deficient mice demonstrating adipose tissue depletion and diarrhea following intestine-specific raptor deletion compared with *Tsc1* KO and control mice. (*n* = 3). **e** Representative H&E-stained small intestine sections demonstrating that Raptor deletion mimics the effect of Akt deficiency in small intestine and leads to extensive destruction of the mucosal layer (Left). Quantification of villus length (Right). (*n* = 3). Floating bars show the minimum to maximum range with the internal horizontal line representing the mean. **f** IHC staining emphasizes the disruption of intestinal epithelial stem cell survival, proliferation, and differentiation. (*n* = 4 mice per group). **g** Representative images show deficiency in the ability of *Rptor*-deleted crypts to form organoids in comparison to Ctrl (*VillCre*^*ERT2*^ + Tam) and *Tsc1*-depleted crypts (upper). Statistical analysis of organoid area (lower). (*n* = 3, 10 organoids per group). Data are presented as a scatter dot plot indicating sample mean ± SD.

The results showing that hyperactivation of mTORC1 overcomes the consequences of intestinal Akt deficiency, suggest that mTORC1, downstream of Akt, is critical for intestinal integrity. However, previous results showed that intestinal removal of Raptor, which determines mTORC1 activity, by itself, did not elicit intestinal damage or lethality. Rather, intestinal Raptor deletion or pharmacological inhibition of mTORC1 was shown to impair crypt regeneration and disrupt intestinal homeostasis only following injury, and to limit tumorigenesis. Moreover, mTORC1 inhibition exacerbates mucosal atrophy following irradiation in vivo and significantly diminishes crypts’ ability to form organoids ex vivo^19,74,75^. Given that mTORC1 hyperactivation overcomes Akt-deficiency-induced intestinal damage, these previous studies are surprising and could be due to incomplete deletion of Raptor. To address this, we generated (*Raptor*^*f/f*^; *VillCre*^*ERT2*^) mice, in which a complete deletion of *Raptor* was achieved by administration of Tam for 7 consecutive days, and marked reduction of mTORC1 activity was detected (Fig. 7a). Following Raptor ablation, mice exhibited the same phenotype we observed after intestinal *Akt1* and *Akt2* deletion, characterized by swift body weight loss, consumption of body fat, and lethality (Fig. 7b–d). Likewise, mice lacking Raptor experienced diarrhea and the cecum was filled with loose or watery stool (Fig.7d). Raptor-deficient mice had shorter villi (Fig. 7e), increased cell death, impaired cell proliferation, and compromised cellular differentiation and intestinal epithelial integrity (Fig. 7f). Small intestine tissues with intestinal Raptor deletion exhibited a great reduction in p-S6 staining compared to control, further confirming efficient mTORC1 inactivation (Fig. 7f). Conversely, intestinal Tsc1 deficiency as expected resulted in a significant elevation in p-S6 staining compared to Raptor-deficient tissues (Fig. 7f). In parallel to the in vivo studies, we isolated crypts from all these mice and incubated them in culture with 4-OHT to induce the deletion of the floxed genes. Consistently, loss of Raptor prohibited organoid formation to the same extent as seen in crypts lacking both Akt1 and Akt2 isoforms. Taken together, these results show that mTORC1 is the most critical downstream effector of Akt that is not only required, but also sufficient, for intestinal homeostasis and survival.

## Discussion

Our results revealed that the mortality induced by Akt1 and Akt2 systemic deletion is mediated by intestinal damage. We showed that, like the systemic deletion, intestinal Akt1 and Akt2 deletion is sufficient to induce rapid mortality due to intestinal damage and inflammation. Extensive histological analysis, including IHC staining, revealed severe damage and alteration in the structural architecture of the small intestine and colon. Specifically, the villi in the small intestine were blunted and exposed, and significant abnormalities in the large intestine architecture were also observed. For instance, invaginations of crypts were substantially diminished, and the surface of the epithelium was markedly flattened. Our results clearly showed that Akt is essential for Lgr5^+^ stem cell survival and proliferation and for Paneth cell viability. Additionally, loss of Akt dampened TACs' proliferation and migration capacity; as determined by the reduced abundance and random distribution of Ki67^+^ cells within the villus-crypt axis. Consequently, the number of differentiated cells significantly decreased. Notably, differentiated cells in the epithelium not only showed a reduction in their abundance, but also alteration in their size, content, and localization. The effect of Akt deletion on the intestine explains the notable reduction in food absorption and nutrient uptake, followed by induced lipolysis and hypoglycemia. The increased intestinal permeability can be explained by the increased cell death as it was shown that apoptotic cells within epithelial mucosa can create gaps within the epithelial barrier leading to increased intestinal permeability. Increased permeability could then allow gut-derived bacteria to invade the circulation and endotoxins to translocate into the bloodstream leading to chronic and systemic inflammation^76–82^. Consistent with the results in vivo, crypts lacking Akt failed to generate organoids in culture.

To further investigate the requirement of Akt for cell-intrinsic stem cell survival, rapid self-renewal, and the high turnover rate of the intestinal epithelium, we generated a mouse model with inducible deletion of Akt, exclusively in the Lgr5^+^ stem cells. Surprisingly, our results revealed no significant differences between Tam- and oil-injected mice. Namely, we did not observe any noticeable effect on body weight or mouse survival. Indeed, these discrepancies between the Villin-driven and the Lgr5-driven Cre systems were previously reported^48–50^. First, it was shown that upon depletion of the Lgr5^+^ stem cell pool, other epithelial cell types closely resembling Lgr5^+^ cells or markedly distinct may serve as reservoirs. These cells immediately compensate for the loss of stem cells, rendering Lgr5^+^ stem cells dispensable^14,46,51,52,83–94^. Indeed, we noticed a significant increase in Bmi1 and Lgr4^46,95^ mRNA levels following intestinal *Akt* deletion (Fig. S2g). Conversely, it was demonstrated that Lgr5^+^ cells are essential even during regular epithelial replenishment and that small intestines lacking this population of cells exhibit compromised integrity^29,96^. However, it was also suggested that the negligible effect associated with Lgr5^+^ cells ablation is attributed in part to the mosaic expression pattern of *Lgr5* and could be circumvented by using different systems that may provide a robust Cre recombination^29,97–99^. Finally, these discrepancies come in agreement with our findings and suggest that reliance on the *Lgr5-EGFP-Cre*^*ERT2*^ model in editing Lgr5^+^ cells, specifically in vivo, remains a matter of discussion and warrants further clarification. On the other hand, we found that Akt deletion specifically in Lgr5^+^ stem cells precluded organoid formation and mediated cell death in culture. These results strongly suggest that Akt is required for Lgr5^+^ stem cell survival and that Lgr5^+^ stem cells are essential for organoid formation in vitro and probably crypt regeneration and tissue homeostasis in vivo. Indeed, if Lgr5^+^ stem cells were dispensable and could be compensated by other epithelial cell types following cell death or tissue damage, we would expect to see stem cell survival and organoid formation despite the absence of Lgr5^+^ stem cells.

Our genetic findings were confirmed pharmacologically by exposing the mice to pan-PI3K and pan-Akt inhibitors. Thus, these results could explain the occasional severe diarrhea observed in patients treated with these drugs and highlight the need for careful consideration of potential gut side effects when targeting the PI3K/Akt pathway in cancer patients^22,23^.

To identify the downstream effectors of Akt that are responsible for this phenotype, we analyzed our bulk mRNA-seq and found that the FOXO signaling pathway was one of the most highly active pathways, and FoxO1 was one of the top transcription factors activated in the small intestine after Akt deletion. Immunoblotting analysis demonstrated a significant reduction in FoxO1 phosphorylation, IHC staining demonstrated nuclear localization, and RNA-seq analysis confirmed induction of many FOXO target genes. Therefore, we focused on the FoxO1 transcription factor and mTORC1, which are also the most evolutionarily conserved downstream effectors of Akt. For these reasons, we deleted FoxO1 together with Akt1 and Akt2 and showed that this was sufficient to overcome the lethality induced by Akt deletion. In the absence of FoxO1, Akt1 and Akt2 deletion did not exert intestinal damage, did not affect food intake, and had no impact on intestinal permeability and inflammation as measured by FITC-dextran and IL-6 levels, respectively. Interestingly, mTORC1 activity, which is diminished in the absence of Akt1 and Akt2, is re-elevated when FoxO1 is also deleted, likely because of negative feedback loops exerted by FOXO on mTORC1 activity^56,61^. However, we cannot exclude other mechanisms by which FoxO1 deletion activates mTORC1 in the absence of Akt. These potential mechanisms likely exert their effects downstream of the negative regulator of mTORC1, the TSC1/TSC2 heterodimer, which is activated by Akt deficiency. These results raised the possibility that the reactivation of mTORC1 by FoxO1 deletion plays a role in overcoming intestinal damage and mortality. Consistently, hyperactivation of mTORC1 by intestinal *Tsc1* deletion was also able to overcome the intestinal damage and mortality induced by Akt deletion. Thus, it is possible that the diminishment of mTORC1 activity by Akt deletion is a major reason for the intestinal damage and lethality. We obtained support for this possibility by inhibiting intestinal mTORC1 via Raptor deletion and found that mTORC1 inhibition phenocopied Akt deletion, leading to intestinal damage and mortality. Notably, it was previously reported that intestinal deletion of FoxO1 impairs intestinal homeostasis and mucus production^54^. However, we did not observe this phenotype after inducible intestinal deletion of FoxO1, although mTORC1 was activated. The discrepancy could be because we used inducible intestinal FoxO1 deletion in adult mice, whereas the other report used the constitutive Villin-Cre-mediated deletion. Furthermore, a deletion of all FoxO isoforms in the intestine using Lgr5-Cre did not report this phenotype^20^.

Finally, we found that combined systemic deletion of *Akt1*, *Akt2*, and *FoxO1* did not induce intestinal damage or mortality. These results are unexpected because they imply that Akt1 and Akt2 are not required for adult viability if FoxO1 is also deleted. Germline deletion of *FoxO1* is embryonically lethal, and  to our knowledge, FoxO1 systemic deletion has not been tested in adult mice. Thus, at least in the absence of Akt1 and Akt2, systemic deletion of FoxO1 is tolerated. Of note, the deletion of *Akt1* and *Akt2* in the absence of FoxO1 elicits hyperglycemia and loss of body fat, which is consistent with the previously shown role of Akt2, in combination with Akt1, in regulating glucose homeostasis and adipose tissues^60^. Interestingly, intestinal deletion of Tsc1 in the absence of Akt1 and Akt2 retains nuclear FoxO1 localization. However, our results suggest that despite FoxO1 nuclear localization, hyperactivation of mTORC1 antagonizes its transcriptional activity, thus abrogating its ability to cause intestinal damage.

In summary, our findings demonstrate that Akt is required for intestinal homeostasis, which is mediated by the inhibition of FoxO1 and the activation of mTORC1. These results also provide in vivo evidence for the interplay between FoxO1 and mTORC1 and suggest a protective role for targeting FoxO1 in suppressing tissue damage and systemic inflammation.

## Methods

All antibodies, chemicals, reagents, and mouse strains used are listed in supplementary data 7. Dilutions of antibodies in supplementary data 8, and oligonucleotide sequences for genotyping primers are listed in supplementary data 9.

### Generation and genotyping of global and intestine-specific mutant mice

All animal protocols were approved by the Institutional Animal Care and Use Committee at the University of Illinois at Chicago. All mice were housed at controlled temperatures of 22 ± 1 °C in individually ventilated cages at a 12–h day/12–h night cycle with free access to rodent chow and water. C57BL/6J mice with conditional and global deletion of *Akt1* and *Akt2* were generated as previously described^8^. Transgenic mice carrying *Akt1*^*f/f*^ and *Akt2* ^*f/f*^ ^100,101^ were bred to *VillCre*^*ERT2*^ transgenic mice^26^ to generate intestinal epithelium-specific deletion of *Akt1* and *Akt2*. *Akt1*^*f/f*^ possesses *loxP* sites flanking exons 3–4, and *Akt2*^*f/f*^ possesses *loxP* sites flanking exons 4–5. Also, mice lacking Akt2 (*Akt2*^*-/-*^ germline deleted) and carrying *loxP*–flanked *Akt1* alleles (*Akt1*^*f/f*^) were bred to *Villin-Cre*^*ERT2*^ mice to generate Akt2 null strain with spatial and conditional deletion of Akt1 specifically in the intestine. After multiple crossings, (*Akt1*^*f/f*^; *Akt2*^*f/f*^; *VillCre*^*ERT2*^) and (*Akt1*^*f/f*^*; Akt2*^*-/-*^; *VillCre*^*ERT2*^) mice were stably generated and maintained in a pathogen-free condition. These mice were then crossed to (*FoxO1*^*f/f*^*; VillCre*^*ERT2*^ *or Tsc1*^*f/f*^*; VillCre*^*ERT2*^) to generate (*Akt1*^*f/f*^; *Akt2*^*f/f*^; *FoxO1*^*f/f*^*; VillCre*^*ERT2*^), (*Akt1*^*f/f*^; *Akt2*^*-/-*^; *FoxO1*^*f/f*^*; VillCre*^*ERT2*^), (*Akt1*^*f/f*^; *Akt2*^*f/f*^; *Tsc1*^*f/f*^*; VillCre*^*ERT2*^), and (*Akt1*^*f/f*^; *Akt2*^*-/-*^; *Tsc1*^*f/f*^*; VillCre*^*ERT2*^), respectively. Moreover, we crossed (*Akt1*^*f/f*^; *Akt2*^*f/f*^; *FoxO1*^*f/f*^*; VillCre*^*ERT2*^) mice with (*Akt1*^*f/f*^; *Akt2*^*f/f*^; *R26Cre*^*ERT2*^) to generate a mouse model with conditional deletion of the three genes in the whole body upon tamoxifen-induced Cre recombinase. Additionally, *Akt1*^*f/f*^; *Akt2*^*f/f*^ and *Akt1*^*f/f*^*; Akt2*^*-/-*^ mice were bred to *Lgr5-EGFP-IRES-Cre*^*ERT2*^ transgenic mice, described here^9^, to generate intestinal stem cell-specific deletion of *Akt1* and *Akt2*. Finally, *FoxO1*^*f/f*^, *Tsc1*^*f/f*^*, and Rptor*^*f/f*^ mice were bred with the *VillCre*^*ERT2*^ expressing mice to generate (FoxO*1*^*f/f*^*; VillCre*^*ERT2*^*)*, (*Tsc1*^*f/f*^*; VillCre*^*ERT2*^) *and* (*Rptor*^*f/f*^*; VillCre*^*ERT2*^). Genotypes of the mice were confirmed by performing PCR on genomic DNA extracted from the tail as well as from internal organ homogenates.

### Establishment of organoid culture

As previously described^27,33^; the small intestine was isolated, the fat/mesentery was dissected away, and the lumen was washed with cold PBS (10X Phosphate-Buffered Saline, pH 7.4 ± 0.1, calcium and magnesium free #46-013-CM) and cut into 3 segments. Approximately 5–7 cm of the proximal segment (duodenum) was used for isolating crypts as follows. Duodenum segments were opened longitudinally, and mucus was washed with ice-cold sterile PBS using 1 mL tips, and villi were removed by scraping the tissue using a glass slide. Intestinal segments containing crypts were cut into 3–5 mm pieces, pipetted, and washed vigorously with PBS 10–20 times, then incubated with 2 mM EDTA on a shaker at 4 °C for 20 min. EDTA was removed, and crypts were washed 3 times with cold PBS. To liberate crypts, 20 mL PBS was added, and the tissue sample was hand-shaken for 15 s. To remove villous material and tissue fragments, the crypt-containing PBS solution was filtered through a 70 µm cell strainer (70 µm Cell Strainer, Falcon #352350). This step was repeated 2 more times to have 3 different mixes of (crypt/villi) fractions. All 3 fractions were pelleted at 290 × *g* for 5 min, washed in PBS, and pelleted again at 100 × *g* for 3 min and resuspended in 10 mL AdDMEM/F-12 medium (Advanced DMEM/F-12, Gibco #12-634-010). Crypts per mL were counted in all 3 fractions, and the one that had more crypts/single cells ratio was chosen for plating. Crypts were mixed with growth factor-reduced Matrigel (Matrigel Matrix for Organoid Culture, Phenol Red-free, LDEV-free, 10 mL, Corning #356255), seeded at 100 crypts/µL Matrigel in 24-well plates, kept in the incubator for 30–45 min to solidify, and organoid culture medium (IntestiCult™ Organoid Growth Medium (Mouse), STEMCELL Technologies #06005) was overlaid onto the Matrigel dome. Medium was changed every 3 days. All organoids were subjected at least once, to mechanical breakup, before they were used for any further experiments. Experiments were performed on passage 1 or higher, by adding either EtOH, 4-OHT, or PI3K and Akt inhibitors. Treatment started either on day 1, day 3, or day4, and organoids were collected on day 7 for analysis. Organoid images were taken using Celigo or bright-field microscopy.

### Tamoxifen and drug treatments in mice and organoids

For mice: Tamoxifen (Tam) (Sigma-Aldrich, #T5648-5G) was dissolved in corn oil (Sigma-Aldrich #C8267-500ML) at a dose of (2–3) mg/100 µL and kept overnight on the shaker at 37 °C to obtain a homogeneous solution. Control groups included: VillCre^ERT2^ mice expressing no floxed genes injected with Tam, floxed mice expressing VillCre^ERT2^ injected with oil, and Cre-free mice injected with Tam. Mice aged 8–12 weeks were injected intraperitoneally according to their body weight for 7 consecutive days. Sixty milligrams per kilogram of BKM120 (Buparlisib) MedChemExpress #944396-07-0 or 300 mg/kg of MK-2206 (MK-2206 dihydrochloride, MedChemExpress #1032350-13-2) were dissolved in vehicle (10% DMSO + 40% PEG300 + 5% Tween-80 + 45% Saline). Mice were injected for 2–3 weeks daily or every other day.

### Isolation of serum and plasma

After anesthesia, blood was collected from retro-orbital vein, centrifuged for 15 min at 2000 × *g* and serum was collected in clean tubes and kept at −80 °C until use. For plasma, blood was collected and diluted with an anticoagulant (15% v/v acid citrate dextrose solution), mixed thoroughly, centrifuged at 5000 rpm for 10 min. Plasma samples were collected in new tubes and kept on ice or at −80 °C for further experiments.

### Measurement of Glucose, insulin, and IL-6 levels

For glucose level, 1-2 µL blood samples were obtained from the tail vein nick without anesthesia. Glucometer (CONTOUR NEXT Blood Glucose Monitoring System, Ascensia) and strips (Contour Glucose Test Strips, Contour #00193-7090-21) were used to measure glucose daily at 5 pm. For IL-6 level, serum collected and frozen at −80 °C was obtained and IL-6 kits from Sigma-Aldrich were used according to the manufacturer’s instructions. Insulin levels were measured from the same serum samples using Rat/Mouse Insulin ELISA Kit (Millipore #EZRMI-13K) according to the manufacturer’s instructions.

### Western blot analysis

Small intestine tissues were washed in PBS, dissected, and homogenized in RIPA buffer supplemented with protease inhibitor (Thermo Scientific #A32953) and phosphatase inhibitor (Thermo Scientific #A32957), left on ice for 1 h during which cell lysates were vortexed 3 times 10 s each. Lysates were pelleted at 16,000 × *g* for 20 min, and the clear supernatants were moved to pre-labeled microcentrifuge tubes. Total protein was quantified using the Bradford method (Bio-Rad Laboratories #500-0006), boiled in 1X loading dye, resolved on a 10% SDS–PAGE, and transferred to PVDF membranes following standard methods. Membranes were incubated for 1 h at RT in 5% BSA/TBST for blocking, and overnight at 4 °C with the relevant primary antibodies. Membranes were then washed 3 times/5 min and incubated at RT for 45 min with HRP–conjugated secondary antibody. Protein expression was visualized using an enhanced chemiluminescence detection system (Azure Biosystems).

### Intestinal permeability assay

The intestinal permeability assay was performed according to the standard protocol to determine barrier function upon Akt intestinal deletion. Mice were fasted during the light cycle for 6 h and then orally administered FITC-dextran (Fluorescein isothiocyanate (FITC)-dextran, Sigma #60842-46-8). Blood was collected, and plasma was separated from mutant and WT mice before and 4 h post Dextran injection (60 mg/100 g body weight). FITC-dextran leaking into the blood serum was assessed by measuring the fluorescence intensity in plasma. Samples from before FITC-dextran administration were used for normalization.

### Tissue collection and histological analysis

Mice at the endpoint were euthanized using AnaSed (Xylazine Hydrochloride, Akorn Animal Health # NDC 59399-110-20) and Ketamine (Ketamine Hydrochloride, Hospira #0409-2051-05), and cervical dislocation was performed. The intestinal tract was removed and washed on ice with cold phosphate–buffered saline (PBS) several times in a Petri dish. The small intestine was divided into 3 segments: the second segment, jejunum, was used mainly for histological examination, while the first and third, duodenum and ileum, respectively, were used for other experiments. Jejunum tissues were opened longitudinally, washed gently, and fixed in a 10% neutral buffered formalin (Formalde-Fresh Solution, Thermo Fisher Scientific #SF93-4) overnight at 4 °C. The fixed tissue specimens were formed into Swiss rolls, dehydrated, embedded in paraffin, sectioned to 5 µm, and fixed on slides. Sections were deparaffinized in xylene, then rehydrated in ethanol, and stained with hematoxylin (Harris Hematoxylin, Sigma-Aldrich #HHS32-1L) for 30–60 s and washed in tap water 3 times, 1 min each. Excess stain was further removed by dipping the slides for 5 s in 1% acid alcohol (1% HCl in 70% ethanol), followed by incubation for 5 s in a bluing agent such as sodium bicarbonate (Thermo Fisher Scientific, #BP328-1). Slides were washed in a gentle stream of tap water for 1–3 min before staining with eosin. Hematoxylin-stained slides were counterstained with eosin (Eosin Y disodium salt, Sigma, #E-6003) for 10–30 s, dehydrated in 75%, 85%, and in 2 changes of 95% and 100% ethanol (Decon Labs, #2705) for 3 min each, and cleared through 2 changes of xylene for 3 min each. A xylene-based Permount Mounting Medium (Fisher Scientific #SP15) was applied immediately to the slides, which were cover slipped and let to dry. Finally, inverted microscope (Leica DMi8) was used to obtain images using 10X, 20X, and 60X (oil) objectives.

### OCT embedding and Cryo-sectioning

Duodenum sections were dissected, washed in cold PBS and fixed in 4% PFA (16% Formaldehyde (w/v), Methanol-free, Thermo Scientific #28908) for 1 h at RT. Fixed tissues were washed in 1X PBS (Fisher Scientific #BP661-50) 3 times, 5 min each, and preserved in 30% sucrose/PBS (Sigma-Aldrich, #F0127) overnight at 4 °C. Cryoprotected tissues were then quickly frozen in Tissue-Plus O.C.T. compound (Fisher Healthcare, #4585) using liquid nitrogen. Frozen blocks were sectioned at 5 μm thickness using a cryostat (Leica CM1950) and mounted on charged slides (Fisherbrand Superfrost Plus Microscope Slides, Fisher Scientific #1255015). Slides were stored at −80 °C until staining. Slides were removed from the freezer and left at RT to dry. To completely remove OCT, slides were rinsed in 1X PBS twice for 3 min, followed by 2 times washing in tap water. Slides were stained with Nile Red for lipid droplets and Hoechst 33342 (Hoechst Nucleic Acid Stains, Invitrogen #H3570) staining for nucleus detection.

### Nile Red staining

Nile red (Sigma-Aldrich #N3013) was dissolved in DMSO (Dimethyl Sulfoxide, Thermo Fisher Scientific # BP231-100) (0.5 mg/mL). Tissue sections on the slides were marked using hydrophobic marker and a drop of 50–100 µL of Nile red solution was applied to the slides. Slides were kept in the dark for 20 min. Then, slides were washed three times in PBS and mounting medium (VECTASHIELD® PLUS Antifade Mounting Medium with DAPI, Vector Labs #H-2000) was applied to the slide. Images were developed using confocal fluorescence microscope (Zeiss LSM 900 + Airyscan 2).

### Immunohistochemical and immunofluorescence staining

Heat-induced antigen retrieval was performed on tissues after deparaffinization and dehydration using 0.01 M sodium citrate (Sodium Citrate Dihydrate, Thermo Fisher Scientific #BP327-500) (pH 6.0) at 95 °C for 25 min followed by cooling on ice for 30 min. Suppression of peroxidase activity was done by incubating tissue slides in 3% H_2_O_2_ (Hydrogen Peroxide 30%, Thermo Fisher Scientific #H325-500) on ice for 20 min. Following blocking with 3% BSA/TBST + 5% goat (Normal Goat Serum Blocking Solution, Vector Labs #S-1000-20) or horse (Normal Horse Serum Blocking Solution, Vector Labs #S-2000-20) serum, the slides were incubated with primary antibody at 4 °C overnight. Slides were then incubated at RT with appropriate secondary antibodies (ImmPRESS HRP Goat Anti-Rabbit, Vector Labs #MP-7451) or (ImmPRESS HRP Horse Anti-Rabbit, Vector Labs #MP-7401) for 1 h. DAB Substrate Kit, Peroxidase (HRP), with Nickel, (3,3’-diaminobenzidine) (Vector Labs #SK-4100) was used to develop the signal, and hematoxylin was used for counterstaining.

### Primary antibodies and concentrations

Antibodies were diluted as follows: Rabbit anti-Olfm4 at 1:100 (Cell Signaling), Rabbit anti-Lysozyme at 1:5000 (Dako Cytomation), Rabbit anti-Ki67 at 1:100-200 (Novocastra Laboratories Ltd.), Rabbit anti-cleaved caspase-3 at 1:100 (Cell Signaling), Rabbit anti-Muc2 at 1:5000 (cell Signaling), Rabbit anti-Chromogranin A at 1:5000 (Immunostar), Rabbit anti-Dclk1 1:5000 (ab31704 Abcam), Rabbit anti p-S6 1:300–1000 (Cell Signaling), Rabbit anti FoxO1 1:100 (Cell Signaling) and Rabbit anti Tsc1 1:100 (Cell Signaling).

### Organoid staining

After drug treatments, the media were removed, and organoids were collected in cold AdDMEM/F-12 fresh media using 1 mL filter tips. Organoids were washed twice, kept on ice for 5 min after each wash to dissolve the Matrigel. For fixation, organoids were incubated in 4% PFA for 1 h at RT. Organoids were then blocked and permeabilized all at once by incubating them with (5% BSA + 0.3% Triton-X) at RT. Organoids were washed once with 1% BSA and then incubated with primary antibody diluted in (2.5% BSA + 0.1% Triton-X) overnight at 4 °C, followed by 3 times wash in 1% BSA and incubation with Alexa Fluor secondary antibody (Goat anti-Rabbit IgG Alexa Fluor™ 594, Invitrogen #A-11012) at a dilution of 1:50–1:100 for 3 h at RT. Organoids were washed twice with 1% BSA and then incubated with Hoechst 33342 solution diluted 1:1000 in 1% BSA solution for 15 min, followed by 3 times wash with 1% BSA solution. Individual organoids were picked and moved to specific plates for microscopic investigation. Image analysis was performed using Zeiss LSM 700 confocal microscope.

### Image quantification

Microscopic images were processed with Adobe Photoshop 7.0 and analyzed by the software ImageJ (NIH); cells were counted, and the intensity of the staining was measured according to this protocol^102^. Villus lengths, crypt depths, organoid areas, and lengths of the small and large intestines were all measured from digital images using ImageJ.

### Statistical analysis

Representative values in the figures are expressed as the mean ± SD. Sample sizes are mentioned in the respective figure legends. Data are presented as mean ± SD. Statistical significance was determined by two-tailed Student’s *t* test for pairwise comparisons or one-way or two-way ANOVA with either Tukey’s post-hoc or Sidak’s test for multiple comparisons. Differences were significant if the *P*- value was less than 0.05.

### RNA extraction and RNA-seq library preparation and sequencing

Small intestines were isolated from C57BL/6 mice (control, DKO, F-TKO, and T-TKO), rinsed with cold PBS, opened longitudinally, washed multiple times, and chopped into small fragments (5–10 mm). Tissue fragments were further washed in cold PBS, then incubated in 2 mM EDTA in PBS on a shaker at 4 °C for 20 min. After removing EDTA, tissue segments were washed three times in cold PBS and vigorously hand-shaken for 15–20 s. The supernatant was collected and passed through 300 μm cell strainers. This step was repeated twice, and the supernatant was centrifuged at 290 × *g* for 5 min. Pellets were resuspended in PBS and centrifuged at 100 × *g* for 3 min to eliminate single cells. Pellets were resuspended and pelleted again by centrifugation at 250 × *g* for 5 min. Final pellets were suspended in RNAlater (Invitrogen), kept at 4 °C overnight, and then stored at −80 °C until RNA extraction. RNA extraction was performed with the Maxwell® RSC simplyRNA Tissue Kit (Promega, AS1340). The extract was evaluated with Qubit™ RNA High Sensitivity (HS) Assay Kit (ThermoFisher, Q32855), Qubit™ 1X dsDNA High Sensitivity (HS) Assay Kit (ThermoFisher, Q33231), and RNA ScreenTape Analysis (Agilent, 5067-5576). Due to substantial DNA carryover, additional DNase treatment was performed with the RNase-Free DNase Set (Qiagen, 79254) and RNeasy Mini Cleanup Kit (Qiagen, 74116).

RNA-seq libraries were prepared with the NEXTFLEX Rapid Directional RNA-Seq Kit 2.0 (Revvity, NOVA-5198-53), with upstream enrichment for mRNA with NEXTFLEX Poly(A) Beads 2.0 (Revvity, NOVA-512995). Successful library preparation was confirmed on Illumina MiniSeq, then samples were rebalanced and pooled for sequencing on Illumina NovaSeq X on a 10B-cluster flow cell with 2 × 150 paired-end reads, aiming for 30 M clusters per sample.

### Reporting summary

Further information on research design is available in the Nature Portfolio Reporting Summary linked to this article.

## Supplementary information

Supplementary Information

Supplementary Dataset 1

Supplementary Dataset 2

Supplementary Dataset 3

Supplementary Dataset 4

Supplementary Dataset 5

Supplementary Dataset 6

Supplementary Dataset 7

Supplementary Dataset 8

Supplementary Dataset 9

Reporting Summary

Transparent Peer Review file

## Source data

Source Data 1

Source Data 2

## Data Availability

The RNA-seq data reported in this study are deposited in the NCBI under accession number: PRJNA1467868. https://www.ncbi.nlm.nih.gov/sra/PRJNA1467868. Source data are provided with this paper.

## References

[1] Chen, W. S. et al. Growth retardation and increased apoptosis in mice with homozygous disruption of the Akt1 gene. *Genes Dev.* **15**, 2203–2208 10.1101/gad.913901 (2001). PMC312770 11544177

[2] Cho, H., Thorvaldsen, J. L., Chu, Q., Feng, F. & Birnbaum, M. J. Akt1/PKBalpha is required for normal growth but dispensable for maintenance of glucose homeostasis in mice. *J. Biol. Chem.* **276**, 38349–38352 10.1074/jbc.C100462200 (2001). 11533044

[3] Cho, H. et al. Insulin resistance and a diabetes mellitus-like syndrome in mice lacking the protein kinase Akt2 (PKB beta). *Science* **292**, 1728–1731 10.1126/science.292.5522.1728 (2001). 11387480

[4] Easton, R. M. et al. Role for Akt3/protein kinase Bgamma in attainment of normal brain size. *Mol. Cell Biol.* **25**, 1869–1878 10.1128/MCB.25.5.1869-1878.2005 (2005). PMC549378 15713641

[5] Tschopp, O. et al. Essential role of protein kinase B gamma (PKB gamma/Akt3) in postnatal brain development but not in glucose homeostasis. *Development* **132**, 2943–2954 10.1242/dev.01864 (2005). 15930105

[6] Peng, X. D. et al. Dwarfism, impaired skin development, skeletal muscle atrophy, delayed bone development, and impeded adipogenesis in mice lacking Akt1 and Akt2. *Genes Dev.* **17**, 1352–1365 10.1101/gad.1089403 (2003). PMC196068 12782654

[7] Yang, Z. Z. et al. Dosage-dependent effects of Akt1/protein kinase Balpha (PKBalpha) and Akt3/PKBgamma on thymus, skin, and cardiovascular and nervous system development in mice. *Mol. Cell Biol.* **25**, 10407–10418 10.1128/mcb.25.23.10407-10418.2005 (2005). PMC1291243 16287854

[8] Wang, Q. et al. Spontaneous hepatocellular carcinoma after the combined deletion of Akt isoforms. *Cancer Cell* **29**, 523–535 10.1016/j.ccell.2016.02.008 (2016). PMC4921241 26996309

[9] Barker, N. et al. Identification of stem cells in small intestine and colon by marker gene Lgr5. *Nature* **449**, 1003–1007 10.1038/nature06196 (2007). 17934449

[10] Scoville, D. H., Sato, T., He, X. C. & Li, L. Current view: intestinal stem cells and signaling. *Gastroenterology* **134**, 849–864 10.1053/j.gastro.2008.01.079 (2008). 18325394

[11] Clevers, H. Shifting paradigms in tissue stem cell biology: Insights from the intestine. *Cell* **189**, 706–724 10.1016/j.cell.2025.12.025 (2026). 41650940

[12] Lynch, E. B. et al. Phosphatidylinositol 3-kinase signaling enhances intestinal crypt epithelial cell recovery after radiation. *Am. J. Pathol.* **195**, 1411–1427 10.1016/j.ajpath.2025.04.010 (2025). PMC12405924 40316215

[13] He, G. W. et al. Optimized human intestinal organoid model reveals interleukin-22-dependency of Paneth cell formation. *Cell Stem Cell* **29**, 1718–1720 10.1016/j.stem.2022.11.001 (2022). PMC9746670 36459971

[14] Schmitt, M. et al. Paneth cells respond to inflammation and contribute to tissue regeneration by acquiring stem-like features through SCF/c-Kit signaling. *Cell Rep.* **24**, 2312–2328 e2317 10.1016/j.celrep.2018.07.085 (2018). 30157426

[15] Reilly, L. et al. Loss of PI3k activity of inositol polyphosphate multikinase impairs PDK1-mediated AKT activation, cell migration, and intestinal homeostasis. *iScience* **26**, 106623 10.1016/j.isci.2023.106623 (2023). PMC10197106 37216099

[16] Lee, G. et al. Phosphoinositide 3-kinase signaling mediates beta-catenin activation in intestinal epithelial stem and progenitor cells in colitis. *Gastroenterology* **139**, 869–881 10.1053/j.gastro.2010.05.037 (2010). 881 e861-869. PMC2930080 20580720

[17] Laprise, P. et al. Phosphatidylinositol 3-kinase controls human intestinal epithelial cell differentiation by promoting adherens junction assembly and p38 MAPK activation. *J. Biol. Chem.* **277**, 8226–8234 10.1074/jbc.M110235200 (2002). 11756422

[18] Sheng, H., Shao, J., Townsend, C. M. Jr. & Evers, B. M. Phosphatidylinositol 3-kinase mediates proliferative signals in intestinal epithelial cells. *Gut* **52**, 1472–1478 10.1136/gut.52.10.1472 (2003). PMC1773820 12970141

[19] Faller, W. J. et al. mTORC1-mediated translational elongation limits intestinal tumour initiation and growth. *Nature* **517**, 497–500 10.1038/nature13896 (2015). PMC4304784 25383520

[20] Ludikhuize, M. C. et al. Mitochondria define intestinal stem cell differentiation downstream of a FOXO/Notch axis. *Cell Metab.* **32**, 889–900 e887 10.1016/j.cmet.2020.10.005 (2020). 33147486

[21] Wu, X., Kim, H., Jang, W. & Kim, C. Hipk transduces nutrient signals to control intestinal stem cell proliferation and fate in Drosophila. *Sci. Rep.* 10.1038/s41598-026-45137-5 (2026). PMC13168291 41876746

[22] Andrikopoulou, A. et al. The emerging role of capivasertib in breast cancer. *Breast* **63**, 157–167 10.1016/j.breast.2022.03.018 (2022). PMC9011110 35398754

[23] Breal, C. et al. Management of gastro-intestinal toxicity of the Pi3 kinase inhibitor: optimizing future dosing strategies. *Cancers* **15**, 10.3390/cancers15082279 (2023). PMC10136502 37190206

[24] Manning, B. D. & Toker, A. AKT/PKB signaling: navigating the network. *Cell* **169**, 381–405 10.1016/j.cell.2017.04.001 (2017). PMC5546324 28431241

[25] Hay, N. Interplay between FOXO, TOR, and Akt. *Biochim. Biophys. Acta* **1813**, 1965–1970 10.1016/j.bbamcr.2011.03.013 (2011). PMC3427795 21440577

[26] el Marjou, F. et al. Tissue-specific and inducible Cre-mediated recombination in the gut epithelium. *Genesis* **39**, 186–193 10.1002/gene.20042 (2004). 15282745

[27] Sato, T. et al. Single Lgr5 stem cells build crypt-villus structures in vitro without a mesenchymal niche. *Nature* **459**, 262–265 10.1038/nature07935 (2009). 19329995

[28] Barker, N. et al. Lgr5(+ve) stem cells drive self-renewal in the stomach and build long-lived gastric units in vitro. *Cell Stem Cell* **6**, 25–36 10.1016/j.stem.2009.11.013 (2010). 20085740

[29] Tan, S. H. et al. A constant pool of Lgr5(+) intestinal stem cells is required for intestinal homeostasis. *Cell Rep.* **34**, 108633 10.1016/j.celrep.2020.108633 (2021). 33503423

[30] Schuijers, J. et al. Ascl2 acts as an R-spondin/Wnt-responsive switch to control stemness in intestinal crypts. *Cell Stem Cell* **16**, 158–170 10.1016/j.stem.2014.12.006 (2015). 25620640

[31] Clevers, H. The intestinal crypt, a prototype stem cell compartment. *Cell* **154**, 274–284 10.1016/j.cell.2013.07.004 (2013). 23870119

[32] Formeister, E. J. et al. Distinct SOX9 levels differentially mark stem/progenitor populations and enteroendocrine cells of the small intestine epithelium. *Am. J. Physiol. Gastrointest. Liver Physiol.* **296**, G1108–G1118 10.1152/ajpgi.00004.2009 (2009). PMC2696217 19228882

[33] Snippert, H. J. et al. Intestinal crypt homeostasis results from neutral competition between symmetrically dividing Lgr5 stem cells. *Cell* **143**, 134–144 10.1016/j.cell.2010.09.016 (2010). 20887898

[34] Beumer, J. & Clevers, H. Cell fate specification and differentiation in the adult mammalian intestine. *Nat. Rev. Mol. Cell Biol.* **22**, 39–53 10.1038/s41580-020-0278-0 (2021). 32958874

[35] Ireland, H., Houghton, C., Howard, L. & Winton, D. J. Cellular inheritance of a Cre-activated reporter gene to determine Paneth cell longevity in the murine small intestine. *Dev. Dyn.* **233**, 1332–1336 10.1002/dvdy.20446 (2005). 15937933

[36] Gerkins, C., Hajjar, R., Oliero, M. & Santos, M. M. Assessment of gut barrier integrity in mice using fluorescein-isothiocyanate-labeled dextran. *J. Vis. Exp.* 10.3791/64710 (2022). 36468715

[37] Di Vincenzo, F., Del Gaudio, A., Petito, V., Lopetuso, L. R. & Scaldaferri, F. Gut microbiota, intestinal permeability, and systemic inflammation: a narrative review. *Intern. Emerg. Med.* **19**, 275–293 10.1007/s11739-023-03374-w (2024). PMC10954893 37505311

[38] Zhao, Z. et al. Organoids. *Nat. Rev. Methods Primers* **2** 10.1038/s43586-022-00174-y (2022). PMC10270325 37325195

[39] Puschhof, J. et al. Intestinal organoid cocultures with microbes. *Nat. Protoc.* **16**, 4633–4649 10.1038/s41596-021-00589-z (2021). 34381208

[40] Kim, J., Koo, B. K. & Knoblich, J. A. Human organoids: model systems for human biology and medicine. *Nat. Rev. Mol. Cell Biol.* **21**, 571–584 10.1038/s41580-020-0259-3 (2020). PMC7339799 32636524

[41] Heo, I. et al. Modelling Cryptosporidium infection in human small intestinal and lung organoids. *Nat. Microbiol.* **3**, 814–823 10.1038/s41564-018-0177-8 (2018). PMC6027984 29946163

[42] Yin, X. et al. Engineering stem cell organoids. *Cell Stem Cell* **18**, 25–38 10.1016/j.stem.2015.12.005 (2016). PMC4728053 26748754

[43] Clevers, H. Modeling development and disease with organoids. *Cell* **165**, 1586–1597 10.1016/j.cell.2016.05.082 (2016). 27315476

[44] Beumer, J. et al. Enteroendocrine cells switch hormone expression along the crypt-to-villus BMP signalling gradient. *Nat. Cell Biol.* **20**, 909–916 10.1038/s41556-018-0143-y (2018). PMC6276989 30038251

[45] Metcalfe, C., Kljavin, N. M., Ybarra, R. & de Sauvage, F. J. Lgr5+ stem cells are indispensable for radiation-induced intestinal regeneration. *Cell Stem Cell* **14**, 149–159 10.1016/j.stem.2013.11.008 (2014). 24332836

[46] Tian, H. et al. A reserve stem cell population in small intestine renders Lgr5-positive cells dispensable. *Nature* **478**, 255–259 10.1038/nature10408 (2011). PMC4251967 21927002

[47] Qi, Z. et al. BMP restricts stemness of intestinal Lgr5(+) stem cells by directly suppressing their signature genes. *Nat. Commun.* **8**, 13824 10.1038/ncomms13824 (2017). PMC5227110 28059064

[48] Martini, E. et al. Loss of survivin in intestinal epithelial progenitor cells leads to mitotic catastrophe and breakdown of gut immune homeostasis. *Cell Rep.* **14**, 1062–1073 10.1016/j.celrep.2016.01.010 (2016). 26832409

[49] Kim, T. H., Escudero, S. & Shivdasani, R. A. Intact function of Lgr5 receptor-expressing intestinal stem cells in the absence of Paneth cells. *Proc. Natl. Acad. Sci. USA* **109**, 3932–3937 10.1073/pnas.1113890109 (2012). PMC3309789 22355124

[50] Yang, L. et al. CREPT is required for murine stem cell maintenance during intestinal regeneration. *Nat. Commun.* **12**, 270 10.1038/s41467-020-20636-9 (2021). PMC7801528 33431892

[51] Tetteh, P. W. et al. Replacement of lost Lgr5-positive stem cells through plasticity of their enterocyte-lineage daughters. *Cell Stem Cell* **18**, 203–213 10.1016/j.stem.2016.01.001 (2016). 26831517

[52] Yan, K. S. et al. The intestinal stem cell markers Bmi1 and Lgr5 identify two functionally distinct populations. *Proc. Natl. Acad. Sci. USA* **109**, 466–471 10.1073/pnas.1118857109 (2012). PMC3258636 22190486

[53] Capdevila, C. et al. Time-resolved fate mapping identifies the intestinal upper crypt zone as an origin of Lgr5+ crypt base columnar cells. *Cell* **187**, 3039–3055 e3014 10.1016/j.cell.2024.05.001 (2024). PMC11770878 38848677

[54] Chen, Z. et al. Foxo1 controls gut homeostasis and commensalism by regulating mucus secretion. *J. Exp. Med.* **218**, 10.1084/jem.20210324 (2021). PMC8424467 34287641

[55] Bouchi, R. et al. FOXO1 inhibition yields functional insulin-producing cells in human gut organoid cultures. *Nat. Commun.* **5**, 4242 10.1038/ncomms5242 (2014). PMC4083475 24979718

[56] Chen, C. C. et al. FoxOs inhibit mTORC1 and activate Akt by inducing the expression of Sestrin3 and Rictor. *Dev. Cell* **18**, 592–604 10.1016/j.devcel.2010.03.008 (2010). PMC3031984 20412774

[57] Sanchez-Gurmaches, J., Martinez Calejman, C., Jung, S. M., Li, H. & Guertin, D. A. Brown fat organogenesis and maintenance requires AKT1 and AKT2. *Mol. Metab.* **23**, 60–74 10.1016/j.molmet.2019.02.004 (2019). PMC6480051 30833219

[58] Shearin, A. L., Monks, B. R., Seale, P. & Birnbaum, M. J. Lack of AKT in adipocytes causes severe lipodystrophy. *Mol. Metab.* **5**, 472–479 10.1016/j.molmet.2016.05.006 (2016). PMC4921941 27408773

[59] Nogueira, V. et al. Akt-dependent Skp2 mRNA translation is required for exiting contact inhibition, oncogenesis, and adipogenesis. *EMBO J.* **31**, 1134–1146 10.1038/emboj.2011.478 (2012). PMC3297989 22307088

[60] Chen, W. S. et al. Leptin deficiency and beta-cell dysfunction underlie type 2 diabetes in compound Akt knockout mice. *Mol. Cell Biol.* **29**, 3151–3162 10.1128/MCB.01792-08 (2009). PMC2681997 19289493

[61] Lin, A. et al. The FoxO-BNIP3 axis exerts a unique regulation of mTORC1 and cell survival under energy stress. *Oncogene* **33**, 3183–3194 10.1038/onc.2013.273 (2014). PMC4365448 23851496

[62] Harrington, L. S. et al. The TSC1-2 tumor suppressor controls insulin-PI3K signaling via regulation of IRS proteins. *J. Cell Biol.* **166**, 213–223 10.1083/jcb.200403069 (2004). PMC2172316 15249583

[63] Manning, B. D. Balancing Akt with S6K: implications for both metabolic diseases and tumorigenesis. *J. Cell Biol.* **167**, 399–403 10.1083/jcb.200408161 (2004). PMC2172491 15533996

[64] Hsu, P. P. et al. The mTOR-regulated phosphoproteome reveals a mechanism of mTORC1-mediated inhibition of growth factor signaling. *Science* **332**, 1317–1322 10.1126/science.1199498 (2011). PMC3177140 21659604

[65] Yu, Y. et al. Phosphoproteomic analysis identifies Grb10 as an mTORC1 substrate that negatively regulates insulin signaling. *Science* **332**, 1322–1326 10.1126/science.1199484 (2011). PMC3195509 21659605

[66] Skeen, J. E. et al. Akt deficiency impairs normal cell proliferation and suppresses oncogenesis in a p53-independent and mTORC1-dependent manner. *Cancer Cell* **10**, 269–280 10.1016/j.ccr.2006.08.022 (2006). 17045205

[67] Kwiatkowski, D. J. & Manning, B. D. Tuberous sclerosis: a GAP at the crossroads of multiple signaling pathways. *Hum. Mol. Genet.* **14**, R251–R258 10.1093/hmg/ddi260 (2005). 16244323

[68] Crino, P. B., Nathanson, K. L. & Henske, E. P. The tuberous sclerosis complex. *N. Engl. J. Med.* **355**, 1345–1356 10.1056/NEJMra055323 (2006). 17005952

[69] Curatolo, P. & Bombardieri, R. Tuberous sclerosis. *Handb. Clin. Neurol.* **87**, 129–151 10.1016/S0072-9752(07)87009-6 (2008). 18809023

[70] Menon, S. et al. Spatial control of the TSC complex integrates insulin and nutrient regulation of mTORC1 at the lysosome. *Cell* **156**, 771–785 10.1016/j.cell.2013.11.049 (2014). PMC4030681 24529379

[71] Kwiatkowski, D. J. et al. A mouse model of TSC1 reveals sex-dependent lethality from liver hemangiomas, and up-regulation of p70S6 kinase activity in Tsc1 null cells. *Hum. Mol. Genet.* **11**, 525–534 10.1093/hmg/11.5.525 (2002). 11875047

[72] Zhou, Y., Rychahou, P., Wang, Q., Weiss, H. L. & Evers, B. M. TSC2/mTORC1 signaling controls Paneth and goblet cell differentiation in the intestinal epithelium. *Cell Death Dis.* **6**, e1631 10.1038/cddis.2014.588 (2015). PMC4669793 25654764

[73] Webb, A. E., Kundaje, A. & Brunet, A. Characterization of the direct targets of FOXO transcription factors throughout evolution. *Aging Cell* **15**, 673–685 10.1111/acel.12479 (2016). PMC4933671 27061590

[74] Bohin, N. et al. Insulin-like growth factor-1 and mTORC1 signaling promote the intestinal regenerative response after irradiation injury. *Cell Mol. Gastroenterol. Hepatol.* **10**, 797–810 10.1016/j.jcmgh.2020.05.013 (2020). PMC7502577 32502530

[75] Sampson, L. L. mTOR disruption causes intestinal epithelial cell defects and intestinal atrophy postinjury in mice. *FASEB J. Res. Commun.* **30** 10.1096/fj.15-278606 (2016). PMC4750410 26631481

[76] Nenci, A. et al. Epithelial NEMO links innate immunity to chronic intestinal inflammation. *Nature* **446**, 557–561 10.1038/nature05698 (2007). 17361131

[77] Diez-Sanchez, A. et al. LSD1 drives intestinal epithelial maturation and controls small intestinal immune cell composition independent of microbiota in a murine model. *Nat. Commun.* **15**, 3412 10.1038/s41467-024-47815-2 (2024). PMC11035651 38649356

[78] Al-Sadi, R. et al. MMP-9-induced increase in intestinal epithelial tight permeability is mediated by p38 kinase signaling pathway activation of MLCK gene. *Am. J. Physiol. Gastrointest. Liver Physiol.* **316**, G278–G290 10.1152/ajpgi.00126.2018 (2019). PMC6397336 30543452

[79] Wirtz, S. et al. Chemically induced mouse models of acute and chronic intestinal inflammation. *Nat. Protoc.* **12**, 1295–1309 10.1038/nprot.2017.044 (2017). 28569761

[80] Rigby, R. J. et al. Intestinal bacteria are necessary for doxorubicin-induced intestinal damage but not for doxorubicin-induced apoptosis. *Gut Microbes* **7**, 414–423 10.1080/19490976.2016.1215806 (2016). PMC5046166 27459363

[81] Goode, E. C., Warburton, R. C., Gelson, W. T. & Watson, A. J. Activated intestinal macrophages in patients with cirrhosis release NO and IL-6 that may disrupt intestinal barrier function. *Gastroenterology* **145**, 1481–1484 10.1053/j.gastro.2013.10.031 (2013). 24409494

[82] Patankar, J. V. & Becker, C. Cell death in the gut epithelium and implications for chronic inflammation. *Nat. Rev. Gastroenterol. Hepatol.* **17**, 543–556 10.1038/s41575-020-0326-4 (2020). 32651553

[83] van Es, J. H. et al. Dll1+ secretory progenitor cells revert to stem cells upon crypt damage. *Nat. Cell Biol.* **14**, 1099–1104 10.1038/ncb2581 (2012). PMC3789123 23000963

[84] Wallaeys, C., Garcia-Gonzalez, N. & Libert, C. Paneth cells as the cornerstones of intestinal and organismal health: a primer. *EMBO Mol. Med* **15**, e16427 10.15252/emmm.202216427 (2023). PMC9906427 36573340

[85] Jadhav, U. et al. Dynamic reorganization of chromatin accessibility signatures during dedifferentiation of secretory precursors into Lgr5+ intestinal stem cells. *Cell Stem Cell* **21**, 65–77 e65 10.1016/j.stem.2017.05.001 (2017). PMC5505276 28648363

[86] Yu, S. et al. Paneth cell multipotency induced by notch activation following injury. *Cell Stem Cell* **23**, 46–59 e45 10.1016/j.stem.2018.05.002 (2018). PMC6035085 29887318

[87] Shimokawa, M. et al. Visualization and targeting of LGR5(+) human colon cancer stem cells. *Nature* **545**, 187–192 10.1038/nature22081 (2017). 28355176

[88] Buczacki, S. J. et al. Intestinal label-retaining cells are secretory precursors expressing Lgr5. *Nature* **495**, 65–69 10.1038/nature11965 (2013). 23446353

[89] Montgomery, R. K. et al. Mouse telomerase reverse transcriptase (mTert) expression marks slowly cycling intestinal stem cells. *Proc. Natl. Acad. Sci. USA* **108**, 179–184 10.1073/pnas.1013004108 (2011). PMC3017192 21173232

[90] Powell, A. E. et al. The pan-ErbB negative regulator Lrig1 is an intestinal stem cell marker that functions as a tumor suppressor. *Cell* **149**, 146–158 10.1016/j.cell.2012.02.042 (2012). PMC3563328 22464327

[91] Sangiorgi, E. & Capecchi, M. R. Bmi1 is expressed in vivo in intestinal stem cells. *Nat. Genet.* **40**, 915–920 10.1038/ng.165 (2008). PMC2906135 18536716

[92] Takeda, N. et al. Interconversion between intestinal stem cell populations in distinct niches. *Science* **334**, 1420–1424 10.1126/science.1213214 (2011). PMC3705713 22075725

[93] Asfaha, S. et al. Krt19(+)/Lgr5(-) cells are radioresistant cancer-initiating stem cells in the colon and intestine. *Cell Stem Cell* **16**, 627–638 10.1016/j.stem.2015.04.013 (2015). PMC4457942 26046762

[94] Yousefi, M., Li, L. & Lengner, C. J. Hierarchy and plasticity in the intestinal stem cell compartment. *Trends Cell Biol.* **27**, 753–764 10.1016/j.tcb.2017.06.006 (2017). PMC5612891 28732600

[95] Mustata, R. C. et al. Lgr4 is required for Paneth cell differentiation and maintenance of intestinal stem cells ex vivo. *EMBO Rep.* **12**, 558–564 10.1038/embor.2011.52 (2011). PMC3128273 21508962

[96] Murata, K. et al. Ascl2-dependent cell dedifferentiation drives regeneration of ablated intestinal stem cells. *Cell Stem Cell* **26**, 377–390 e376 10.1016/j.stem.2019.12.011 (2020). PMC7147146 32084390

[97] Seishima, R. & Barker, N. A contemporary snapshot of intestinal stem cells and their regulation. *Differentiation* **108**, 3–7 10.1016/j.diff.2019.01.004 (2019). 30711339

[98] Leushacke, M. et al. Lgr5-expressing chief cells drive epithelial regeneration and cancer in the oxyntic stomach. *Nat. Cell Biol.* **19**, 774–786 10.1038/ncb3541 (2017). 28581476

[99] Schuijers, J., van der Flier, L. G., van Es, J. & Clevers, H. Robust cre-mediated recombination in small intestinal stem cells utilizing the olfm4 locus. *Stem Cell Rep.* **3**, 234–241 10.1016/j.stemcr.2014.05.018 (2014). PMC4175542 25254337

[100] Leavens, K. F., Easton, R. M., Shulman, G. I., Previs, S. F. & Birnbaum, M. J. Akt2 is required for hepatic lipid accumulation in models of insulin resistance. *Cell Metab.* **10**, 405–418 10.1016/j.cmet.2009.10.004 (2009). PMC2796129 19883618

[101] Wan, M. et al. Loss of Akt1 in mice increases energy expenditure and protects against diet-induced obesity. *Mol. Cell Biol.* **32**, 96–106 10.1128/MCB.05806-11 (2012). PMC3255699 22037765

[102] Crowe, A. R. & Yue, W. Semi-quantitative determination of protein expression using immunohistochemistry staining and analysis: an integrated protocol. *Bio Protoc.* **9** 10.21769/BioProtoc.3465 (2019). PMC6924920 31867411

