# Supplementary material for: Loss of intestinal Akt elicits rapid mortality via intestinal damage which is rescued by mTORC1 activation
Source: Nat Commun. 2026 Sep 23;17:11057. doi: 10.1038/s41467-026-77283-9 (PMC13690743; doi:10.1038/s41467-026-77283-9)
Supplement: Supplementary file 1 [file 41467_2026_77283_MOESM11_ESM.pdf]

nature portfolio

Corresponding author(s): *Double-anonymous peer review submissions: write DAPR and your manuscript number here instead of author names.*

Last updated by author(s): *YYYY-MM-DD*

Reporting Summary

Nature Portfolio wishes to improve the reproducibility of the work that we publish. This form provides structure for consistency and transparency in reporting. For further information on Nature Portfolio policies, see our Editorial Policies and the Editorial Policy Checklist.

Statistics

For all statistical analyses, confirm that the following items are present in the figure legend, table legend, main text, or Methods section.

n/a	Confirmed	
☐	☒	The exact sample size (*n*) for each experimental group/condition, given as a discrete number and unit of measurement
☐	☒	A statement on whether measurements were taken from distinct samples or whether the same sample was measured repeatedly
☐	☒	The statistical test(s) used AND whether they are one- or two-sided<br>*Only common tests should be described solely by name; describe more complex techniques in the Methods section.*
☒	☐	A description of all covariates tested
☒	☐	A description of any assumptions or corrections, such as tests of normality and adjustment for multiple comparisons
☐	☒	A full description of the statistical parameters including central tendency (e.g. means) or other basic estimates (e.g. regression coefficient) AND variation (e.g. standard deviation) or associated estimates of uncertainty (e.g. confidence intervals)
☐	☒	For null hypothesis testing, the test statistic (e.g. *F*, *t*, *r*) with confidence intervals, effect sizes, degrees of freedom and *P* value noted<br>*Give P values as exact values whenever suitable.*
☒	☐	For Bayesian analysis, information on the choice of priors and Markov chain Monte Carlo settings
☒	☐	For hierarchical and complex designs, identification of the appropriate level for tests and full reporting of outcomes
☒	☐	Estimates of effect sizes (e.g. Cohen's *d*, Pearson's *r*), indicating how they were calculated

Our web collection on statistics for biologists contains articles on many of the points above.

Software and code

Policy information about availability of computer code

Data collection	ImageJ Schneider et al., 2012. https://imagej.net/ij/.GraphPad Prism version 8.4.2 Prism www.graphpad.com<br>Cryostat (CM1950) Leica Biosystems N/A<br>Inverted microscope (DMi8) Leica Biosystems N/A<br>Confocal fluorescence microscope (LSM 900 with Airyscan 2) Zeiss N/A<br>Confocal fluorescence microscope (LSM 700) Zeiss N/A<br>Enhanced chemiluminescence detection system Azure Biosystems N/A<br>Glucometer (Contour Next Blood Glucose Ascensia # 00193-7090-21<br>Monitoring System
Data analysis	GraphPad Prism version 8.4.2 Prism www.graphpad.com

For manuscripts utilizing custom algorithms or software that are central to the research but not yet described in published literature, software must be made available to editors and reviewers. We strongly encourage code deposition in a community repository (e.g. GitHub). See the Nature Portfolio guidelines for submitting code & software for further information.

Data

Policy information about availability of data

All manuscripts must include a data availability statement. This statement should provide the following information, where applicable:

- Accession codes, unique identifiers, or web links for publicly available datasets
- A description of any restrictions on data availability
- For clinical datasets or third party data, please ensure that the statement adheres to our policy

The RNA-seq data reported in this study are deposited in the NCBI under accession number: PRJNA1467868. https://www.ncbi.nlm.nih.gov/sra/PRJNA1467868.
Source data are provided with this paper.

Research involving human participants, their data, or biological material

Policy information about studies with human participants or human data. See also policy information about sex, gender (identity/presentation), and sexual orientation and race, ethnicity and racism.

Reporting on sex and gender	NA
Reporting on race, ethnicity, or other socially relevant groupings	NA
Population characteristics	NA
Recruitment	NA
Ethics oversight	NA

Note that full information on the approval of the study protocol must also be provided in the manuscript.

Field-specific reporting

Please select the one below that is the best fit for your research. If you are not sure, read the appropriate sections before making your selection.

☒ Life sciences ☐ Behavioural & social sciences ☐ Ecological, evolutionary & environmental sciences

For a reference copy of the document with all sections, see nature.com/documents/nr-reporting-summary-flat.pdf

Life sciences study design

All studies must disclose on these points even when the disclosure is negative.

Sample size	Sample size was performed
Data exclusions	No data exclusion
Replication	All data were reproduced
Randomization	This was not relevant to the studies
Blinding	Visual analysis was performed blindly

Reporting for specific materials, systems and methods

We require information from authors about some types of materials, experimental systems and methods used in many studies. Here, indicate whether each material, system or method listed is relevant to your study. If you are not sure if a list item applies to your research, read the appropriate section before selecting a response.

Materials & experimental systems

n/a	Involved in the study	
☐	☒	Antibodies
☐	☒	Eukaryotic cell lines
☐	☐	Palaeontology and archaeology
☐	☒	Animals and other organisms
☐	☐	Clinical data
☐	☐	Dual use research of concern
☐	☐	Plants

Methods

n/a	Involved in the study	
☒	☐	ChIP-seq
☒	☐	Flow cytometry
☒	☐	MRI-based neuroimaging

Antibodies

Antibodies used

Rabbit anti-Olfm4 Cell Signaling Cat# 39141S
Rabbit anti-Lysozyme Dako Cat# A0099
Rabbit anti-Ki67 Abcam Cat# ab16667
Rabbit anti-Cleaved Caspase-3 Cell Signaling Cat# 9661S
Rabbit anti-Muc2 Abcam Cat# ab272692
Rabbit anti-Chromogranin A Proteintech Cat# 10529-1-AP
Rabbit anti-Dclk1 Abcam Cat# ab31704
Rabbit anti-CD3 Cell Signaling Cat# 78588S
Rabbit anti-CD11c Cell Signaling Cat# 97585S
Rabbit anti-LY-6G Cell Signaling Cat# 870485
Rabbit anti-F4/80 Cell Signaling Cat# 70076S
Rabbit anti-MRP8 (S100A8) Cell Signaling Cat# 47310S
Rabbit anti-pan-AKT Cell Signaling Cat# 4691L
Rabbit anti-pan-p-AKT XP Cell Signaling Cat# 4060L
Rabbit anti-AKT1 Cell Signaling Cat# 2938S
Rabbit anti-p-AKT1 specific (S473) Cell Signaling Cat# 9018S
Rabbit anti-AKT2 Cell Signaling Cat# 3063S
Rabbit anti-p-AKT2 specific (S474) Cell Signaling Cat# 8599
Rabbit anti-AKT3 Cell Signaling Cat# 14982S
Rabbit anti-PRAS40 Cell Signaling Cat# 2997S
Rabbit anti-p-PRAS40 Cell Signaling Cat# 2997S
Rabbit anti-S6 Cell Signaling Cat# 2217S
Rabbit anti-p-S6 Cell Signaling Cat# 2211L
Rabbit anti-Raptor Cell Signaling Cat# 2280S
Rabbit anti-FOXO1 Cell Signaling Cat# 2880S
Rabbit anti-p-FOXO1 Cell Signaling Cat# 9461S
Rabbit anti-β-actin Cell Signaling Cat# 4970
Rabbit anti GAPDH XP Cell Signaling Cat# 5174
Mouse anti-Vinculin Sigma-Aldrich Cat# V9131
Anti-Rabbit IgG (H+L) Secondary Antibody, HRP Invitrogen Cat# G21234
Anti-Mouse IgG (H+L) Secondary Antibody, HRP Invitrogen Cat# G21040
Goat Anti-Rabbit IgG Alexa Fluor™ 488 Invitrogen Cat# A11008
Goat Anti-Mouse IgG Alexa Fluor™ 488 Invitrogen Cat# A11001
Goat Anti-Rabbit IgG Alexa Fluor™ 594 Invitrogen Cat# A11012
Goat Anti-Mouse IgG Alexa Fluor™ 594 Invitrogen Cat# A11001

Validation

Antibodies were validated using knockout cells and tissues.

Eukaryotic cell lines

Policy information about cell lines and Sex and Gender in Research

Cell line source(s)	NA
Authentication	NA
Mycoplasma contamination	NA
Commonly misidentified lines (See ICLAC register)	NA

Palaeontology and Archaeology

Specimen provenance

Provide provenance information for specimens and describe permits that were obtained for the work (including the name of the

Specimen provenance | *issuing authority, the date of issue, and any identifying information). Permits should encompass collection and, where applicable, export.*

Specimen deposition | *Indicate where the specimens have been deposited to permit free access by other researchers.*

Dating methods | *If new dates are provided, describe how they were obtained (e.g. collection, storage, sample pretreatment and measurement), where they were obtained (i.e. lab name), the calibration program and the protocol for quality assurance OR state that no new dates are provided.*

☐ Tick this box to confirm that the raw and calibrated dates are available in the paper or in Supplementary Information.

Ethics oversight | *Identify the organization(s) that approved or provided guidance on the study protocol, OR state that no ethical approval or guidance was required and explain why not.*

Note that full information on the approval of the study protocol must also be provided in the manuscript.

Animals and other research organisms

Policy information about studies involving animals; ARRIVE guidelines recommended for reporting animal research, and Sex and Gender in Research

Laboratory animals

Mouse C57BL/6: Akt1f/f; Akt1f/f; VillinCreERT2 This paper N/A
Mouse C57BL/6: Akt2-/-; Akt1f/f; VillinCreERT2 This paper N/A
Mouse C57BL/6: FoxO1f/f; VillinCreERT2 This paper N/A
Mouse C57BL/6: Akt1f/f; Akt2f/f; FoxO1f/f; VillinCreERT2 This paper N/A
Mouse C57BL/6: Akt2-/-; Akt1f/f; FoxO1f/f; VillinCreERT2 This paper N/A
Mouse C57BL/6: Tsc1f/f; VillinCreERT2 This paper N/A
Mouse C57BL/6: Akt1f/f; Akt2f/f; Tsc1f/f; VillinCreERT2 This paper N/A
Mouse C57BL/6: Akt2-/-; Akt1f/f; Tsc1f/f; VillinCreERT2 This paper N/A
Mouse C57BL/6: Rptorf/f; VillinCreERT2 This paper N/A
Mouse C57BL/6: Akt1f/f; Akt2f/f; Rosa26CreERT2 Wang et al., 2016 N/A
Mouse C57BL/6: Akt1f/f; Akt2f/f; FoxO1f/f; Rosa26CreERT2 This paper N/A
Mouse C57BL/6: Akt1f/f; Akt2f/f; Lgr5-EGFP-IRES-CreERT2 This paper N/A
Mouse C57BL/6: Akt2-/-; Akt1f/f; Lgr5-EGFP-IRES-CreERT2 This paper N/A
Mouse C57BL/6:Akt1f/f-; Akt2-/-; Lgr5-EGFP-IRES-CreERT2 This paper N/A

Wild animals | NA

Reporting on sex | NA

Field-collected samples | NA

Ethics oversight | Institutional Animal Care and Use Committee at the University of Illinois at Chicago

Note that full information on the approval of the study protocol must also be provided in the manuscript.

Clinical data

Policy information about clinical studies
All manuscripts should comply with the ICMJE guidelines for publication of clinical research and a completed CONSORT checklist must be included with all submissions.

Clinical trial registration | NA

Study protocol | NA

Data collection | NA

Outcomes | NA

Dual use research of concern

Policy information about dual use research of concern

Hazards

Could the accidental, deliberate or reckless misuse of agents or technologies generated in the work, or the application of information presented in the manuscript, pose a threat to:

No	Yes	
☒	☐	Public health
☒	☐	National security
☒	☐	Crops and/or livestock
☒	☐	Ecosystems
☒	☐	Any other significant area

Experiments of concern

Does the work involve any of these experiments of concern:

No	Yes	
☒	☐	Demonstrate how to render a vaccine ineffective
☒	☐	Confer resistance to therapeutically useful antibiotics or antiviral agents
☒	☐	Enhance the virulence of a pathogen or render a nonpathogen virulent
☒	☐	Increase transmissibility of a pathogen
☒	☐	Alter the host range of a pathogen
☒	☐	Enable evasion of diagnostic/detection modalities
☒	☐	Enable the weaponization of a biological agent or toxin
☒	☐	Any other potentially harmful combination of experiments and agents

Plants

Seed stocks	NA
Novel plant genotypes	NA
Authentication	NA
