# Supplementary material for: Loss of intestinal Akt elicits rapid mortality via intestinal damage which is rescued by mTORC1 activation
Source: Nat Commun. 2026 Sep 23;17:11057. doi: 10.1038/s41467-026-77283-9 (PMC13690743; doi:10.1038/s41467-026-77283-9)
Supplement: Supplementary file 2 [file 41467_2026_77283_MOESM12_ESM.pdf]

nature portfolio

Peer Review File

Loss of intestinal Akt elicits rapid mortality via intestinal damage which is rescued by mTORC1 activation

Corresponding Author: Professor Nissim Hay

This file contains all reviewer reports in order by version, followed by all author rebuttals in order by version.

Version 0:

Reviewer comments:

Reviewer #1

(Remarks to the Author)
In the paper entitled « Loss of intestinal Akt impairs intestinal homeostasis eliciting rapid mortality that is rescued by mTORC1 activation through FoxO1 or Tsc1 deletion », the authors provide some experimental evidence that mice lacking both Akt1/2 isoforms in the intestine suffer from severe damage in the intestinal architecture with a strong inflammatory phenotype. They also show that the genetic inactivation of both Akt1/2 isoforms leads to the loss of Paneth cells as well as to defects in the proliferation and survival of Lgr5+ and transit-amplifying cells. Importantly, they also show that the deletion of FoxO1 or Tsc1 in Akt1/2 double mutants rescue the phenotype and suggest that mTORC1 activation is critical to maintain intestinal integrity. Overall, the authors describe an interesting and strong intestinal phenotype seen upon the loss of both Akt1/2 isoforms. The conducted experiments are convincing and results are robust. However, the precise molecular mechanisms through which FoxO1 or Tsc1 deletion rescues the phenotype of Akt1/2 double mutants deserve more attention. The authors should address the following points to improve their study.

Major points
1. The introduction lacks a key paragragh in which functional links between Akt isoforms and FoxO1 are described. It is indeed essential to mention that Akt phosphorylates FoxO1 in response to insulin and growth factors. This phosphorylation of FoxO1 by Akt brings it from the nucleus to the cytoplasm in order to inactivate it. Same remark on mechanisms through which Tsc1 inhibits mTORC1. These mechanistical explanations are essential to better understand the phenotypes that will be later described in the manuscript. The authors provide this information in the result section (from lane 355) but I think that it should be introduced earlier in the manuscript. Some words on known Akt substrates should be added in the Introduction section : this is essential for the understanding of molecular mechanisms through which Akt1/2 isoforms promote intestinal homeostasis.

2. The introduction is also lacking a paragragh describing the biology of Lgr5+ cells. How do they ultimately generate all epithelial subtypes ? A few words should also describe each epithelial subtypes given the fact that authors will quantify them in the Result section. Generally speaking, the Introduction section is too short.

3. The authors use Oflm4 as a marker of Lgr5+ stem cells in intestinal crypts. It is also important to note that Olfm4 is a direct target of Notch signaling. Therefore, I think that it would also be interesting to carry out western blot analyses to assess Notch activation in all experimental conditions. Should we expect a defective Notch activation in Akt1/2 double mutants ? This information, if relevant, would add some mechanistical insights into this study.

4. In Figure S2G, the authors nicely show that the loss of both Akt1/2 isoforms in the intestine leads to the accumulation of various pro-inflammatory cytokines and chemokines, which underlies the severe inflammatory phenotype seen in these mutants. It is important to note that many of these candidates are regulated by NF-kappaB, a pro-inflammatory and pro-survival family of transcription factors. It is likely that damage in the intestinal architecture seen in Akt1/2 double mutants leads to the activation of Toll-like receptors (TLR) signaling and ultimately to NF-kappaB-dependent gene transcription. The authors should assess NF-kappaB activation by western blot (assessing p65 phosphorylation on Serine 536 is a good way to assess this). We may indeed expect higher levels of NF-kappaB activity in Akt1/2 double mutants.

5. For Figure 3E in which several pharmacological Akt inhibitors are tested, the authors assessed S6 phosphorylation. They should also assess nuclear versus cytoplasmic levels of FoxO1 given the fact that FoxO1 is a direct target of Akt. This could

actually be an elegant way to introduce this candidate in the study. We indeed expect FoxO1 to show a distinct subcellular localization in Akt1/2 double mutants versus WT mice as well as in mice treated with Akt inhibitors. Assessing nuclear and cytoplasmic levels of FoxO1 in both experimental models (i.e. in WT/KO mice as well as in mice treated or not with Akt inhibitors) would actually be an additional evidence to prove that phenotypes are comparable. Speaking of this, the authors show an IHC for FoxO1 in Figure 4A but it is difficult to judge this result. The authors should also carry out western blot analyses with nuclear versus cytoplasmic extracts in order to provide a more convincing result.

6. In control mice in which both Akt1/2 isoforms are expressed, we would then expect to see FoxO1 phosphorylation by both kinases. Can we detect FoxO1 phosphorylation in mice under homeostasis ? If so, in which cells ? Lgr5+ cells ? All epithelial subtypes ? Immunofluorescence studies with co-markers would be nice to do to explore this issues.

7. I did not find any information of mice only lacking FoxO1 in intestinal epithelial cells. This additional control should be carried out as one may say that the genetic inactivation of FoxO1 alone most likely leads to a phenotype. In this context, it is important to note that Chen and colleagues previously published that FoxO1 is important in gut homeostasis and also controls mucus secretion by goblet cells (JEM 2021, PMID 34287641). Findings described in this study should be discussed by authors. In this context, authors of the current study shows that the inactivation of FoxO1 in Akt1/2 double mutants enhances mTORC1 activity (as assessed by anti-pS6 levels by western blot). This important result raises two questions : first, what is happening on mTORC1 activity if we only inactivate FoxO1 in intestinal epithelial cells ? This important control has to be done. The second question is on molecular mechanisms through which mTORC1 activation is comparable in Ctrl versus F-TKO mice. Some explanations are required. The current authors previously published a nice study in which functional links between FoxO1 and mTORC1 activity were established. This study should be cited at the time authors introduce data on mTORC1 activity in the manuscript.

8. Changes in total S6 are seen in extracts from Ctrl versus DKO mice (Figure 4A) but those changes are not that dramatic anymore in data illustrated in Figure 6A. This inconsistency should be corrected.

9. Unless I missed something, I have not found Figure S6 in the PDF documents provided by the authors.

10. Generally speaking, it is not surprising that the loss of both Akt1/2 isoforms leads to cell death and to a severe phenotype in the intestine, given the key role of both isoforms in cell survival. Moreover, it is also expected that FoxO1 deficiency in Akt1/2 double mutants restore the phenotype given the known role of Akt on FoxO1 inhibition through phosphorylation. From a mechanistical point of view, there results, although properly executed, do not provide new mechanisms driving cell survival in the intestine. On the other hand, authors conclude on lane 477 that mTORC1 hyperactivation overrides FoxO1 activity despite its nuclear localization. This observation is much more novel from a mechanistical point of view but it is sad that authors did not explore this link further. Do we have any evidence that FoxO1 phosphorylation in T-TKO mice would change ? Any additional information on this new mechanistical link would be great to increase the impact of this study. This is, at least in my view, the main weakness of this study.

11. Akt1/2 isoforms are expected to target multiple substrates beside FoxO1. Although we understand that FoxO1 is a major candidate acting downstream of Akt1/2 in this study, authors should also discuss the possibility that additional substrates targeted by Akt1/2 may act as effectors of cell survival in the intestine.

Minor points

1. Molecular weights markers are lacking for western blot analyses.

2. pS6 levels assessed by western blot analyses are illustrated in Figure 4B but these results are not described. The authors should show these western blot later in the paper or describe them at that time.

Reviewer #2

(Remarks to the Author)
Comments to authors

The underlying mechanism of impaired intestinal homeostasis in Akt1/2 dKO mice remains unclear. The authors identified genetic epistasis by which FoxO1 or Tsc1 mediates Akt1/2 dKO-induced intestinal phenotypes. Additionally, the authors showed that genetic deletion of Raptor, a key component of mTORC1, phenocopies Akt1/2 dKO phenotypes. This is a straightforward study that extensively uses genetically engineered mouse models and covers a relatively short phenotypic analysis window of up to 8 days. However, the authors' discovery still has conceptual gaps and requires clarification and additional experimental evidence to support the proposed model better. Addressing the following comments will strengthen the authors' findings.

Major comments
A. Fig. 2F, 4L, . Instead of quantifying the area, the organoid-forming efficiency (OFE, at least up to 3 passages) can better address the impact of Akt1/2 cKO on the integrity and stemness of organoids, as shown in the results using a Lgr5CreERT2 driver. For Supplementary Figure S3A and D, please include additional information, i.e., organoid numbers instead of OFE.
B. Fig. 4A. Please include additional IHC images showing the localization change of FoxO1 from the cytosol to the nucleus. Also, include images from the crypts. This is because Akt1/2 dKO significantly affects ISCs and TAs in the crypts. Moreover,

FoxO1 in Figure 5E does not seem to be expressed in the nuclei or cells in the crypts. Please also improve the FoxO1 IHC to reduce background.
C. Fig. 1J, 3I, 4I, 5E. Muc2, a marker of goblet cells, showed inconsistent results among the phenotypes. Especially, dKO (5E) and dKO (1J) do not match. Based on Fig. 5E, the mucinous cell lineage was not affected by dKO. Also, Raptor cKO failed to inhibit Muc2 expression (Fig. 7F). Explain the inconsistency or repeat the experiments.
D. While the drug-treated mice develop diarrhea and intestinal shortening, these phenotypes could reflect non-specific systemic toxicity at the chosen dose rather than on-target PI3K/Akt inhibition in the intestinal epithelium. The authors should strengthen this point by providing basic functional readouts of intestinal injury. It would be helpful to include an independent PI3K/Akt inhibitor or include an unrelated compound at a comparably tolerated dose as a negative control for non-specific systemic toxicity.
E. The authors suggested that targeting FOXO1 may mitigate gastrointestinal toxicity associated with PI3K/Akt pathway inhibition in cancer therapy. However, because FOXO factors are frequently involved in tumor-suppressive stress and apoptotic programs downstream of PI3K/Akt blockade, systemic FOXO1 inhibition could compromise anti-tumor efficacy or have context-dependent oncogenic consequences. Please clarify the intended clinical scenario (which patient populations/indications for PI3K/Akt inhibitors) and discuss feasibility and safety considerations, including whether a gut-restricted/locally delivered protective strategy would be required and how tumor-specific FOXO1 alterations might affect this approach.
F. Given the broad regulatory function of Akt, the rationale for focusing exclusively on FoxO1 remains elusive. The authors should provide a comprehensive list of Akt targets in this model and demonstrate that FoxO1 is a key transcriptional factor in such dKO phenotypes. For instance, instead of showing the selected genes and heatmaps (S2G and S7B, C) from bulk RNA-seq, please perform GSEA, pathway, or regulon analyses to demonstrate activation of the FoxO1-related transcriptional circuit, beyond heatmap clustering. Additionally, intestinal cell lineages (Paneth, mucinous, neuroendocrine, TA, and ISCs [proliferative and quiescent]) can be examined by analyzing the RNA-seq datasets, strengthening the IHC results. Such unbiased results will strongly justify the authors' choice of FoxO1. This manuscript does not include information about the datasets (e.g., GEO accession number and a token for reviewers) or a data availability statement.

Minor comments
1. Line 335, Fig. 3I. Until showing the impact of inhibitors on Lgr5-positive cells using Lgr5CreERT2 mice (as a reporter), this sentence incorrectly describes the results. Olfm4 and others are ISC markers. However, the expression of them and Lgr5 could differ. Please revise the sentence.
2. Discussion can be improved by minimizing redundancy (repetition of results).
3. Please standardize the mouse strain nomenclature throughout the manuscript. The standard notation is C57BL/6 (with "BL" in uppercase) – line 103, 105.
4. In the introduction (around line 68), the in-text citations appear to be improperly formatted.
5. Please correct minor typographical issues, for example, "mTROC1" → "mTORC1" and "Tsc1deletion" → "Tsc1_deletion", and scan the manuscript for similar formatting or spacing errors (In the discussion around lines 570 and 573). FoxO1 v. FOXO1
6. Please standardize statistical reporting across all figure legends by explicitly stating what "n" represents and consistently listing the statistical test used and the error-bar definition (SD vs. SEM).
7. Please include scale bars for each image.
8. Fig. 4M. Replace it with better/clearer images.
9. The manuscript states that several key in vivo experiments were performed in male mice, whereas systemic deletion experiments included both sexes. Please clarify the rationale for using male-only cohorts in these experiments.
10. Figure S6 is missing.
11. The figure legends and labels need improvement for clarity. Please use markers (arrows, asterisks) and color-coded labels to highlight specific pathological features in IHC H&E images (e.g., Fig.4I/ Fig. S4H).

Version 1:

Reviewer comments:

Reviewer #1

(Remarks to the Author)
The authors experimentally addressed most of my comments and also updated the introduction section as requested. It is indeed very interesting to note that several canonical Notch target genes were significantly decreased in the small intestine of mice lacking intestinal Akt, which therefore establishes a functional link between Notch signaling and Akt deficiency. As some Wnt target genes are also downregulated in these mice, I found the paper more interesting/novel from a signaling point of view. Likewise, the authors now clarified molecular mechanisms through which Akt loss leads to intestinal and systemic inflammation as NF-kappaB-dependent gene transcription is potentiated in these mice. Overall and although some questions remain unclear (which happens for most if not all submitted manuscripts...), it is fair to say that this version is definitely more convincing.

Reviewer #2

(Remarks to the Author)
Most comments were addressed by new experimental results and clarification.

Open Access This Peer Review File is licensed under a Creative Commons Attribution 4.0 International License, which permits use, sharing, adaptation, distribution and reproduction in any medium or format, as long as you give appropriate credit to the original author(s) and the source, provide a link to the Creative Commons license, and indicate if changes were made.
In cases where reviewers are anonymous, credit should be given to 'Anonymous Referee' and the source.
The images or other third party material in this Peer Review File are included in the article’s Creative Commons license, unless indicated otherwise in a credit line to the material. If material is not included in the article’s Creative Commons license and your intended use is not permitted by statutory regulation or exceeds the permitted use, you will need to obtain permission directly from the copyright holder.
To view a copy of this license, visit https://creativecommons.org/licenses/by/4.0/

Reviewer #1 (Remarks to the Author)

In the paper entitled « Loss of intestinal Akt impairs intestinal homeostasis eliciting rapid mortality that is rescued by mTORC1 activation through FoxO1 or Tsc1 deletion », the authors provide some experimental evidence that mice lacking both Akt1/2 isoforms in the intestine suffer from severe damage in the intestinal architecture with a strong inflammatory phenotype. They also show that the genetic inactivation of both Akt1/2 isoforms leads to the loss of Paneth cells as well as to defects in the proliferation and survival of Lgr5+ and transit-amplifying cells. Importantly, they also show that the deletion of FoxO1 or Tsc1 in Akt1/2 double mutants rescue the phenotype and suggest that mTORC1 activation is critical to maintain intestinal integrity. Overall, the authors describe an interesting and strong intestinal phenotype seen upon the loss of both Akt1/2 isoforms. The conducted experiments are convincing and results are robust. However, the precise molecular mechanisms through which FoxO1 or Tsc1 deletion rescues the phenotype of Akt1/2 double mutants deserve more attention. The authors should address the following points to improve their study.

Major points

1. The introduction lacks a key paragraph in which functional links between Akt isoforms and FoxO1 are described. It is indeed essential to mention that Akt phosphorylates FoxO1 in response to insulin and growth factors. This phosphorylation of FoxO1 by Akt brings it from the nucleus to the cytoplasm in order to inactivate it. Same remark on mechanisms through which Tsc1 inhibits mTORC1. These mechanistical explanations are essential to better understand the phenotypes that will be later described in the manuscript. The authors provide this information in the result section (from lane 355) but I think that it should be introduced earlier in the manuscript. Some words on known Akt substrates should be added in the Introduction section : this is essential for the understanding of molecular mechanisms through which Akt1/2 isoforms promote intestinal homeostasis.

Response: We agree with the reviewer, and have revised the introduction accordingly. We indicated the numerous targets of Akt and explained why we focused on FoxO and mTORC1. First, because FOXO targets were at the top of affected genes after Akt1/2 deletion and second because FoxO and mTORC1 are the most evolutionarily conserved downstream effectors of Akt.

2. The introduction is also lacking a paragragh describing the biology of Lgr5+ cells. How do they ultimately generate all epithelial subtypes ? A few words should also describe each epithelial subtypes given the fact that authors will quantify them in the Result section. Generally speaking, the Introduction section is too short.

<u>Response:</u> Although we have briefly mentioned in the Results section that Lgr5⁺ stem cells reside within the crypt and to which epithelial cell lineage they differentiate. We expanded the Introduction section to describe where Lgr5⁺ intestinal stem cells can be found within the crypt base and that they give rise to other differentiated cell types. We also provided information about some of the most critical signaling pathways for the maintenance of stem cells and intestinal tissue homeostasis, including the PI3K/Akt pathway.

3. The authors use Olfm4 as a marker of Lgr5+ stem cells in intestinal crypts. It is also important to note that Olfm4 is a direct target of Notch signaling. Therefore, I think that it would also be interesting to carry out western blot analyses to assess Notch activation in all experimental conditions. Should we expect a defective Notch activation in Akt1/2 double mutants ? This information, if relevant, would add some mechanistical insights into this study.

<u>Response:</u> We appreciate the this insightful point. Although OLFm4 was used as a reliable marker of intestinal stem cells, we analyzed our bulk RNA-seq data to address this mechanistically. We found that several canonical Notch target genes (*Nrarp*, *Dtx1*, *Rfng*, and *Hes2*) were significantly decreased in the small intestine of mice lacking intestinal Akt. Moreover, several Wnt-related genes were reduced, including *Fzd2*, *Wnt6*, *Wnt9b*, *Lypd6*, and *Dand5*. These data suggest that Akt loss dampens two key pathways governing intestinal homeostasis: Notch and WNT (Fig. S2g and Supplementary table 1).

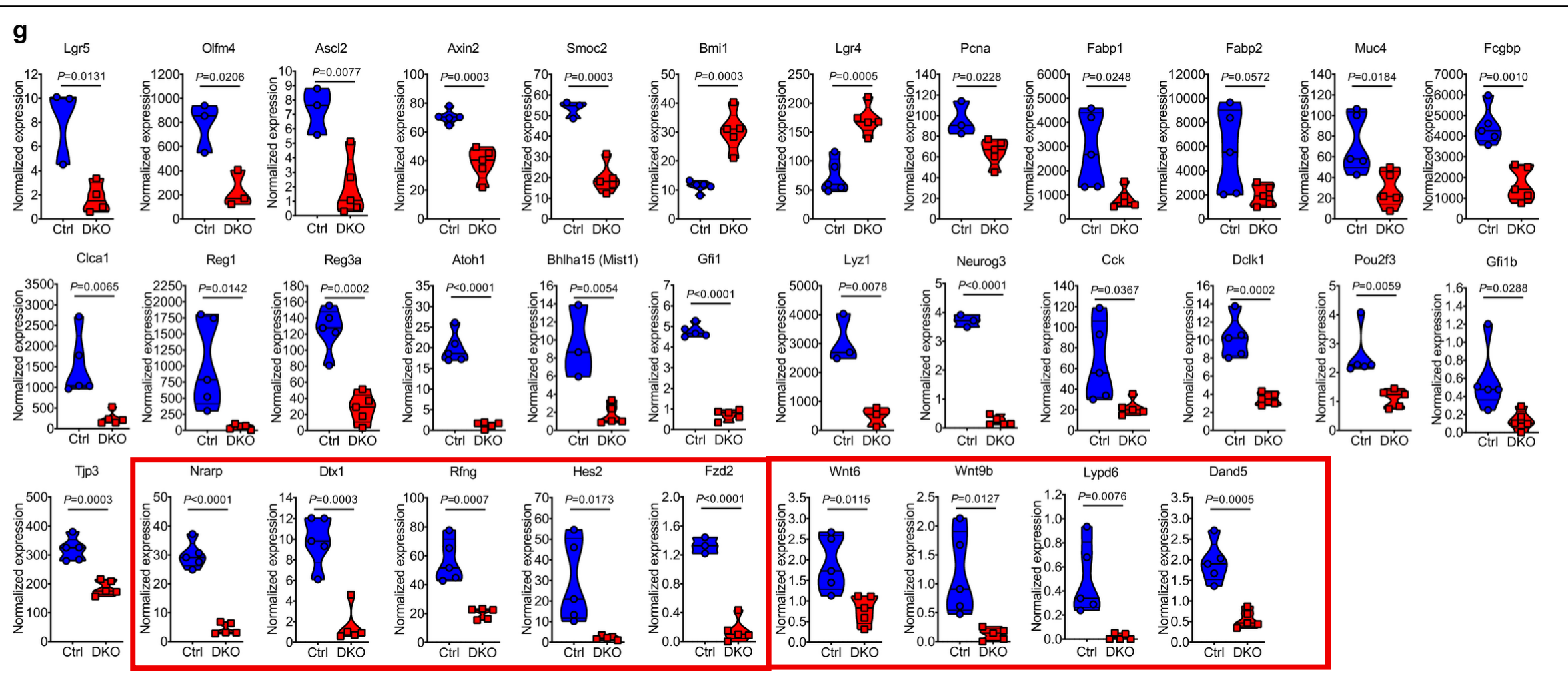

Fig. S2g: NOTCH target genes and Wnt-related genes (boxed) are repressed after the deletion of Akt1 and Akt2.

4. In Figure S2G, the authors nicely show that the loss of both Akt1/2 isoforms in the intestine underlies the severe inflammatory phenotype seen in these mutants. It is important to note that many of these candidates are regulated by NF-kappaB, a pro-

inflammatory and pro-survival family of transcription factors. It is likely that damage in the intestinal architecture seen in Akt1/2 double mutants leads to the activation of Toll-like receptors (TLR) signaling and ultimately to NF-kappaB-dependent gene transcription. The authors should assess NF-kappaB activation by western blot (assessing p65 phosphorylation on Serine 536 is a good marker to assess this).

Response: We appreciate this excellent observation. Our RNA-seq data showed induction of inflammation related genes in Akt-deficient mice (Fig. S2h and Supplementary Table 2), and pathway analysis suggest activation of the TLR/NF-κB axis (Fig. S2i and Supplementary table 3). In addition, our transcription factor analysis indicated enrichment of RELA (p65) and IKBKB (Supplementary table 3), and we also detected increased p65 phosphorylation at Ser-536 after Akt inhibition (Fig. 3e of the revised manuscript). Finally, NF-κB-related transcripts further supported the induction of inflammatory response and immune cell infiltration (Fig. S2j, and Supplementary table 4). Together, our data strongly suggest that induction of NF-κB pathway is a major downstream event that follows Akt loss in the intestinal epithelium leading to intestinal and systemic inflammation.

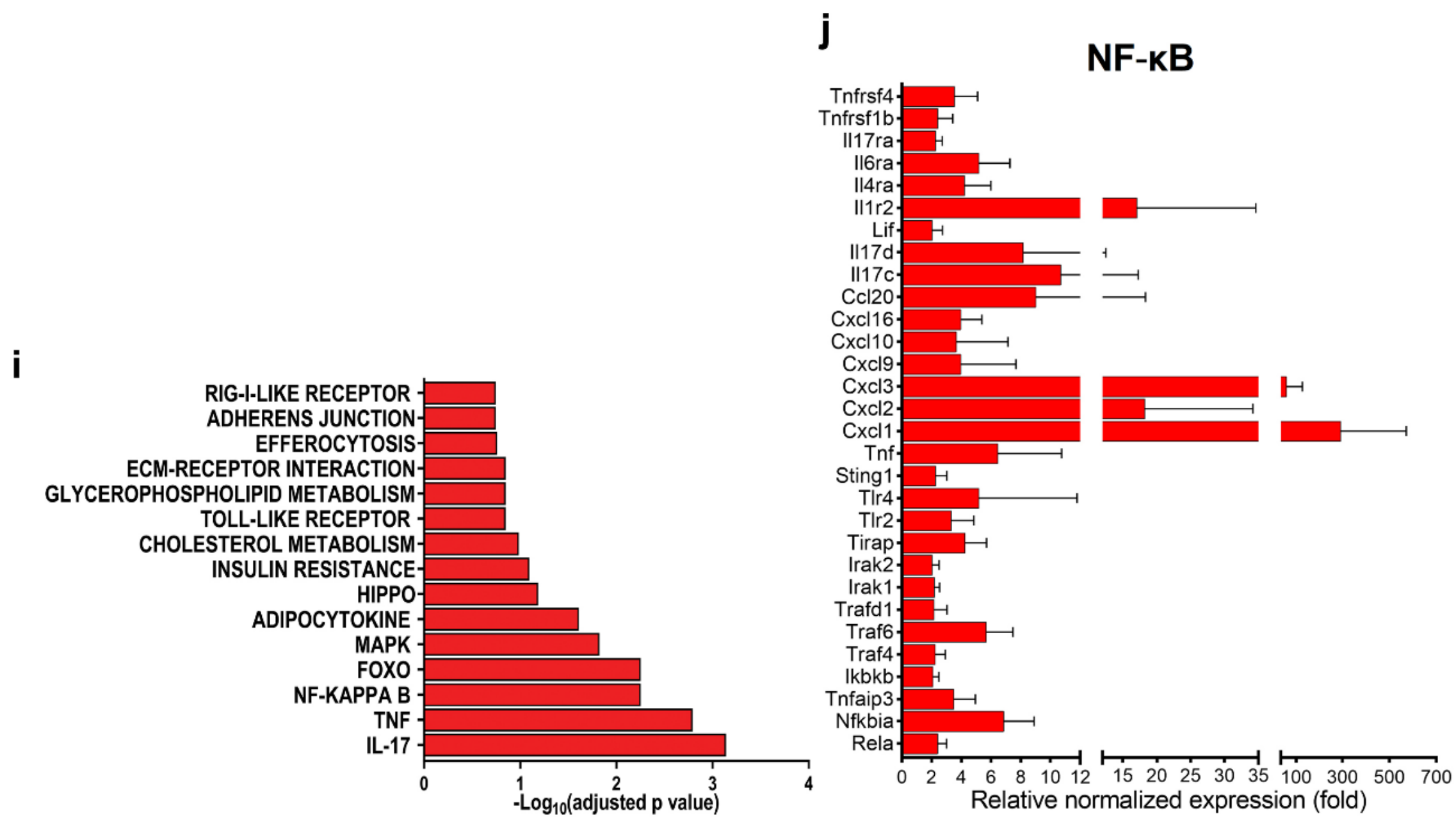

Fig. S2: i) KEGG Pathway Analysis of the most significately (q Value < 0.05) upregulated genes in Akt DKO in comparison to control. j) A list of NF-κB-related transcripts highly elevated in Akt deficient small intestines.

5. For Figure 3E in which several pharmacological Akt inhibitors are tested, the authors assessed S6 phosphorylation. They should also assess nuclear versus cytoplasmic levels of FoxO1 given the fact that FoxO1 is a direct target of Akt. This could actually be an

elegant way to introduce this candidate in the study. We indeed expect FoxO1 to show a distinct subcellular localization in Akt1/2 double mutants versus WT mice as well as in mice treated with Akt inhibitors. Assessing nuclear and cytoplasmic levels of FoxO1 in both experimental models (i.e. in WT/KO mice as well as in mice treated or not with Akt inhibitors) would actually be an additional evidence to prove that phenotypes are comparable. Speaking of this, the authors show an IHC for FoxO1 in Figure 4A but it is difficult to judge this result. The authors should also carry out western blot analyses with nuclear versus cytoplasmic extracts in order to provide a more convincing result.

Response: We agree that FoxO1 localization is an important point. We did not perform nuclear/cytoplasmic fractionation, instead, we performed IHC staining and the results showed predominantly FoxO1 nuclear localization in both the villi and crypt area (Fig. 4a, 4i, S4h, and S8a). Our immunoblotting data confirmed a complete inhibition of FoxO1 phosphorylation in both mouse models of intestine-specific and whole body Akt deletion (Fig. 4b, S4I, and S5a). The IHC findings are consistent FoxO1 activation (see also Figure for the reviewer below).

6. In control mice in which both Akt1/2 isoforms are expressed, we would then expect to see FoxO1 phosphorylation by both kinases. Can we detect FoxO1 phosphorylation in mice under homeostasis ? If so, in which cells ? Lgr5+ cells ? All epithelial subtypes ?
Response: We assessed FoxO1 phosphorylation in control mice by immunoblotting (Fig. 4b, and S4I). These results show in control mice FoxO1 is phosphorylated at Ser-256 (p-FoxO1), however this phosphorylation is almost completely blocked after Akt1 and Akt2 intestine specific and systemic deletion.
We are also showing below more IHC images confirming the nuclear localization of FoxO1 in the small intestine of mice lacking Akt.

Figure for the reviewer: Localization (indicated by arrows) before deletion (cytoplasmic localization) and after exposure to Tam to delete Akt1 and Akt2 (nuclear localization).

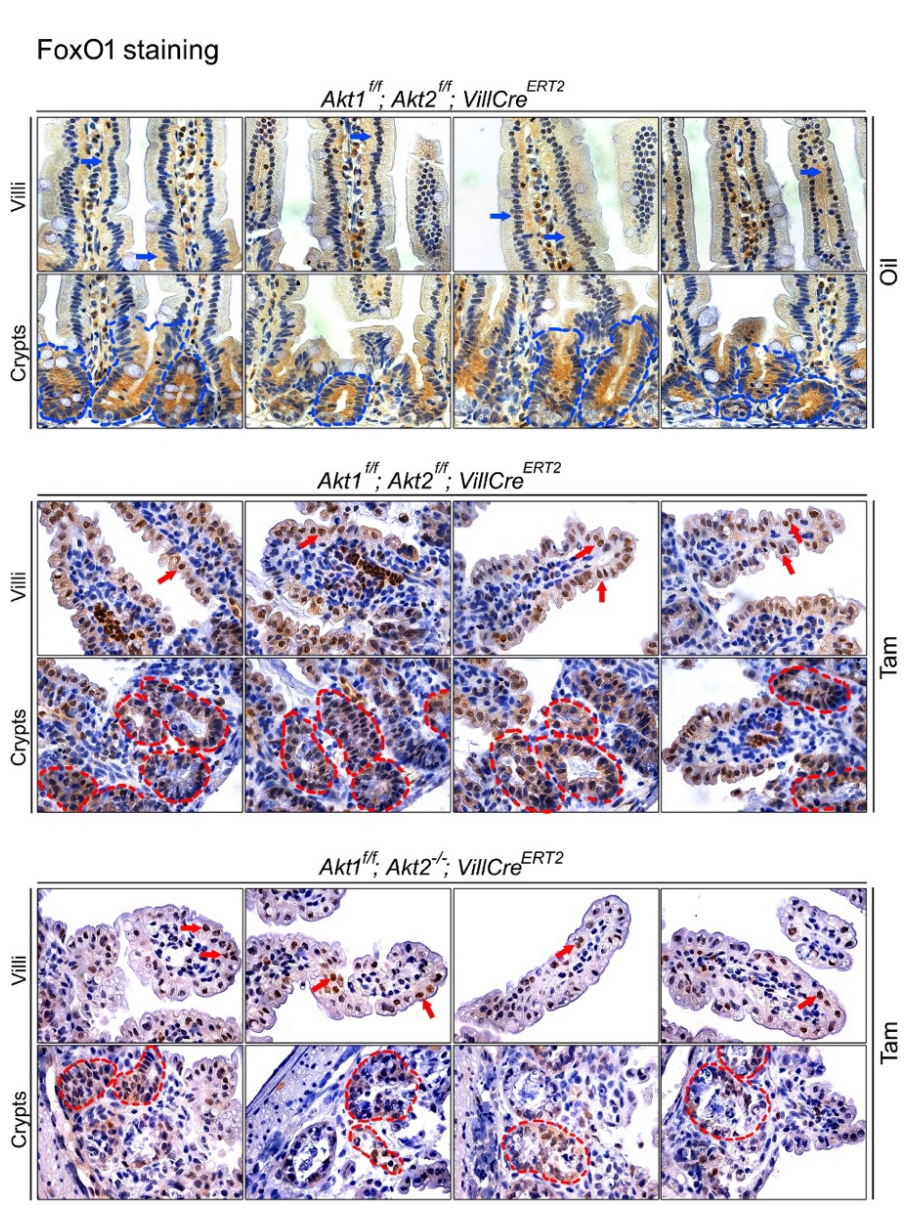

Immunofluorescence studies with co-markers would be nice to do to explore these issues.

Response: Unfortunately, the anti-p-FoxO1 antibody (Cell signaling, Cat# 9461S) that worked well for immunoblotting, did not reliably work for IHC.

7. I did not find any information of mice only lacking FoxO1 in intestinal epithelial cells. This additional control should be carried out as one may say that the genetic inactivation of FoxO1 alone most likely leads to a phenotype. In this context, it is important to note that Chen and colleagues previously published that FoxO1 is important in gut homeostasis and also controls mucus secretion by goblet cells (JEM 2021, PMID 34287641). Findings described in this study should be discussed by authors. In this context, authors of the current study shows that the inactivation of FoxO1 in Akt1/2 double mutants enhances mTORC1 activity (as assessed by anti-pS6 levels by western blot). This important result raises two questions : first, what is happening on mTORC1 activity if we only inactivate FoxO1 in intestinal epithelial cells? This important control has to be done. The second question is on molecular mechanisms through which mTORC1 activation is comparable in Ctrl versus F-TKO mice. Some explanations are required. The current authors previously published a nice study in which functional links between FoxO1 and mTORC1 activity were established. This study should be cited at the time authors introduce data on mTORC1 activity in the manuscript.

Response: We generated FoxO1$^{f/f}$;VillCre^{ERT2} mice, but we did not observe the phenotype described by Chen et al (2021) after FoxO1 deletion. However, we clearly detected the activation of mTORC1 through eleveated p-S6 (Fig. S5 of the revised manuscript). One possible explanation for the discrepancy is that Chen et al used VillCre mice while we deleted FoxO1 in adult mice using VillCre^{ERT2} mice after administration of tamoxifen. This is discussed in the revised manuscript. Notably it was also previously reported that deletion of FoxO1-3 by Lgr5-Cre did not exert the same phenotype (PMID:33147486).

For the comparison between mTORC1 activation detected by p-S6 level, please refer to western blot analysis in (Fig. 4b and S4l) and IHC analysis (Fig. S5b). The data suggest that FoxO1 loss contributes to mTORC1 reactivation observed in F-TKO despite the absence of Akt. Interestingly, these results support our previous finding that FoxO loss activates mTORC1 in Sesn3 and Rictor dependent manner (PMID: 20412774).

8. Changes in total S6 are seen in extracts from Ctrl versus DKO mice (Figure 4A) but those changes are not that dramatic anymore in data illustrated in Figure 6A. This inconsistency should be corrected.

Response: The reviewer probably meant total S6 seen in extracts from Ctrl versus DKO mice (Fig. 4b and Fig. 6b). We looked at pS6 relative to S6 in these figures and observed similar changes (see below). Similar results were obtained in Fig. 5a, 6b and S7l.

Figure 4

b

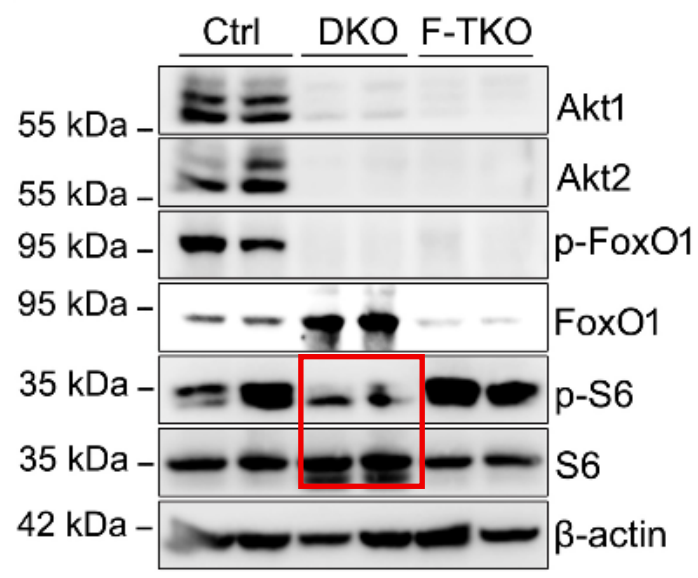

Figure 6

b

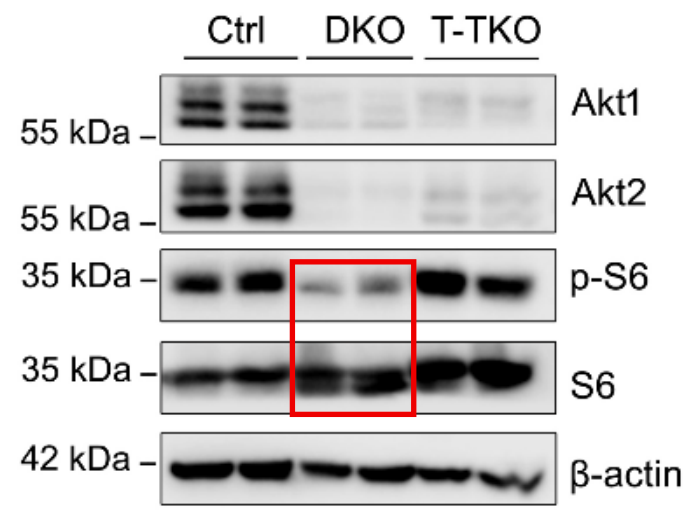

9. Unless I missed something, I have not found Figure S6 in the PDF documents provided by the authors.

Response: We apologize for this error. Figure S6 (Fig. S7 in the revised manuscript) is included in the revised manuscript.

10. Generally speaking, it is not surprising that the loss of both Akt1/2 isoforms leads to cell death and to a severe phenotype in the intestine, given the key role of both isoforms in cell survival. Moreover, it is also expected that FoxO1 deficiency in Akt1/2 double mutants restore the phenotype given the known role of Akt on FoxO1 inhibition through phosphorylation. From a mechanistical point of view, there results, although properly executed, do not provide new mechanisms driving cell survival in the intestine. On the other hand, authors conclude on lane 477 that mTORC1 hyperactivation overrides FoxO1 activity despite its nuclear localization. This observation is much more novel from a mechanistical point of view, but it is sad that authors did not explore this link further. Do we have any evidence that FoxO1 phosphorylation in T-TKO mice would change?

Response: One major objective of the studies is to understand why systemic Akt1 and Akt2 deletion exerts rapid mortality. We unequivocally showed that this is due to intestinal damage, which is mediated by mTORC1 inhibition and the activation of FoxO1. Hyperactivating of mTORC1 by Tsc1 deletion rescued mortality of Akt1 and Akt2 deficient. Likewise, deletion of FoxO1, which activates mTORC1, also rescued mortality. These results suggest that inactivation of mTORC1 by Akt1 and Akt2 deletion is likely the major cause of mortality. As the reviewer mentioned, we also think that it is interesting that mTORC1 hyperactivation in the absence of Akt1 and Akt2 overrides nuclear FoxO1 (not phosphorylated) activity. One potential mechanism is that FoxO1 exert its effect, at least in part, by inhibiting mTORC1 and therefore hyperactivation of mTORC1 could override it. However, to find the exact mechanism is beyond the scope of these studies.

11. Akt1/2 isoforms are expected to target multiple substrates beside FoxO1. Although we understand that FoxO1 is a major candidate acting downstream of Akt1/2 in this study, authors should also discuss the possibility that additional substrates targeted by Akt1/2 may act as effectors of cell survival in the intestine.

Response: Our RNA seq data, together with pathway analysis and transcription factor enrichment, using three independent data sets, revealed that FOXO targets were among the most highly enriched (Fig. S2i). Thus, FOXO and its downstream effectors are highly affected following Akt deletion. Again, two more independent databases suggested FOXO (FoxO1) and P65 (RELA) among the most highly enriched transcription factors (Supplementary table 3). In addition, and as we indicated earlier, we focused on the most evolutionarily conserved downstream effectors of Akt, and indeed FOXO1 deletion rescued the phenotype.

Minor points

1. Molecular weights markers are lacking for western blot analyses.

Response: MW markers were indicated in the revised manuscript.

2. pS6 levels assessed by western blot analyses are illustrated in Figure 4B but these results are not described. The authors should show these western blots later in the paper or describe them at that time.

Response: We now describe these data in the Results section and figure (Fig. 4). We stated that “The results also show activation of mTORC1, as measured by P-S6, in the absence of Akt after FoxO1 removal”.

Reviewer #2 (Remarks to the Author):

Comments to authors

The underlying mechanism of impaired intestinal homeostasis in Akt1/2 dKO mice remains unclear. The authors identified genetic epistasis by which FoxO1 or Tsc1 mediates Akt1/2 dKO-induced intestinal phenotypes. Additionally, the authors showed that genetic deletion of Raptor, a key component of mTORC1, phenocopies Akt1/2 dKO phenotypes. This is a straightforward study that extensively uses genetically engineered mouse models and covers a relatively short phenotypic analysis window of up to 8 days. However, the authors' discovery still has conceptual gaps and requires clarification and additional experimental evidence to support the proposed model better. Addressing the following comments will strengthen the authors' findings.

Major comments

A. Fig. 2F, 4L, . Instead of quantifying the area, the organoid-forming efficiency (OFE, at least up to 3 passages) can better address the impact of Akt1/2 cKO on the integrity and stemness of organoids, as shown in the results using a Lgr5CreERT2 driver. For Supplementary Figure S3A and D, please include additional information, i.e., organoid numbers instead of OFE.

Response: We thank the reviewer for this thoughtful suggestion. We agree that organoid forming efficiency (OFE) is an informative readout of stemness and long-term self-renewal. Initially, we attempted to quantify both metrics, including the number of organoids over a certain number of passages, as well as organoid area, for control and Akt1/2 DKO. However, following Akt1/2 deletion, all crypts/organoids degenerated, and no organoids could be propagated to later passages. Indeed, we have taken images of those "degenerating/dying" organoids after we subcultured them for a second passage, but only a few could form "small cystic enteroids devoid of self-renewal capacity" and never grew beyond that. Thus, serial OFE over two or three passages was not feasible. We believe this complete loss of organoid propagation itself reflects a profound impairment of organoid integrity and stemness in Akt1/2 DKO. In agreement with that, we plated (200–500) crypts per (20–50) µL Matrigel domes in (12–24) well plates, freshly isolated from mice, and found that crypts lacking Akt 1/2, following Tam treatment in vivo, had the same morphology as organoids treated with 4-OHT in vitro. Not a single organoid was formed after Akt deletion; only a few enteroids per well (Fig. 2a-g). Also, please find more images attached below.

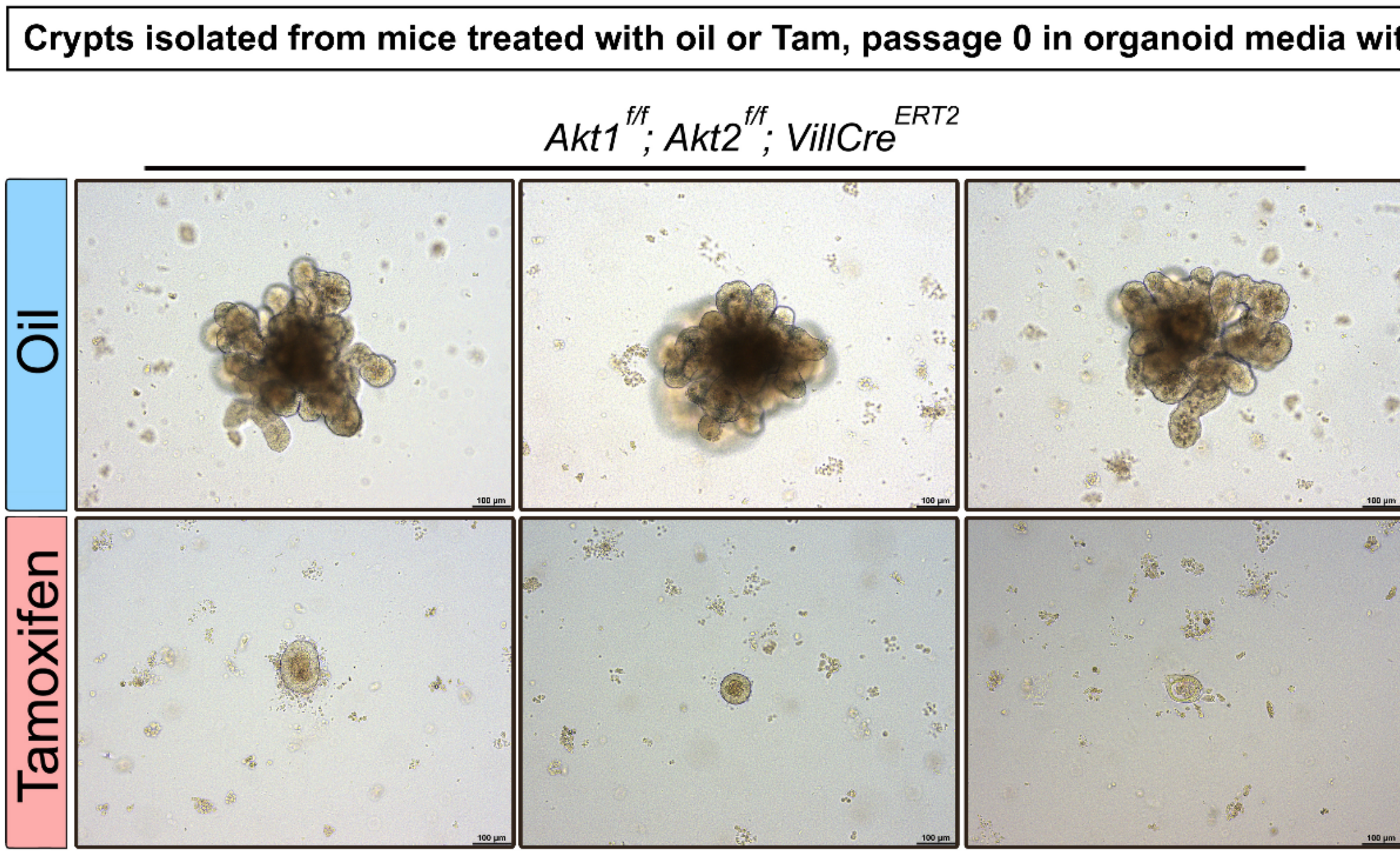

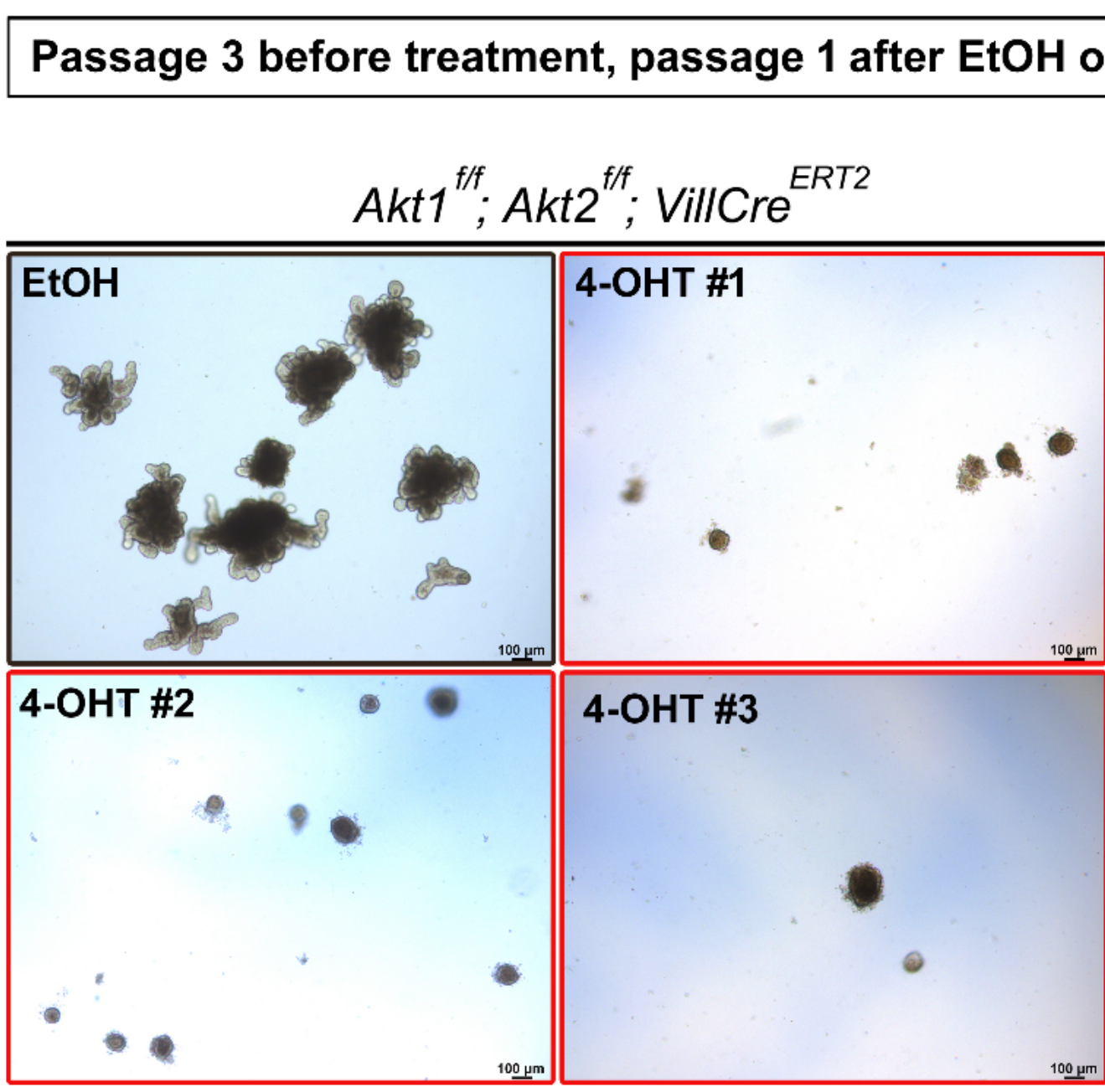

B. Fig. 4A. Please include additional IHC images showing the localization change of FoxO1 from the cytosol to the nucleus. Also, include images from the crypts. This is because Akt1/2 dKO significantly affects ISCs and TAs in the crypts. Moreover, FoxO1 in

Figure 5E does not seem to be expressed in the nuclei or cells in the crypts. Please also improve the FoxO1 IHC to reduce background.

Response: We thank the reviewer for these comments. Since we showed evidence that lethality is FoxO1-dependent, we agree that clarifying FoxO1 localization from the cytosol to the nucleus, particularly in crypt ISCs and TAs, is important. Indeed, we already included higher-magnification images with the upper panels showing villi and lower panels showing crypt regions (Fig. S8a). These images clearly demonstrate cytosolic FoxO1 in the villi and crypts of oil-treated mice and enhanced nuclear FoxO1 in Tam-treated mice. The absence of FoxO1 staining in the cytoplasm of Ctrl mice may suggest the degradation of FoxO1. We included images highlighting the nuclear localization of FoxO1 in Akt1/2 (DKO) and Akt1/2; Tsc1 (T-TKO) (Fig. S8a). We also included two negative controls: the first one (third panels from the left) shows FoxO1 staining only in the non-epithelial cells and a complete loss of signal in the epithelial cells after specific deletion of FoxO1 in the small intestine, and the second negative control (the last panels from the left) shows a lack of FoxO1 staining in all cells of whole-body FoxO1-deleted mice (see also response to point 6 of reviewer 1). These controls highlight the reliability of our staining protocol in maintaining low background and excluding any false positive or negative results. We agree with the reviewer and believe we should replace the FoxO1 stained section in Fig 5e as we did.

Figure S8

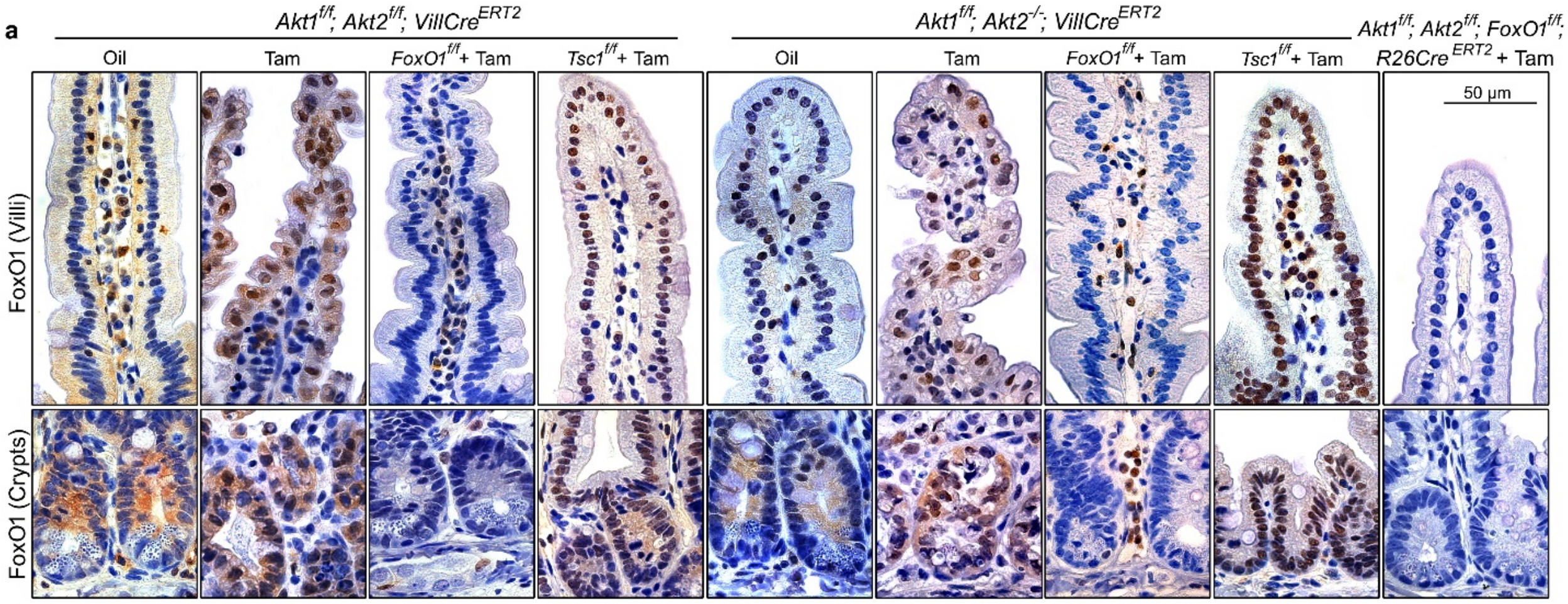

C. Fig. 1J, 3I, 4I, 5E. Muc2, a marker of goblet cells, showed inconsistent results among the phenotypes. Especially, dKO (5E) and dKO (1J) do not match. Based on Fig. 5E, the mucinous cell lineage was not affected by dKO. Also, Raptor cKO failed to inhibit Muc2 expression (Fig. 7F). Explain the inconsistency or repeat the experiments.

Response: We thank the reviewer for highlighting these important points regarding Muc2 staining. However, we would like to clarify the following, regarding the experimental details:

Fig. 1j and 5e (Akt1/2 DKO models): While both show a substantial reduction in the number of goblet cells, they represent distinct genetic models and therefore a side-by-side comparison is not directly comparable. However, Fig. 1j ($Akt1^{f/f}$; $Akt2^{f/f}$; $VillCre^{ERT2}$) demonstrates a significant reduction in the number of goblet cells per villus-crypt. Although Fig. 5e represents a whole-body Akt1/2 KO model, mice lacking Akt1/2 in the whole body showed the same effect. Importantly, in most cases, the fragility of the Akt-null small intestine tissues hindered our ability to completely remove the residual mucus. Therefore, the brown Muc2 signal seen in Fig 5e is likely reflecting extracellular mucus accumulation rather than goblet cells. Furthermore, these $Muc2^{+}$ positive spots lack the characteristic morphology of the goblet-shaped goblet cells so they should not be considered in our quantitative comparison. Regardless, per your request, we have replaced Fig. 5e with a cleaner section that more clearly reveals the expected goblet cell reduction, consistent with Fig. 1j.

Fig. 3l (pharmacological inhibition): these IHC images compare WT mice orally administrated with PI3K or Akt inhibitors vs. vehicle. Our extensive histological examination shows a great reduction in the number of goblet cells among other differentiated cells. However, it should be noted that chemical inhibition transiently disrupts kinase activity, unlike the intestine-specific or whole-body deletion of Akt1/2 in the genetic models. Moreover, the inhibitors were administered orally three to five times per week for two or three weeks, which differs in both duration and kinetics from the permanent gene deletion achieved by the Cre-lox system.

Fig. 4i (two issues need to be addressed here): First, the extreme phenotype in the upper panel intentionally captures a severely affected mouse that survived to endpoint with near-complete loss of differentiated lineages including goblet cells. This biological variability is already captured in our statistics that consistently show a significant reduction across all models of Akt deletion/inhibition in comparison to WT/Ctrl. Second, although we had the most updated version of the figure, we mistakenly submitted an old figure in which Muc2 staining was followed by DclK1 quantification. The corrected version is included in the revised version of the manuscript.

Fig. 7f (Raptor KO): While Muc2 expression appears partially preserved, we have many other tissue sections from severely affected Raptor KO mice showing near-absent villi and goblet cells, confirming mTORC1 essentiality for intestinal homeostasis and survival. An improved IHC image is provided with the revised submission.

D. While the drug-treated mice develop diarrhea and intestinal shortening, these

phenotypes could reflect non-specific systemic toxicity at the chosen dose rather than on-target PI3K/Akt inhibition in the intestinal epithelium. The authors should strengthen this point by providing basic functional readouts of intestinal injury. It would be helpful to include an independent PI3K/Akt inhibitor or include an unrelated compound at a comparably tolerated dose as a negative control for non-specific systemic toxicity.

Response: We agree this is an important concern, however, the drug treatment recapitulated the genetic deletion. Also, western blot analysis shows consistent pathway-specific inhibition across genetic and pharmacological models. Finally, our organoid data recapitulate the in vivo studies regarding induction of cell death and inhibition of stemness and proliferation following Akt deletion or inhibition. Collectively, our data provides evidence that these phenotypes reflect bona fide PI3K/Akt inhibition in the intestinal epithelium rather than off-target effects. Therefore, we believe that no additional experiments are required.

E. The authors suggested that targeting FOXO1 may mitigate gastrointestinal toxicity associated with PI3K/Akt pathway inhibition in cancer therapy. However, because FOXO factors are frequently involved in tumor-suppressive stress and apoptotic programs downstream of PI3K/Akt blockade, systemic FOXO1 inhibition could compromise anti-tumor efficacy or have context-dependent oncogenic consequences. Please clarify the intended clinical scenario (which patient populations/indications for PI3K/Akt inhibitors) and discuss feasibility and safety considerations, including whether a gut-restricted/locally delivered protective strategy would be required and how tumor-specific FOXO1 alterations might affect this approach.

Response: This is a great point. We thank the reviewer for bringing up this translational question. Our data provides proof-of-concept that genetic deletion or pharmacologic inhibition of Akt has deleterious effects on intestinal homeostasis and mice viability, whereas FoxO1 deletion restores intestinal function and survival. We therefore propose that targeting FoxO1 could help mitigate the gastrointestinal toxicity frequently observed in cancer patients receiving PI3K/AKT/mTORC1 targeted therapies. In the revised manuscript (Fig. S5) we showed intestinal deletion of FoxO1 with no apparent phenotype.

F. Given the broad regulatory function of Akt, the rationale for focusing exclusively on FoxO1 remains elusive. The authors should provide a comprehensive list of Akt targets in this model and demonstrate that FoxO1 is a key transcriptional factor in such dKO phenotypes. For instance, instead of showing the selected genes and heatmaps (S2G and S7B, C) from bulk RNA-seq, please perform GSEA, pathway, or regulon analyses to demonstrate activation of the FoxO1-related transcriptional circuit, beyond heatmap clustering. Additionally, intestinal cell lineages (Paneth, mucinous, neuroendocrine, TA, and ISCs [proliferative and quiescent]) can be examined by analyzing the RNA-seq

datasets, strengthening the IHC results. Such unbiased results will strongly justify the authors' choice of FoxO1. This manuscript does not include information about the datasets (e.g., GEO accession number and a token for reviewers) or a data availability statement.

Response: This is true that Akt has multiple downstream effectors. However, the rationale behind focusing on FoxO1 and mTORC1 is that these are the most evolutionarily conserved downstream effectors of Akt. Consistently, in the revised manuscript we analyzed our RNA seq data and found that FoxO signaling pathway is activated and that FoxO1 transcription factor is among the most highly enriched transcription factor (Fig. S2i and Supplementary table 3). We now include in our revised manuscript pathway enrichment and transcription factor analyses.

Minor comments

1. Line 335, Fig. 3I. Until showing the impact of inhibitors on Lgr5-positive cells using Lgr5CreERT2 mice (as a reporter), this sentence incorrectly describes the results. Olfm4 and others are ISC markers. However, the expression of them and Lgr5 could differ. Please revise the sentence.

Response: We agree with the reviewer and this sentence was revised.

2. Discussion can be improved by minimizing redundancy (repetition of results).

We thank the reviewer for this great suggestion and we shortened the Discussion and removed repetitive statements.

3. Please standardize the mouse strain nomenclature throughout the manuscript. The standard notation is C57BL/6 (with "BL" in uppercase) – line 103, 105.

Response: We thank the reviewer for this comment and we standardized this in the revised manuscript.

4. In the introduction (around line 68), the in-text citations appear to be improperly formatted.

Thank you for noticing this, we corrected it in the revised manuscript.

5. Please correct minor typographical issues, for example, "mTROC1" → "mTORC1" and "Tsc1deletion" → "Tsc1_deletion", and scan the manuscript for similar formatting or spacing errors (In the discussion around lines 570 and 573). FoxO1 v. FOXO1

Response: This was corrected in the revised manuscript.

6. Please standardize statistical reporting across all figure legends by explicitly stating what “n” represents and consistently listing the statistical test used and the error-bar definition (SD vs. SEM).

Response: Thank you for this comment. We now specify what n represents, the statistical method, and the error-bar definition in all figure legends.

7. Please include scale bars for each image.

Response: In the revised manuscript all images have scale bars.

8. Fig. 4M. Replace it with better/clearer images.

Response: Thank you for this comment, we replaced Fig. 4M with a clearer image

9. The manuscript states that several key in vivo experiments were performed in male mice, whereas systemic deletion experiments included both sexes. Please clarify the rationale for using male-only cohorts in these experiments.

Response: We found no sex-dependent differences between males and females, therefore to reduce animal use, we focused on only male mice.

10. Figure S6 is missing.

We apologize and it is now included in our submission as Fig. S7.

11. The figure legends and labels need improvement for clarity. Please use markers (arrows, asterisks) and color-coded labels to highlight specific pathological features in IHC H&E images (e.g., Fig.4I/ Fig. S4H).

We thank the reviewer for this comment. We improved the figures and figure legends as suggested.
