# Supplementary material for: Loss of intestinal Akt elicits rapid mortality via intestinal damage which is rescued by mTORC1 activation
Source: Nat Commun. 2026 Sep 23;17:11057. doi: 10.1038/s41467-026-77283-9 (PMC13690743; doi:10.1038/s41467-026-77283-9)
Supplement: Supplementary file 3 [file 41467_2026_77283_MOESM1_ESM.pdf]

Supplementary Information for:

Loss of intestinal Akt elicits rapid mortality via intestinal damage which is rescued by mTORC1 activation

Ahmed Magdy[1, 3, 4*], Hyunsoo Rho[1, 5, 6*], Muzamil Mushtaq[1], William Putzbach[1], Veronique Nogueira[1], Vaibhav Chand[1], and Nissim Hay[1,2,7]

[1]College of Medicine, University of Illinois at Chicago, Department of Biochemistry and Molecular Genetics, Chicago, IL
[2]Jesse Brown VA Medical Center, Research & Development Section, Chicago, IL
*Present address:
[3]Department of Cancer Biology, Dana-Farber Cancer Institute, Boston, MA 02115, USA
[4]Department of Cell Biology, Harvard Medical School, Boston, MA 02115, USA
[5]College of Pharmacy, Graduate School of Pharmaceutical Sciences, Ewha Womans University, Seoul 03760, Republic of Korea.
[6]Graduate Program in Innovative Biomaterials Convergence, Ewha Womans University, Seoul 0 3760, Republic of Korea

Correspondence:

Nissim Hay, nhay@uic.edu

Hyunsoo Rho, hrho@ewha.ac.kr

[7] Lead contact: Nissim Hay, nhay@uic.edu

Figure S1

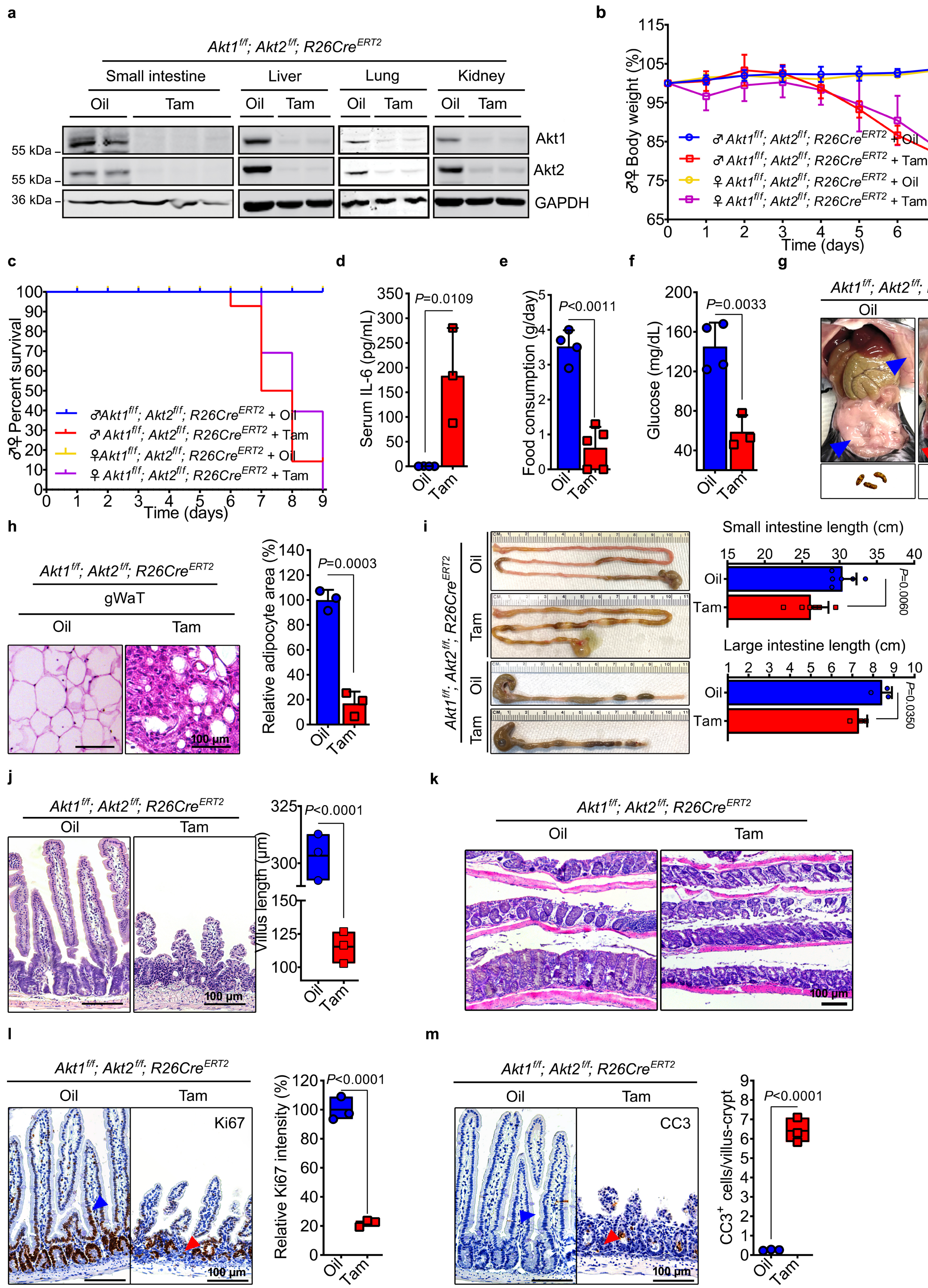
a
Akt1f/f; Akt2f/f; R26CreERT2
Small intestine
Liver
Lung
Kidney
Oil
Tam
55 kDa
36 kDa
Akt1
Akt2
GAPDH
b
♂♀ Body weight (%)
Time (days)
♂ Akt1f/f; Akt2f/f; R26CreERT2 + Oil
♂ Akt1f/f; Akt2f/f; R26CreERT2 + Tam
♀ Akt1f/f; Akt2f/f; R26CreERT2 + Oil
♀ Akt1f/f; Akt2f/f; R26CreERT2 + Tam
c
♂♀ Percent survival
Time (days)
d
Serum IL-6 (pg/mL)
P=0.0109
e
Food consumption (g/day)
P<0.0011
f
Glucose (mg/dL)
P=0.0033
g
h
gWaT
100 μm
Relative adipocyte area (%)
P=0.0003
i
Small intestine length (cm)
P=0.0060
Large intestine length (cm)
P=0.0350
j
Villus length (μm)
P<0.0001
k
l
Ki67
Relative Ki67 intensity (%)
P<0.0001
m
CC3
CC3+ cells/villus-crypt
P<0.0001

Supplementary Figure 1 (Related to Fig. 1) | Systemic deletion of *Akt1* and *Akt2* in C57BL/6 mice causes intestinal dysfunction, massive weight loss and lethality

(a) Western blot showing global deletion of Akt1 and Akt2 in several tissues (small intestine, liver, lung and kidney). GAPDH was used as an internal control. (n=3)

(b) Change in body weight of male and female mice treated with oil or Tam, shown as percent of the initial weight (*n* = 9, 13, 6, 10). Mean ± SD. Two-way mixed-effects model (Tukey's post-hoc multiple comparisons test).

(c) Kaplan-Meier survival curve of ($Akt1^{f/f}$; $Akt2^{f/f}$; $R26Cre^{ERT2}$) oil-injected versus Tam-injected male and female mice (*n* = 12, 12, 9, 9).

(d) Serum IL-6 levels (pg/mL) at endpoint. (*n = 3–4* samples per group). Mean ± SD. Unpaired two-tailed t-test.

(e) Daily food intake 7 days post Tam treatment. (n = 4–5 mice per group). Mean ± SD. Unpaired two-tailed t-test.

(f) Blood glucose levels (mg/dL) demonstrate hypoglycemia in Tam-treated mice (n = 3–4 mice per group). Mean ± SD. Unpaired two-tailed t-test.

(g) Representative images of abdominal organs from oil and Tam-treated mice. (Upper panel) showing fat depletion (red arrowheads), watery cecum, and diarrhea (Lower panel), following systemic deletion of Akt. *(n=3)*

(h) Representative H&E images of gWAT sections showing a significant reduction in adipose area, potentially as a sign of increased fat consumption and enhanced lipolysis, after treatment with Tam compared with control mice (left). Quantification (right). (*n* = 3). Mean ± SD. Unpaired two-tailed t-test.

(i) Gross examination (left) and statistical analysis (right) showing reduction in the length of the small and large intestines in systemic Akt ablation vs. control mice. (n = 3–6 mice per group).

(j) Representative H&E-stained histology images of small intestine from ($Akt1^{f/f}$; $Akt2^{f/f}$; $R26Cre^{ERT2}$) oil-treated compared with tam-treated mice (left). Statistics of villus length (right). Mean ± SD. Unpaired two-tailed t-test.

(k) Representative H&E-stained histology images of large intestines from Akt-null mice in comparison to control group. (n = 3 mice per group).

(l) Representative images of IHC staining in small intestine sections demonstrate a significant reduction in the intensity of Ki67 staining upon Akt systemic deletion compared to control. (n = 3 mice per group). (*n* = 3). Mean ± SD. Unpaired two-tailed t-test. Source data are provided as a Source Data file.

(m) Representative images of IHC staining showing markedly higher number of $CC3^+$ cells within the small intestine crypt area of Akt-depleted mice compared to control. Images are followed by statistical analysis of the relative number of $CC3^+$ cells per crypt (right). (*n* = 3). Mean ± SD. Unpaired two-tailed t-test. Source data are provided as a Source Data file.

Figure S2

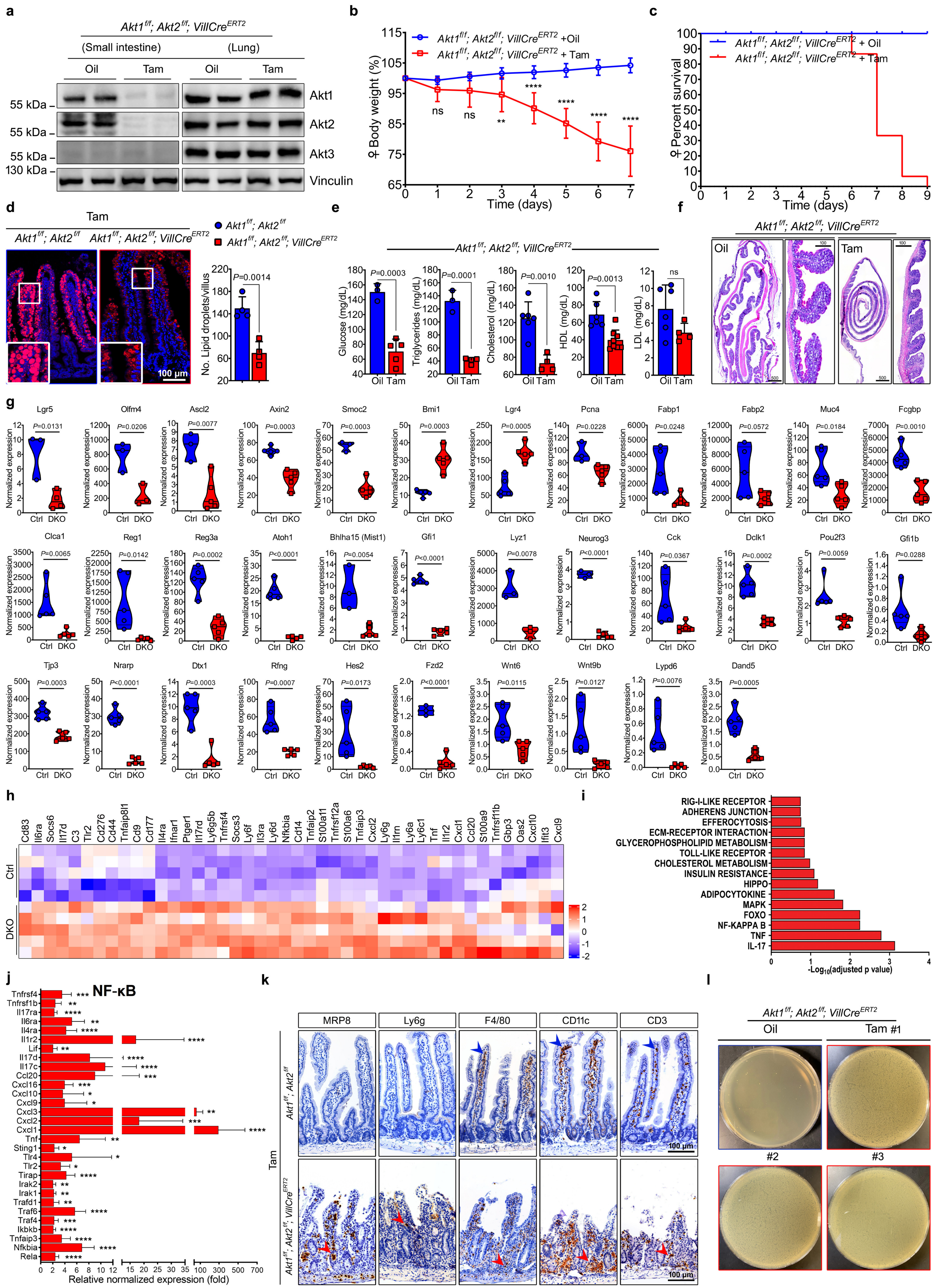

a
Akt1f/f; Akt2f/f; VillCreERT2
(Small intestine)
(Lung)
Oil
Tam
Oil
Tam
55 kDa
55 kDa
55 kDa
130 kDa
Akt1
Akt2
Akt3
Vinculin
b
Akt1f/f; Akt2f/f; VillCreERT2 +Oil
Akt1f/f; Akt2f/f; VillCreERT2 + Tam
♀ Body weight (%)
Time (days)
ns
ns
**
****
****
****
****
c
Akt1f/f; Akt2f/f; VillCreERT2 + Oil
Akt1f/f; Akt2f/f; VillCreERT2 + Tam
♀ Percent survival
Time (days)
d
Tam
Akt1f/f; Akt2f/f
Akt1f/f; Akt2f/f; VillCreERT2
100 μm
Akt1f/f; Akt2f/f
Akt1f/f; Akt2f/f; VillCreERT2
No. Lipid droplets/villus
P=0.0014
e
Akt1f/f; Akt2f/f; VillCreERT2
Glucose (mg/dL)
P=0.0003
Triglycerides (mg/dL)
P=0.0001
Cholesterol (mg/dL)
P=0.0010
HDL (mg/dL)
P=0.0013
LDL (mg/dL)
ns
Oil Tam
f
Akt1f/f; Akt2f/f; VillCreERT2
Oil
Tam
g
Normalized expression
Ctrl DKO
Lgr5 P=0.0131
Olfm4 P=0.0206
Ascl2 P=0.0077
Axin2 P=0.0003
Smoc2 P=0.0003
Bmi1 P=0.0003
Lgr4 P=0.0005
Pcna P=0.0228
Fabp1 P=0.0248
Fabp2 P=0.0572
Muc4 P=0.0184
Fcgbp P=0.0010
Clca1 P=0.0065
Reg1 P=0.0142
Reg3a P=0.0002
Atoh1 P<0.0001
Bhlha15 (Mist1) P=0.0054
Gfi1 P<0.0001
Lyz1 P=0.0078
Neurog3 P<0.0001
Cck P=0.0367
Dclk1 P=0.0002
Pou2f3 P=0.0059
Gfi1b P=0.0288
Tjp3 P=0.0003
Nrarp P<0.0001
Dtx1 P=0.0003
Rfng P=0.0007
Hes2 P=0.0173
Fzd2 P<0.0001
Wnt6 P=0.0115
Wnt9b P=0.0127
Lypd6 P=0.0076
Dand5 P=0.0005
h
Cd83 Il6ra Socs6 Il17d C3 Tlr2 Cd276 Cd44 Tnfaip8l1 Cd9 Cd177 Il4ra Ifnar1 Ptger1 Il17rd Ly6g5b Tnfrsf4 Socs3 Ly6f Il3ra Ly6d Nfkbia Cd14 Tnfaip2 S100a11 Tnfrsf12a S100a6 Tnfaip3 Cxcl2 Ly6g Ifitm Ly6a Ly6c1 Tnf Il1r2 Cxcl1 Ccl20 S100a9 Tnfrsf11b Gbp3 Oas2 Cxcl10 Ifit3 Cxcl9
Ctrl
DKO
i
RIG-I-LIKE RECEPTOR
ADHERENS JUNCTION
EFFEROCYTOSIS
ECM-RECEPTOR INTERACTION
GLYCEROPHOSPHOLIPID METABOLISM
TOLL-LIKE RECEPTOR
CHOLESTEROL METABOLISM
INSULIN RESISTANCE
HIPPO
ADIPOCYTOKINE
MAPK
FOXO
NF-KAPPA B
TNF
IL-17
-Log10(adjusted p value)
j
NF-κB
Tnfrsf4 ***
Tnfrsf1b **
Il17ra ****
Il6ra **
Il4ra ****
Il1r2 ****
Lif **
Il17d ****
Il17c ****
Ccl20 ***
Cxcl16 ***
Cxcl10 *
Cxcl9 *
Cxcl3 **
Cxcl2 ***
Cxcl1 ****
Tnf **
Sting1 *
Tlr4 *
Tlr2 *
Tirap ****
Irak2 **
Irak1 **
Trafd1 **
Traf6 ****
Traf4 ***
Ikbkb ****
Tnfaip3 ****
Nfkbia ****
Rela ****
Relative normalized expression (fold)
k
MRP8
Ly6g
F4/80
CD11c
CD3
Tam
Akt1f/f; Akt2f/f
Akt1f/f; Akt2f/f; VillCreERT2
100 μm
l
Akt1f/f; Akt2f/f; VillCreERT2
Oil
Tam #1
#2
#3

Supplementary Figure 2 (Related to Fig. 1) | Loss of intestinal Akt disrupts nutrient absorption and barrier integrity resulting in inflammation and immune cell infiltration
(a) Western blot analysis demonstrates intestine-specific deletion of Akt1 and Akt2 upon Tam administration, and the absence of Akt3 in small intestine lysates collected from (*Akt1$^{f/f}$*; *Akt2$^{f/f}$*; *VillCre^{ERT2}*). (*n* = 3).

(b) Change in body weight of female mice injected with either oil or Tam, shown as percent of the initial weight. (*n* = 8, 13). Mean ± SD. Two-way mixed-effects model (Sidak's multiple-comparisons test). (ns, $P > 0.05$, *$P \leq 0.05$; **$P \leq 0.01$; ***$P \leq 0.001$; ****$P \leq 0.0001$). Exact P Values are provided in the Source Data File.

(c) Kaplan-Meier survival curve of (*Akt1$^{f/f}$*; *Akt2$^{f/f}$*; *VillCre^{ERT2}*) female mice (n = 15 mice per group).
(d) Representative fluorescence images of Nile Red lipid staining (red) and Hoechst counterstaining (blue), showing lipid droplets (LDs) in proximal jejunum after only 3 days of Tam treatment and 24 h of HFD (left), and quantification of the number of LDs (right). (n = 4 mice per group). Mean ± SD. Unpaired two-tailed t-test.

(e) Blood glucose levels and lipid profile in (*Akt1$^{f/f}$*; *Akt2$^{f/f}$*; *VillCre^{ERT2}*) mice following oil vs. Tam injection (n = 3–8 samples per group). Mean ± SD. Unpaired two-tailed t-test.

(f) H&E-stained colon sections revealing extensive destruction in the colonic epithelium. (n = 3 mice per group).

(g) Analysis of normalized gene expression reveals a significant alteration in the expression levels of markers associated with stem cells *(Lgr5, Olfm4, Ascl2, Axin2, Smoc2, Bmi1, and Lgr4),* transit-amplifying/proliferating cells *(Pcna),* enterocytes *(Fabp1, and Fabp2),* Goblet cells *(Muc4, Fcgbp, Clca1, Reg1, Reg3a, and Atoh1),* Paneth cells *(Bhlha15/MIST-1, Gfi1, and Lyz1),* enteroendocrine cells *(Neurog3, and Cck),* tuft cells *(Dclk1, Pou2f3, and Gfi1b),* Junctional marker *(Tjp3),* Notch pathway components *(Nrarp, Dtx1, Rfng, and Hes2), and* Wnt pathway *(Fzd2, Wnt6, Wnt9b, Lypd6, and Dand5).* Each dot represents an individual mouse. Mean ± SD. Unpaired two-tailed t-test.

(h) Heat map analysis emphasizing inflammation-associated genes. (*n* = 5). Data is presented as Z-scored log^2 Counts Per Million (CPM).

(i) KEGG enrichment analysis of differentially expressed genes between Ctrl and DKO small intestine showing the top 10 enriched KEGG pathways. The bar graph reveals significant enhancement of pro-inflammatory and immune response-related signaling pathways, including IL-17, TNF, NF-κB, and FOXO signaling pathways after Akt deletion.
(j) Bar graph showing a significant increase in the expression of many components of the NF-κB pathway. (*n* = 5). Mean ± SD. Statistical significance is represented on the graph (*q< 0.05), (**q< 0.01), (***q< 0.001), (****q< 0.0001). Exact P values are provided in the Source Data File.

(k) Representative IHC for immune cell markers (MRP8/S100A8: myeloid cells; Ly6g: neutrophils; CD11c: dendritic cells; F4/80: macrophages; CD3: T cells) in small intestine. Red arrowheads indicate the infiltration of various immune cells in the epithelium, while blue arrowheads indicate the immune cells circulating in the blood stream. (*n* = 3).

(l) Bacterial cultures from ileal feces plated on agar demonstrate markedly increased bacterial load in Akt-deficient mice compared to controls. (n=3 mice per group). Source data are provided as a Source Data file.

Figure S3

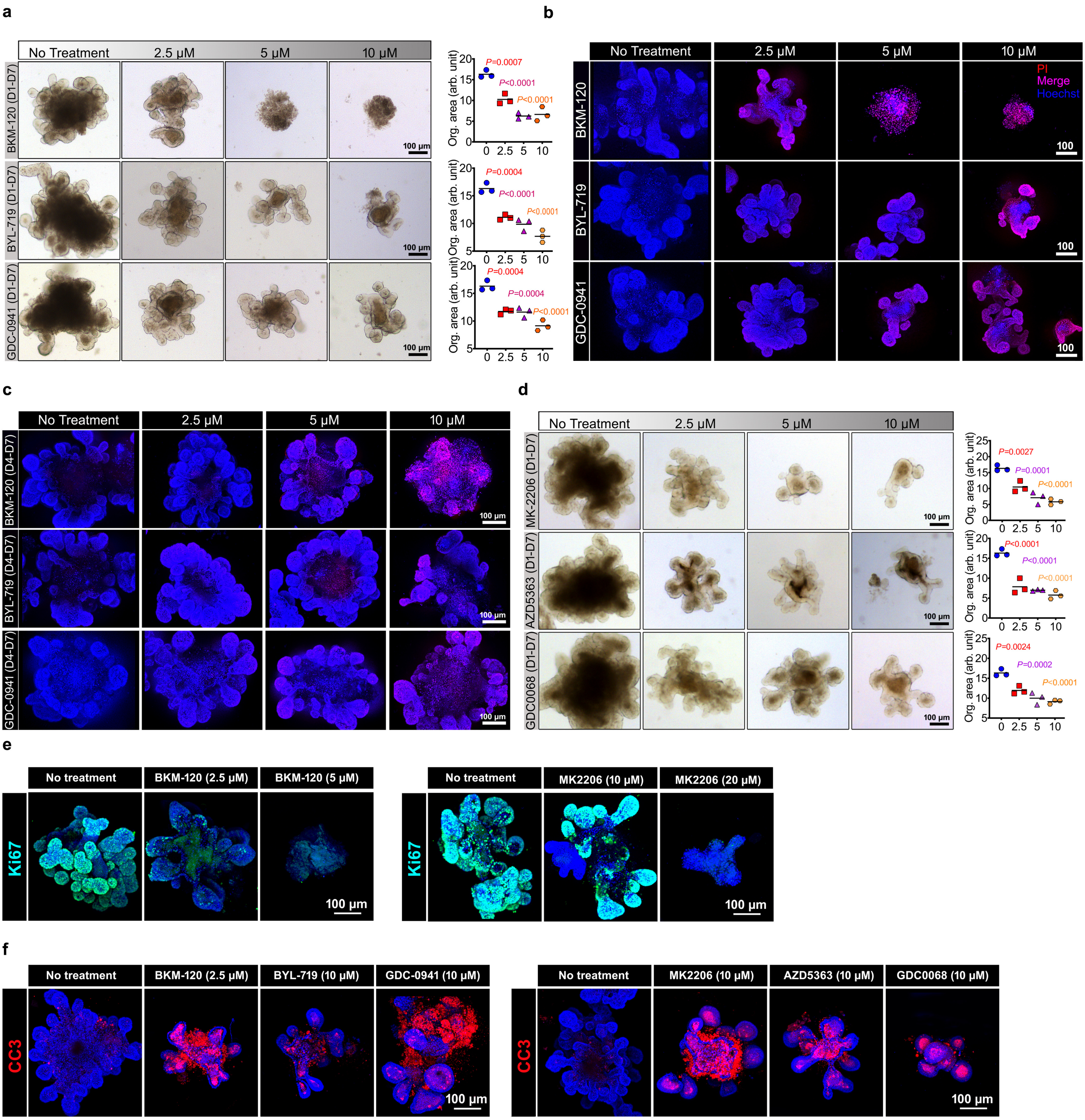
a
No Treatment
2.5 µM
5 µM
10 µM
BKM-120 (D1-D7)
BYL-719 (D1-D7)
GDC-0941 (D1-D7)
100 µm
Org. area (arb. unit)
P=0.0007
P<0.0001
P<0.0001
P=0.0004
P<0.0001
P<0.0001
P=0.0004
P=0.0004
P<0.0001
0 2.5 5 10
b
No Treatment
2.5 µM
5 µM
10 µM
PI
Merge
Hoechst
BKM-120
BYL-719
GDC-0941
100
c
No Treatment
2.5 µM
5 µM
10 µM
BKM-120 (D4-D7)
BYL-719 (D4-D7)
GDC-0941 (D4-D7)
100 µm
d
No Treatment
2.5 µM
5 µM
10 µM
MK-2206 (D1-D7)
AZD5363 (D1-D7)
GDC0068 (D1-D7)
100 µm
Org. area (arb. unit)
P=0.0027
P=0.0001
P<0.0001
P<0.0001
P<0.0001
P<0.0001
P=0.0024
P=0.0002
P<0.0001
0 2.5 5 10
e
No treatment
BKM-120 (2.5 µM)
BKM-120 (5 µM)
No treatment
MK2206 (10 µM)
MK2206 (20 µM)
Ki67
100 µm
f
No treatment
BKM-120 (2.5 µM)
BYL-719 (10 µM)
GDC-0941 (10 µM)
No treatment
MK2206 (10 µM)
AZD5363 (10 µM)
GDC0068 (10 µM)
CC3
100 µm

Supplementary Figure 3 (Related to Fig. 3) | Pharmacological inhibition of the PI3K/AKT pathway impairs organoid formation and crypt maintenance

(a) Representative brightfield images of wild-type (WT) duodenal organoids treated on Day 1 with DMSO or three different doses of PI3K inhibitors for 7 days, followed by quantification of organoid area. (n = 3, at least 5 organoids per treatment).

(b) Propidium iodide (PI, dead cells) and Hoechst (nuclei) staining of WT organoids treated from (Day 1–7) with the indicated doses of the PI3K inhibitors. (*n* = 3).

(c) Representative fluorescence images of PI and Hoechst staining from WT organoids treated with PI3K inhibitors from (Day 4–7). (*n* = 3).

(d) Bright-field images of WT duodenal organoids treated with DMSO or three doses of Akt inhibitors from (Day 1–7) followed by quantification of organoid area. (*n* = 3). (a, d) Data is presented as a scatter dot plot indicating sample mean ± SD. One-way ANOVA followed by Dunnett's post-hoc multiple comparisons test.

(e) Representative images of immunofluorescence for Ki67 in organoids treated with the indicated doses of BKM120 or MK-2206 (Day 3–7). (*n* = 3).

(f) Representative images of immunofluorescence for CC3 in organoids treated from (Day 3–7) with 3 inhibitors of PI3K (left) and 3 inhibitors of Akt (right). (*n* = 3, 3). Source data are provided as a Source Data file.

Figure S4

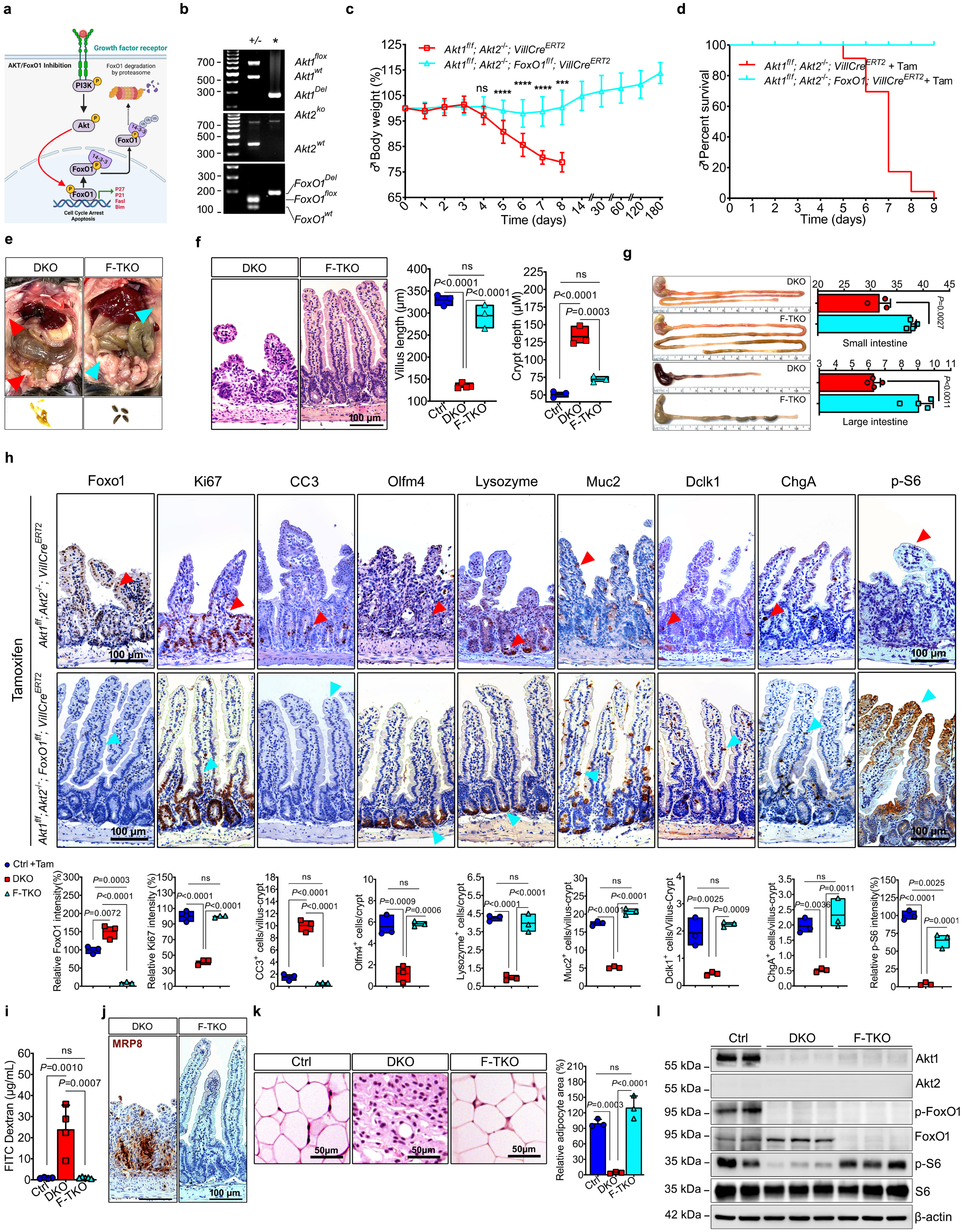

a
Growth factor receptor
AKT/FoxO1 Inhibition
FoxO1 degradation by proteasome
PI3K
Akt
FoxO1
14-3-3
P27
P21
Fasl
Bim
Cell Cycle Arrest
Apoptosis
b
+/-
*
Akt1flox
Akt1wt
Akt1Del
Akt2ko
Akt2wt
FoxO1Del
FoxO1flox
FoxO1wt
c
Akt1f/f; Akt2-/-; VillCreERT2
Akt1f/f; Akt2-/-; FoxO1f/f; VillCreERT2
♂Body weight (%)
Time (days)
ns
****
****
****
***
d
Akt1f/f; Akt2-/-; VillCreERT2 + Tam
Akt1f/f; Akt2-/-; FoxO1f/f; VillCreERT2 + Tam
♂Percent survival
Time (days)
e
DKO
F-TKO
f
DKO
F-TKO
100 μm
Villus length (μm)
Crypt depth (μM)
Ctrl
DKO
F-TKO
P<0.0001
P<0.0001
P=0.0003
ns
g
DKO
F-TKO
Small intestine
Large intestine
P=0.0027
P=0.0011
h
Foxo1
Ki67
CC3
Olfm4
Lysozyme
Muc2
Dclk1
ChgA
p-S6
Tamoxifen
Akt1f/f; Akt2-/-; VillCreERT2
Akt1f/f; Akt2-/-; FoxO1f/f; VillCreERT2
100 μm
Ctrl +Tam
DKO
F-TKO
Relative FoxO1 intensity(%)
Relative Ki67 intensity (%)
CC3+ cells/villus-crypt
Olfm4+ cells/crypt
Lysozyme+ cells/crypt
Muc2+ cells/villus-crypt
Dclk1+ cells/Villus-Crypt
ChgA+ cells/villus-crypt
Relative p-S6 intensity(%)
P=0.0003
P<0.0001
P=0.0072
P=0.0009
P=0.0006
P=0.0025
P=0.0036
P=0.0011
P=0.0001
i
FITC Dextran (μg/mL)
P=0.0010
P=0.0007
ns
j
DKO
F-TKO
MRP8
100 μm
k
Ctrl
DKO
F-TKO
50μm
Relative adipocyte area (%)
P=0.0003
P<0.0001
ns
l
Ctrl
DKO
F-TKO
55 kDa
95 kDa
35 kDa
42 kDa
Akt1
Akt2
p-FoxO1
FoxO1
p-S6
S6
β-actin

Supplementary Figure 4 (Related to Fig. 4) | Intestinal *FoxO1* deletion restores intestinal epithelial integrity, tissue homeostasis, and viability in *Akt2*$^{-/-}$ mice with intestinal Akt1 deletion

(a) Schematic of FoxO1 regulation by Akt. Created in BioRender: https://BioRender.com/e30z608.

(b) Representative PCR results showing deletion of *Akt1* and *FoxO1* in *Akt2* knockout mice following Tam injection (indicated by an asterisk), relative to a heterozygous control sample. (*n* = 3).

(c) The change in body weight demonstrates restoration of normal body weight upon concurrent *FoxO1* deletion (*n* = 23, 12). Mean ± SD. Two-way mixed-effects model (Sidak's multiple-comparisons test).

(d) Kaplan-Meier survival plot of DKO and F-TKO mice. (*n* = 23).

(e) Abdominal photographs showing the differences in body fat content, indicated by arrowhead (upper), and induction of diarrhea in DKO but not T-TKO mice (lower). (*n* = 3).

(f) Representative H&E staining of small intestine (left), showing that disrupted epithelial homeostasis in Akt-null mice is reversed after *FoxO1* deletion. Quantifications (right). (*n* = 3, 4, 3). Data from Ctrl and DKO groups were used again in (Fig. S7f).

(g) (Left) Representative images of small intestine and colon. (Right) Quantification of lengths (cm). (*n* = 5, 4, 3, 4). Mean ± SD. Unpaired two-tailed t-test.

(h) Representative images of IHC staining followed by statistical analysis. Values from Ctrl and DKO were also used in (Fig. S7h). (f,h) floating bars show the minimum, mean, and maximum. One-way ANOVA (Tukey's post-hoc test).

(i) Plasma concentration of FITC-dextran in DKO and F-TKO. Data from Ctrl was used again in S7i. (*n* = 4, 4, 5). Mean ± SD. One-way ANOVA (Tukey's post-hoc test).

(j) Representative images of IHC showing marked increase in MRP8 staining in Akt null compared to F-TKO small intestine. (*n* = 3).

(k) (Left) Representative images of H&E staining of gWAT demonstrate maintained lipid load in adipocytes of F-TKO compared to lipolysis in DKO mice. (Right) statistics. (*n* = 3). Mean ± SD. One-way ANOVA (Tukey's post-hoc test).

(l) Immunoblots demonstrating the deletion of Akt and FoxO1, and the induction of mTORC1 activity following FoxO1 ablation. (*n* = 3). Source data are provided as a Source Data file.

Figure S5

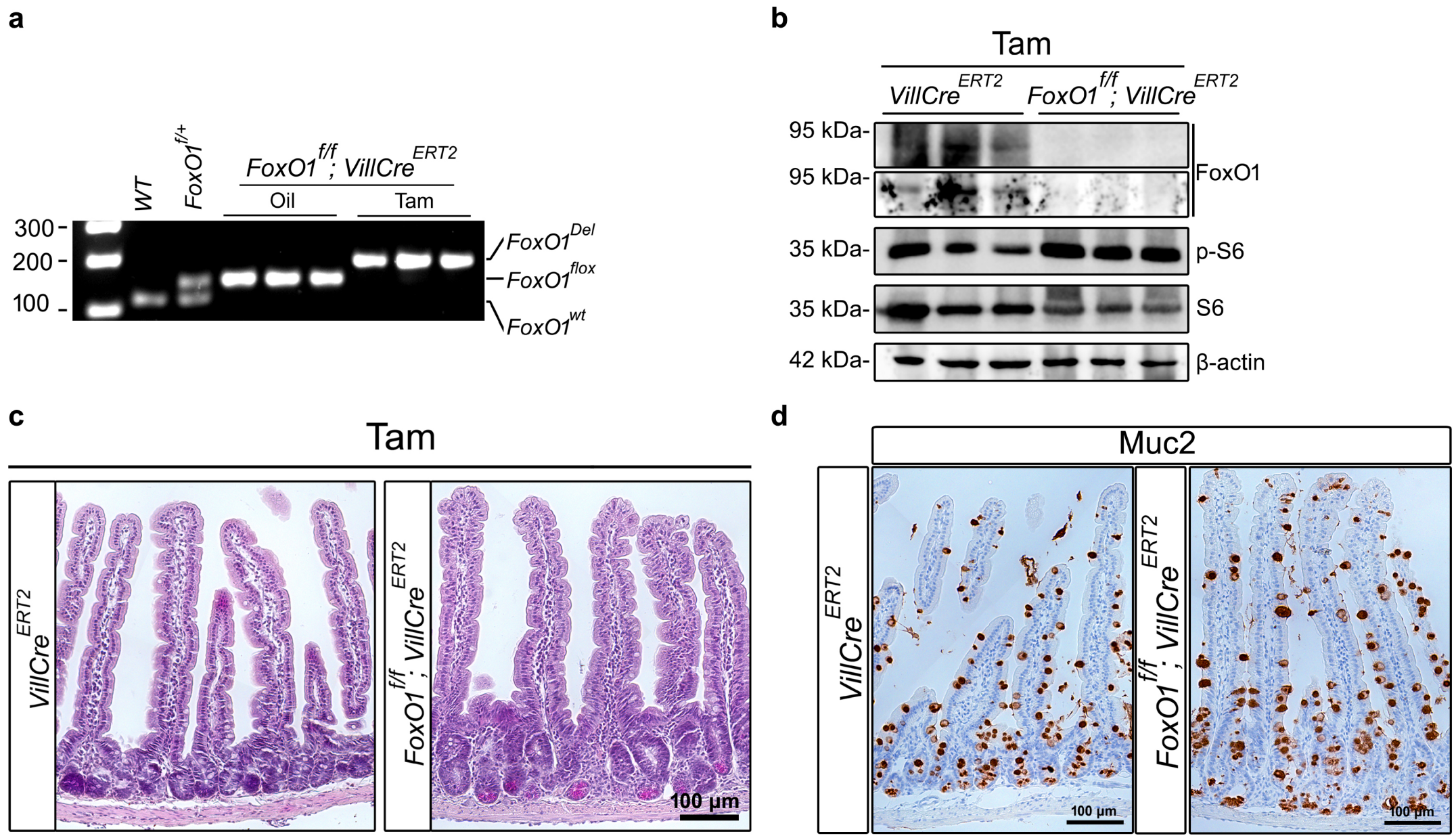

Supplementary Figure 5: Intestinal deletion of FoxO1 did not affect intestinal homeostasis but elevated mTORC1 activity.

(a) PCR analysis of genomic DNA from the small intestine of the indicated genotypes demonstrating efficient deletion of *FoxO1* following Tam treatment. (*n* = 3 mice per group).

(b) Immunoblotting analysis confirming intestinal *FoxO1* deletion and showing p-S6 elevated phosphorylation in mutant mice. (*n* = 3 mice per group).

(c) Representative H&E staining of small intestinal sections showing no alterations in tissue architecture and morphology of FoxO1 deleted mice. (*n* = 3 mice per group).

(d) Representative IHC staining demonstrating no significant alteration in the abundance of goblet cells (Muc2$^+$) between control and FoxO1 deficient mice. (*n* = 3 mice per group).

Figure S6

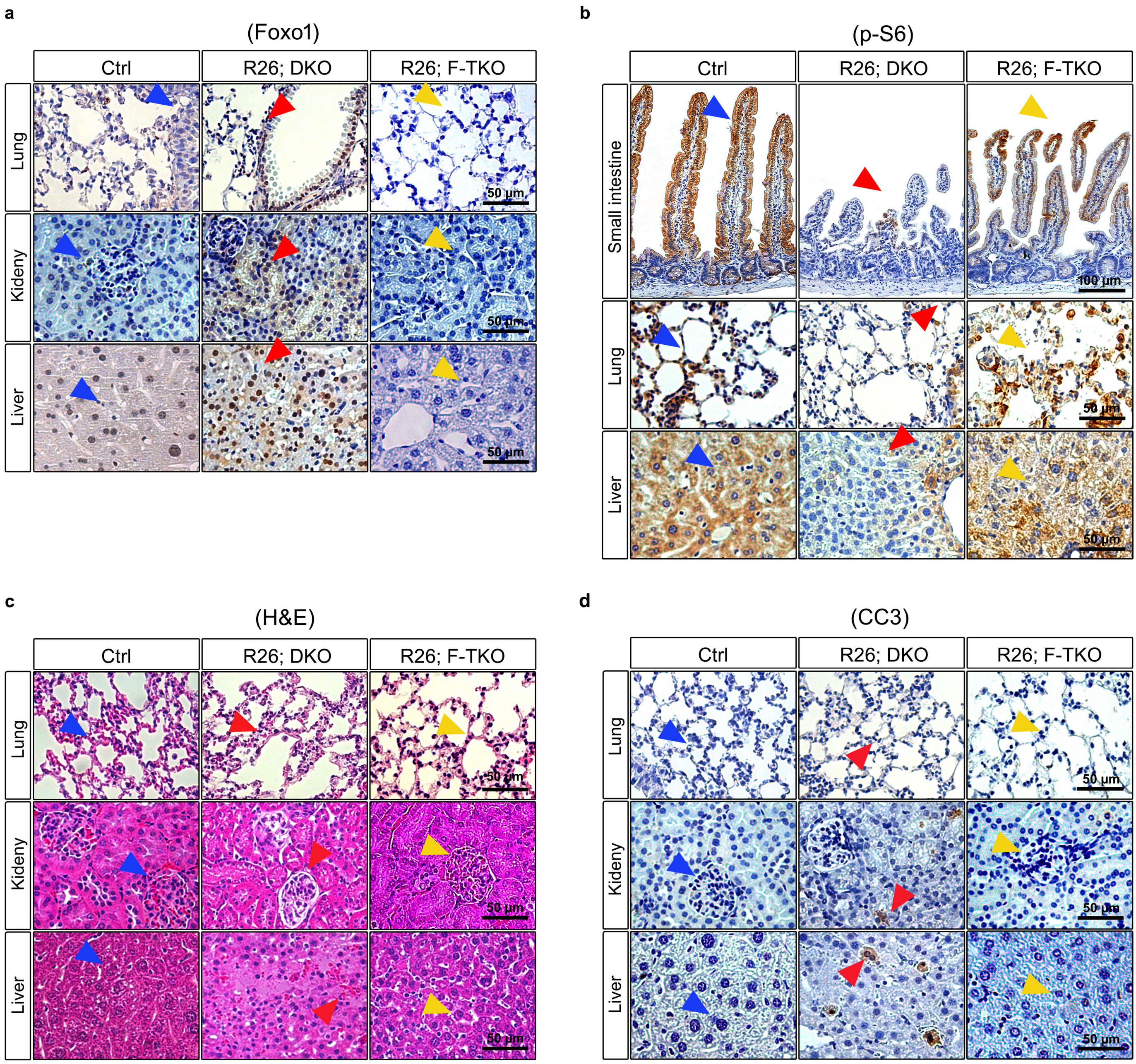

a
(Foxo1)
Ctrl
R26; DKO
R26; F-TKO
Lung
Kideny
Liver
50 μm
b
(p-S6)
Ctrl
R26; DKO
R26; F-TKO
Small intestine
Lung
Liver
100 μm
50 μm
c
(H&E)
Ctrl
R26; DKO
R26; F-TKO
Lung
Kideny
Liver
50 μm
d
(CC3)
Ctrl
R26; DKO
R26; F-TKO
Lung
Kideny
Liver
50 μm

Supplemnatry Figure 6 (Related to Fig. 5) | Systemic *FoxO1* deletion is sufficient to protect from the consequences of *Akt1* and *Akt2* systemic deletion.

(a) Representative images of IHC staining showing localization and accumulation of FoxO1 in the nucleus of the investigated tissues. R26; F-TKO sections confirm the complete deletion of FoxO1. (n = 3 mice per group).

(b) Representative images of p-S6 staining confirming at least partial activation of mTORC1 across various tissues. R26; DKO sections exhibit decreased mTORC1 activity in comparison to Ctrl and R26; F-TKO. (n = 3 mice per group).

(c) Representative H&E-stained sections of lung, kidney, and liver from the indicated genotypes. R26; DKO tissues exhibit histological abnormalities in several tissues including kidney and liver. These abnormalities are reversed in R26; F-TKO tissues. (n = 3 mice per group).

(d) Representative IHC of CC3 staining demonstrating induction of cell death in R26; DKO but not in R26; F-TKO or Ctrl mice.

Figure S7

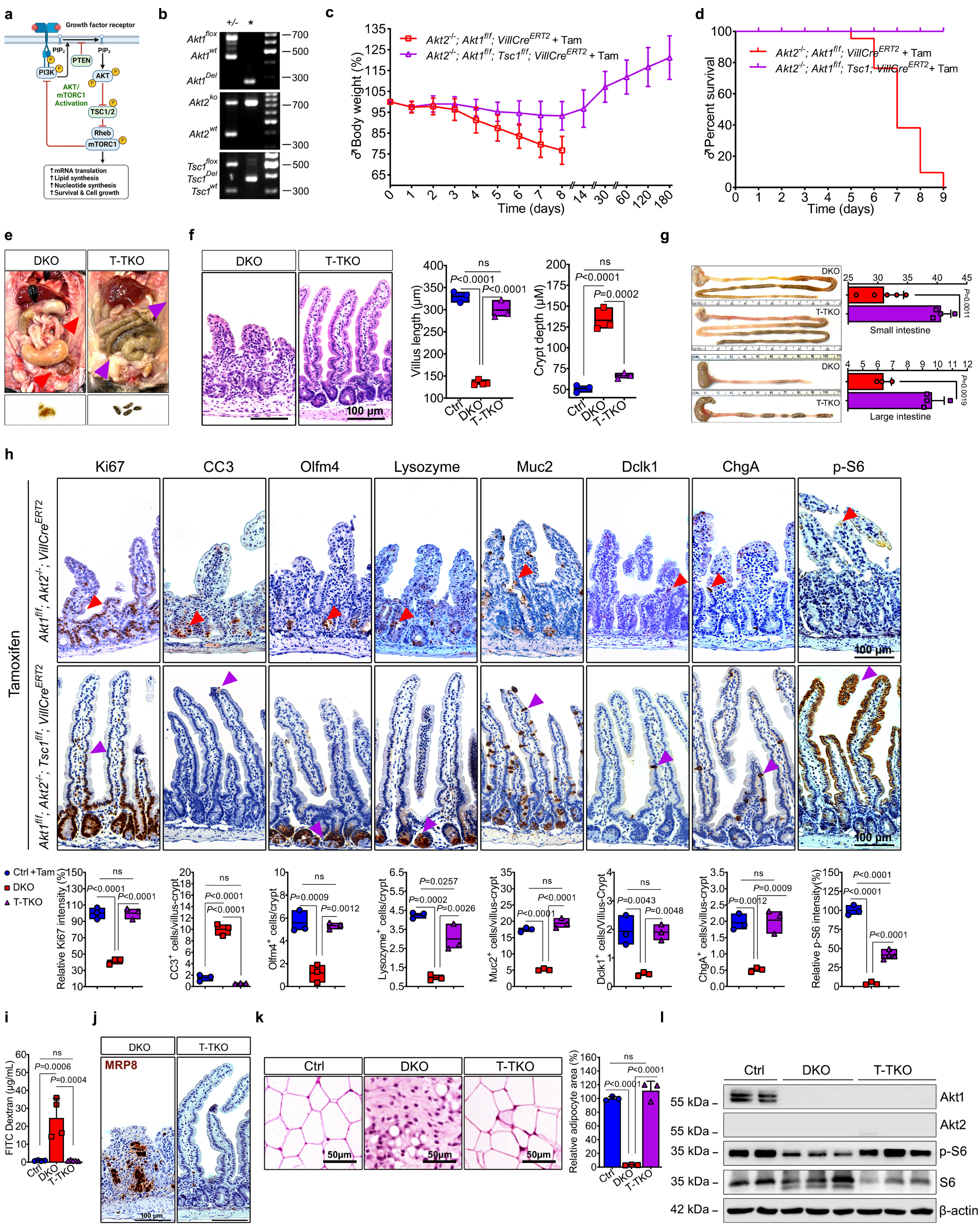

a
Growth factor receptor
PIP2
PIP3
PTEN
PI3K
AKT
AKT/ mTORC1 Activation
TSC1/2
Rheb
mTORC1
↑mRNA translation
↑Lipid synthesis
↑Nucleotide synthesis
↑Survival & Cell growth
b
Akt1flox
Akt1wt
Akt1Del
Akt2ko
Akt2wt
Tsc1flox
Tsc1Del
Tsc1wt
c
Akt2-/-; Akt1f/f; VillCreERT2 + Tam
Akt2-/-; Akt1f/f; Tsc1f/f; VillCreERT2 + Tam
♂Body weight (%)
Time (days)
d
♂Percent survival
Time (days)
e
DKO
T-TKO
f
Villus length (μm)
Crypt depth (μM)
Ctrl
100 μm
g
Small intestine
Large intestine
P=0.0011
P=0.0019
h
Ki67
CC3
Olfm4
Lysozyme
Muc2
Dclk1
ChgA
p-S6
Tamoxifen
Akt1f/f; Akt2-/-; VillCreERT2
Akt1f/f; Akt2-/-; Tsc1f/f; VillCreERT2
Ctrl +Tam
Relative Ki67 intensity (%)
CC3+ cells/villus-crypt
Olfm4+ cells/crypt
Lysozyme+ cells/crypt
Muc2+ cells/villus-crypt
Dclk1+ cells/Villus-Crypt
ChgA+ cells/villus-crypt
Relative p-S6 intensity(%)
i
FITC Dextran (μg/mL)
j
MRP8
k
50μm
Relative adipocyte area (%)
l
Akt1
Akt2
p-S6
S6
β-actin
55 kDa
35 kDa
42 kDa

Supplementary Figure 7: (Related to Fig. 6) | Intestinal activation of mTORC1 by Tsc1 deletion rescues intestinal dysfunction and promotes viability of *Akt2*$^{-/-}$ mice with intestinal Akt1 deletion

(a) Schematic illustration of Akt-dependent activation of mTORC1: Akt phosphorylates TSC2 and inactivates the TSC1/TSC2 complex, a negative regulator of mTORC1 activity, thereby enabling mTORC1 activation. Created in BioRender: https://BioRender.com/q42c071.

(b) PCR analysis of conditional *Akt1* and *Tsc1* excision in *Akt2* germ line deficient mice (*) compared to heterozygous samples (+/-). (*n* = 3).

(c) Change in body weight from Day 0 and over 6 months post Tam injection. (*n* = 23, 17). Mean ± SD. Two-way mixed-effects model (Sidak's multiple-comparisons test).

(d) Kaplan-Meier plot representing survival of *Akt1*$^{f/f}$; *Akt2*$^{-/-}$; *VillCre*^{ERT2} (DKO) and *Akt1*$^{f/f}$; *Akt2*$^{-/-}$; *Tsc1*$^{f/f}$; *VillCre*^{ERT2} (T-TKO). (*n* = 21).

(e) Representative images of internal organs showing fat depletion (red arrowheads), watery cecum, and diarrhea, in DKO but not T-TKO. (*n* = 3).

(f) Representative H&E-stained small intestine sections (left), demonstrating that the disrupted epithelial homeostasis observed in *Akt2*-null mice following intestinal *Akt1* deletion is rescued by *Tsc1* ablation. Quantifications of villus length and crypt depth (right). (*n* = 3, 4, 3).

(g) Representative images showing small and large intestines morphology (Left). Quantification (Right). (*n* = 5, 4, 3, 4). Mean ± SD. Unpaired two-tailed t-test.

(h) Representative IHC staining with the indicated markers followed by quantification. (*n* = 3). (f,h) floating bars show the minimum, mean, and maximum. One-way ANOVA (Tukey's post-hoc test).

(i) Intestinal permeability measure by FITC-dextran assay in the indicated three groups. (*n* = 4, 4, 5) Ctrl data are from S4i. Mean ± SD. One-way ANOVA (Tukey's post-hoc test).

(j) Representative IHC staining showing the infiltration of MRP8^{+} cells in DKO but not T-TKO.

(k) H&E staining of gWAT (Left) and quantification (Right). (*n* = 3). Mean ± SD. One-way ANOVA (Tukey's post-hoc test).

(l) Immunoblots confirming the deletion of Akt isoforms in DKO and T-TKO, and the robust activation of mTORC1 in T-TKO compared to DKO. (*n* = 3). Source data are provided as a Source Data file.

Figure S8

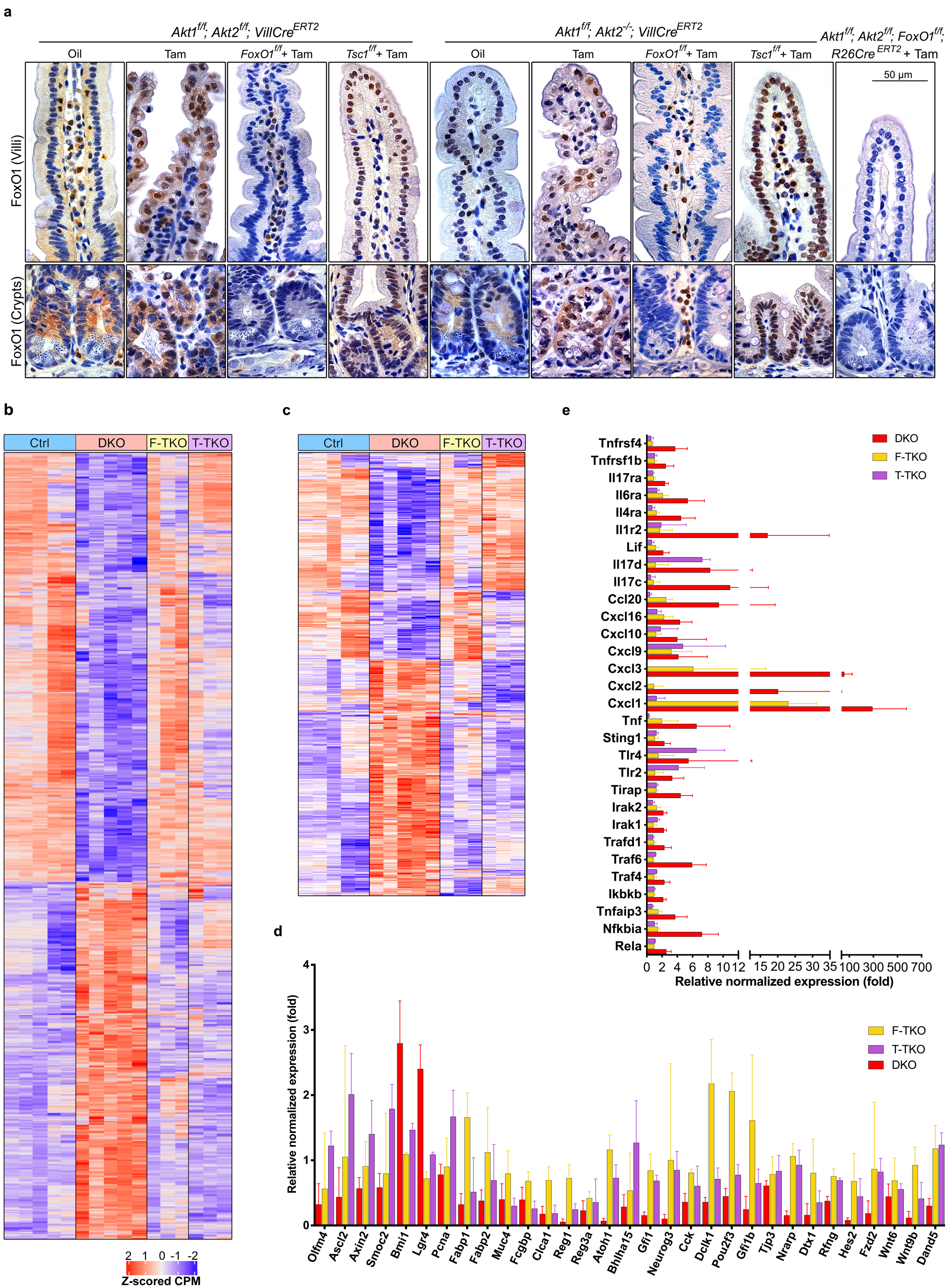

a
Akt1f/f; Akt2f/f; VillCreERT2
Akt1f/f; Akt2-/-; VillCreERT2
Akt1f/f; Akt2f/f; FoxO1f/f; R26CreERT2 + Tam
Oil
Tam
FoxO1f/f + Tam
Tsc1f/f + Tam
50 μm
FoxO1 (Villi)
FoxO1 (Crypts)
b
c
Ctrl
DKO
F-TKO
T-TKO
2 1 0 -1 -2
Z-scored CPM
e
DKO
F-TKO
T-TKO
Tnfrsf4
Tnfrsf1b
Il17ra
Il6ra
Il4ra
Il1r2
Lif
Il17d
Il17c
Ccl20
Cxcl16
Cxcl10
Cxcl9
Cxcl3
Cxcl2
Cxcl1
Tnf
Sting1
Tlr4
Tlr2
Tirap
Irak2
Irak1
Trafd1
Traf6
Traf4
Ikbkb
Tnfaip3
Nfkbia
Rela
Relative normalized expression (fold)
d
Relative normalized expression (fold)
F-TKO
T-TKO
DKO
Olfm4
Ascl2
Axin2
Smoc2
Bmi1
Lgr4
Pcna
Fabp1
Fabp2
Muc4
Fcgbp
Clca1
Reg1
Reg3a
Atoh1
Bhlha15
Gfi1
Neurog3
Cck
Dclk1
Pou2f3
Gfi1b
Tjp3
Nrarp
Dtx1
Rfng
Hes2
Fzd2
Wnt6
Wnt9b
Dand5

Supplementary Figure 8: (Related to Fig. 6) | Localization of FoxO1 in the intestinal cells after Akt1/2 deletion (DKO) and changes in gene expression in DKO, F-TKO, and T-TKO intestines.

(a) IHC analysis demonstrating a complete loss of FoxO1 in intestinal cells following inducible deletion in *Akt1$^{f/f}$; Akt2$^{f/f}$; FoxO1$^{f/f}$; VillCre^{ERT2}* and *Akt1$^{f/f}$; Akt2$^{-/-}$; FoxO1$^{f/f}$; VillCre^{ERT2}* mice, and FoxO1 localization in the nucleus of DKO and T-TKO mice. Results are representative of three independent experiments.

(b) Heat map showing differentially expressed genes in Ctrl, DKO, F-TKO and T-TKO small intestines. (n = 5, 5, 3, 3). Data is presented as Z-scored log^2 Counts Per Million (CPM).

(c) Heat map depicting the only FoxO1 targets in Ctrl, DKO, F-TKO and T-TKO small intestines.

(d and e) Two bar graphs showing the restoration of intestinal homeostasis in Akt lacking mice following FoxO1 or Tsc1 deletion. The results from F-TKO and T-TKO mice demonstrate normalization in the expression of the indicated epithelial cell specific markers and the absence of the inflammatory response stimulated by Akt deletion (DKO). Mean ± SD. Source data are provided as a Source Data file.
